# Regio‐ and Stereoselective Synthesis of 1,1‐Diborylalkenes via Brønsted Base‐Catalyzed Mixed Diboration of Alkynyl Esters and Amides with BpinBdan

**DOI:** 10.1002/ejoc.202000128

**Published:** 2020-03-18

**Authors:** Xiaocui Liu, Wenbo Ming, Xiaoling Luo, Alexandra Friedrich, Jan Maier, Udo Radius, Webster L. Santos, Todd B. Marder

**Affiliations:** ^1^ Institute of Inorganic Chemistry and Institute for Sustainable Chemistry & Catalysis with Boron Julius‐Maximilians‐Universität Würzburg Am Hubland 97074 Würzburg Germany; ^2^ Chongqing Key Laboratory of Inorganic Functional Materials College of Chemistry Chongqing Normal University 401331 Chongqing China; ^3^ Department of Chemistry College of Chemistry Virginia Tech 900 West Campus Drive 24061 Blacksburg Virginia USA

**Keywords:** Boronate esters, Borylation, Cross‐coupling, Synthesis design, Structure elucidation

## Abstract

The NaO*t*Bu‐catalyzed mixed 1,1‐diboration of terminal alkynes using the unsymmetrical diboron reagent BpinBdan (pin = pinacolato; dan = 1,8‐diaminonaphthalene) proceeds in a regio‐ and stereoselective fashion affording moderate to high yields of 1,1‐diborylalkenes bearing orthogonal boron protecting groups. It is applicable to gram‐scale synthesis without loss of yield or selectivity. The mixed 1,1‐diborylalkene products can be utilized in Suzuki–Miyaura cross‐coupling reactions which take place selectivly at the C–B site. DFT calculations suggest the NaO*t*Bu‐catalyzed mixed 1,1‐diboration of alkynes occurs through deprotonation of the terminal alkyne, stepwise addition of BpinBdan to the terminal carbon followed by protonation with *t*BuOH. Experimentally observed selective formation of (*Z*)‐diborylalkenes is supported by our theoretical studies.

## Graphical abstract

Transition‐metal‐free mixed 1,1‐diboration of terminal alkynes with BpinBdan catalyzed by NaO*t*Bu has been developed. The reaction proceeds in a regio‐ and stereoselective fashion affording (*Z*)‐diborylalkenes bearing orthogonal boron protecting groups.

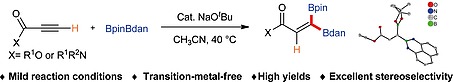

## Introduction

Organoboronic acids and their derivatives have become increasingly of interest due to their widespread application in organic synthesis, materials science, and pharmaceuticals^1^ Alkenylboron compounds have been employed in the stereodefined construction of valuable multisubstituted alkenes including natural products, biologically active molecules, and functional materials.[1h], 2 1,2‐Diborylalkenes are well‐established and are typically synthesized by catalytic diboration of alkynes using Pt,^3^ Pd,^4^ Cu,^5^ Co,^6^ Fe,^7^ Zn^8^ and metal‐free reactions.^9^ Recently, 1,1‐diborylalkenes have emerged as versatile building blocks for the synthesis of multisubstituted alkenes, e.g. the anticancer agent tamoxifen, via selective and stepwise Suzuki–Miyaura couplings.^10^

**Scheme 1:**
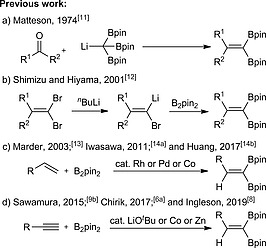
Synthesis of 1,1‐diborylalkenes.

Several approaches have been developed for the synthesis of 1,1‐diborylalkenes. As early as 1974, Matteson et al. described a reaction of carbonyl compounds with triborylmethyllithium, which was prepared by treatment of tetraborylmethane with methyllithium (Scheme 1a).^11^ Shimizu and Hiyama reported that B_2_pin_2_ reacted with alkenylidene‐type lithium carbenoids to afford 1,1‐diborylalkenes via a boron‐based 1,2‐migration. Alkenylidene‐type lithium carbenoids were formed from 1,1‐dibromoalkenes through Li–Br exchange (Scheme 1b).^12^ Later, several transition metal‐catalyzed methods were reported for the synthesis of 1,1‐diborylalkenes using alkenes as starting materials (Scheme 1c). In 2003, during our study of the Rh‐catalyzed dehydrogenative borylation of alkenes, we found that a 1,1‐diborylalkene was formed via a double dehydrogenative borylation of 4‐vinyl anisole with 2 equivalents of B_2_pin_2_.^13^ Subsequently, the Iwasawa and Huang groups reported the use of palladium or cobalt catalysts for the geminal dehydrogenative diboration of terminal alkenes.^14^ In a complementary approach, 1,1‐diborylalkenes can be synthesized from terminal alkynes (Scheme 1d). In 2015, Sawamura developed a Brønsted base (LiO*t*Bu)‐catalyzed 1,1‐diboration of terminal alkynes bearing electron‐withdrawing substituents.[9b] Very recently, more general routes to 1,1‐diborylalkenes from terminal alkynes were developed by the groups of Chirik and Ingleson using cobalt or zinc catalysts.[6a], 8

**Scheme 2:**
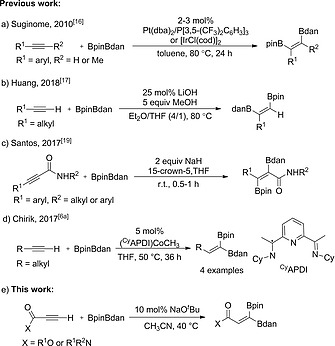
Diboration of alkynes with the unsymmetrical diboron reagent BpinBdan.

Unsymmetrical diboron(4) reagents have been developed and applied in many borylation reactions.[1g], [9e], 15 In 2010, Suginome and co‐workers reported the Pt‐catalyzed regioselective 1,2‐diboration of alkynes^3^ with the unsymmetrical diboron(4) reagent BpinBdan in which the Bdan moiety ends up on the terminal carbon (Scheme 2a).^16^ Later, Huang and Liu reported the diboration of alkyl alkynes with BpinBdan using LiOH as the catalyst in the presence of MeOH. Unlike Suginome's protocol, the Bdan moiety was incorporated at the internal position (Scheme 2b).^17^ Diboration of alkynes to generate *trans*‐configured products are scarce.[4a], [9a], [9c], [9e], 18 Santos and co‐workers developed a transition metal‐free *trans*‐diboration of alkynamides with BpinBdan promoted by NaH. The amide moiety in the substrates acted as a directing group to assist this *trans*‐diboration with excellent selectivities. Bdan and Bpin were exclusively installed on the α‐ and β‐carbon atoms, respectively (Scheme 2c).^19^

The only report on 1,1‐diboration of alkyl alkynes with BpinBdan was performed by Chirik and co‐workers, who synthesized 1,1‐diborylalkenes using 5 mol‐% of (^Cy^APDI)CoCH_3_ as the catalyst (Scheme 2d).[6a] Only 4 examples were reported. Therefore, complementary protocols are necessary. Herein, we report the stereoselective 1,1‐diboration of terminal alkynes with BpinBdan catalyzed by NaO*t*Bu affording 1,1‐diborylalkenes containing two different boryl groups in a regio‐ and stereoselective fashion (Scheme 2e).

We initially studied the reaction using ethyl propiolate **1a** and BpinBdan under a range of conditions (Table 1). Encouragingly, 1,1‐diboryalkene **2a** was obtained in 62 % yield when the reaction was performed in CH_3_CN at 40 °C using LiO*t*Bu as the base catalyst (Entry 1). Analysis of the reaction mixture by GC‐MS showed the presence of a trace amount of by‐product, which might be the *E*‐isomer or 1,2‐isomer, with the same mass and similar fragmentation pattern as **2a**. A screen of Brønsted base catalysts revealed that NaO*t*Bu was superior when compared to LiO*t*Bu, KO*t*Bu, and Cs_2_CO_3_ (Entries 1–4). Weaker organic bases, such as DABCO or Hünig's base (*i*Pr_2_EtN), as catalysts were inefficient (Entries 5 and 6). A control reaction (Entry 7) revealed that NaO*t*Bu was essential for this diboration. Further screening of the amount of NaO*t*Bu, (2 mol‐%, 5 mol‐% and 20 mol‐%), afforded lower yields of **2a** (Entries 8–10). Only a trace of product was obtained when 1 equivalent of NaO*t*Bu was used (Entry 11). A survey of solvents revealed that CH_3_CN was optimal (Entries 12–15). GC‐MS analysis of the crude reaction mixtures showed that **2a** was the main product, indicating excellent regio‐ and stereoselectivities (Figures S1 and S2).

**Table 1:** Optimization of reaction conditions^a^

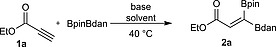			
Entry	Base (mol‐%)	Solvent	Yield of **2a** (%)^b^
1	LiO*t*Bu (10)	CH_3_CN	62 (56)
2	NaO*t*Bu (10)	CH_3_CN	88 (76)
3	KO*t*Bu (10)	CH_3_CN	60 (55)
4	Cs_2_CO_3_ (10)	CH_3_CN	42
5	DABCO (10)	CH_3_CN	< 5
6	DIPEA (10)	CH_3_CN	< 5
7	–	CH_3_CN	0
8	NaO*t*Bu (2)	CH_3_CN	54
9	NaO*t*Bu (5)	CH_3_CN	72 (45)
10	NaO*t*Bu (20)	CH_3_CN	64 (51)
11	NaO*t*Bu (100)	CH_3_CN	< 5
12	NaO*t*Bu (10)	1,4‐dioxane	72 (61)
13	NaO*t*Bu (10)	Et_2_O	65 (52)
14	NaO*t*Bu (10)	MTBE	52 (40)
15	NaO*t*Bu (10)	toluene	60 (51)

a Reaction conditions: In an argon‐filled glove box, **1a** (0.24 mmol, 1.2 equiv.) was treated with base (10 mol‐%), BpinBdan (0.2 mmol) and solvent (2 mL) for 5 h.

b The yields were determined by GC‐MS using *n*‐dodecane as the internal calibration standard; isolated yields are given in parentheses. DABCO: 1,4‐diazabicyclo[2.2.2]octane.

**Figure 1:**
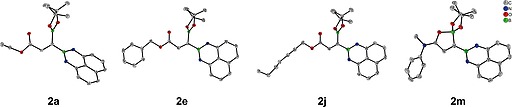
Molecular structures of **2a**, **2e**, **2j**, and **2m**.

With the optimized reaction conditions in hand, the mixed 1,1‐diboration of a variety of alkynoates **1** was tested (Table 2). The model reaction with **1a** afforded **2a** in 76 % isolated yield. Alkoxy substituents ranging from a small methoxy group (**2b**) to much larger *tert*‐butoxy group (**2c**) provided the desired products in high yields. Substrates with cyclohexyloxy (**2d**), benzyloxy (**2e**), furan‐2‐ylmethoxy (**2f**), and naphthalen‐2‐ylmethoxy (**2g**) carbonyl groups, afforded the corresponding products in moderate to high yields (43 %‐78 %). The 1,1‐diboration of phenyl propiolate (**1h**) and naphthalen‐2‐yl propiolate (**1i**) gave products in good yields of 65 % and 75 %, respectively. Notably, in the presence of competing internal alkyne (**2j**) or alkene (**2k** and **2l**) moieties, 1,1‐diboration proceeded at the terminal C≡C bond selectively. Propiolamides **1m** and **1n** were also compatible with this diboration protocol. Increasing the reaction time to 10 h resulted in increased conversion, and the corresponding products were isolated in 87 % and 50 % yields, respectively. Finally, this method enables a convenient gram‐scale synthesis (5 mmol) without loss of yield, as demonstrated for **1a** (**2a**: 1.47 g, 75 %). The structure and stereochemistry of the 1,1‐diborylalkene products was unambiguously confirmed by single‐crystal X‐ray diffraction studies of **2a**, **2e**, **2j**, and **2m** (Figure 1). In contrast to the compounds containing an ester group, the formation of five‐membered rings via O‐B coordination of the amide group with the Bpin moiety was observed in **2m**. Indeed, ^11^B NMR spectroscopy supports the presence of a tricoordinate and a tetracoordinate boron atom in **2m** (29.2, 17.3 ppm) and **2n** (29.9, 15.4 ppm).

**Table 2:**
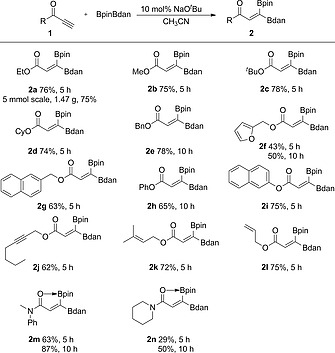
Scope of the mixed 1,1‐diboration of terminal alkynes^a^

a Standard conditions: **1** (0.24 mmol), BpinBdan (0.2 mmol), and NaO*t*Bu (10 mol‐%) in CH_3_CN (2 mL) at 40 °C. Isolated yields.

**Figure 2:**
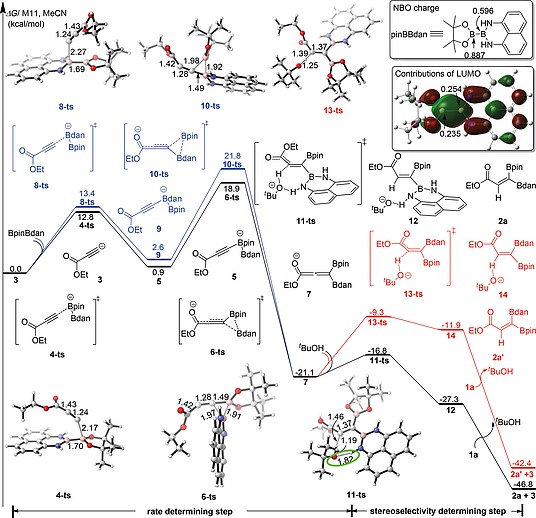
DFT calculations on the mechanism of the mixed 1,1‐diboration of ethyl propiolate (**1a**) at the M11/(6‐311+G(d, p), SMD)//B3LYP‐D3/(6‐31+G(d), SMD) level of theory. Relative free energies (Δ*G*) are given in kcal/mol, and bond lengths are given in Å.

To understand the mechanism and the stereoselectivity of the mixed 1,1‐diboration reaction, we performed a detailed DFT investigation, the results of which are shown in Figure 2. Beginning with the acetylide anion **3** generated via deprotonation of ethyl propiolate (**1a**) by NaO*t*Bu, the more Lewis‐acidic Bpin boron complexes with the alkyne terminal carbon to form anionic adduct **5** via transition state **4‐ts** with a barrier (Δ*G^≠^*) of 12.8 kcal/mol. With cleavage of the B–B bond, Bdan then irreversibly migrates to the alkyne terminal carbon to generate the allenylic intermediate **7** via transition state **6‐ts**, a process which is exergonic by 22.0 kcal/mol. The energy barrier for this step is 18.0 kcal/mol.

Another pathway (blue line) in which the Bdan boron complexes with the acetylide anion **3** followed by 1,2‐migration of Bpin moiety to form **7** is calculated to be unfavorable compared to the above pathway. The relative free energy of **10‐ts** for Bpin migration is higher than that of **6‐ts** by 2.9 kcal/mol. Thus, the preferred pathway for these two steps is nucleophilic attack at the Bpin moiety by **3** followed by 1,2‐migration of Bdan. The contributions of the two boron atoms to the LUMO of BpinBdan were also calculated and they are very similar (0.254 and 0.235). The higher Lewis‐acidity of Bpin due to its more positive NBO charge than Bdan may be responsible for the preference for the formation of anionic adduct **5** which is thermodynamically more stable than **9**. The proton of *t*BuOH, produced by the deprotonation of ethyl propiolate (**1a**) in the initial step, transfers to the internal carbon of allenolate **7** and generates the 1,1‐diborylalkene intermediate **12** or **14** stereoselectively, followed by substrate‐assisted dissociation of *t*BuO^–^ to obtain either product **2a** or **2a'**, respectively. There is a strong driving force of more than 20 kcal/mol for the formation of **2a**/**2a'** (**2a**: 25.7 kJ/mol; **2a'**: 21.3 kJ/mol) starting from **7**. Of both isomers, the experimentally observed product **2a** is clearly the thermodynamically favored one, and lies 4.4 kcal/mol below **2a'**. In addition, the energy barrier leading to **2a** via the transition state **11‐ts**, in which the *t*BuOH attacks from the same side as the Bdan group, is 7.5 kcal/mol lower in energy than that of **13‐ts**, leading to **2a'**, with *t*BuOH attacking from the same side as Bpin. Therefore, **2a** is the main product for both kinetic and thermodynamic reasons, and the acetylide anion **3** generated in this step closes the catalytic cycle. This last step is the stereoselectivity determining step, whereas the rate‐determining step is the Bdan transfer to the alkyne terminal carbon, with the overall activation free energy being 18.9 kcal/mol.

**Figure 3:**
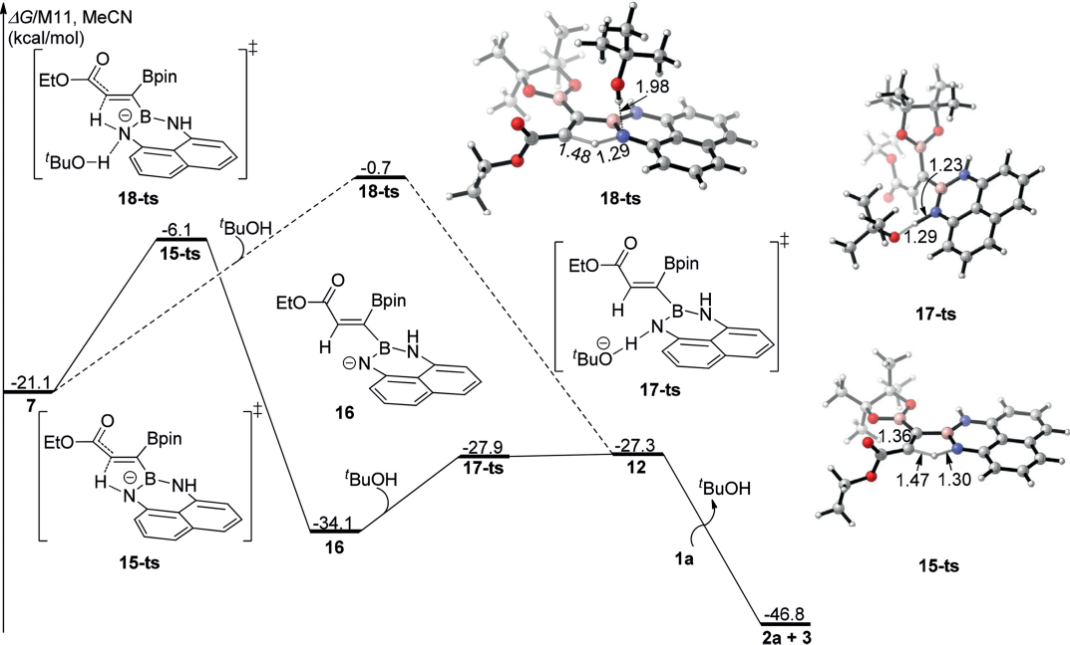
Two alternative pathways for the generation of **2a** calculated at the M11/(6‐311+G(d, p), SMD)//B3LYP‐D3/(6‐31+G(d),SMD) level of theory. Relative free energies (Δ*G*) are given in kcal/mol, and bond lengths are given in Å.

The other two possible pathways to form **2a**, both having higher energy barriers, are shown in Figure 3. The acidic protons on the Bdan group may irreversibly migrate to the carbon of allenyl intermediate **7** via transition state **15‐ts** firstly, followed by fast generation of 1,1‐diborylalkene intermediate **12** by transfer of the *tert*‐butanol proton to nitrogen via **17‐ts**. The relative free energy of **15‐ts** is higher than that of **11‐ts** by 10.7 kcal/mol. Another possible concerted pathway via transition state **18‐ts**, has a barrier of 20.4 kcal/mol, and thus is ruled out. So, the favored pathway leading to **2a** is the one through **11‐ts**.

**Scheme 3:**
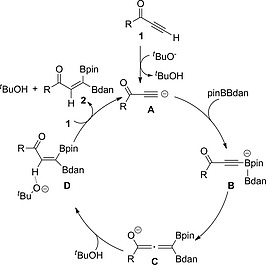
The proposed catalytic cycle for the mixed 1,1‐diboration of terminal **1**.

On the basis of our DFT calculations and experimental results (for details see SI, Part V), a possible catalytic cycle for the NaO*t*Bu‐catalyzed mixed 1,1‐diboration of alkynes is shown in Scheme 3. Deprotonation of the alkyne by the Brønsted base NaO*t*Bu generates acetylide **A**,^20^ which was evidenced by the stoichiometric reaction with *n*BuLi. Species **A** reacts with BpinBdan, in which the carbanion attacks the Bpin moiety selectively vs. the less electrophilic Bdan group, to form an sp^2^‐sp^3^ alkynyl borate intermediate **B**.[1g], [15k], [15l], 21 Then, 1,2‐migration of the Bdan moiety in **B** to the terminal carbon atom of the alkyne occurs to generate allenolate intermediate **C**. Proton transfer to the internal carbon of alkyne produces, stereoselectively, 1,1‐diborylalkene intermediate **D** and, finally, product **2** is obtained with the release of *tert*‐butanol and regeneration of acetylide anion **A**.

**Scheme 4:**
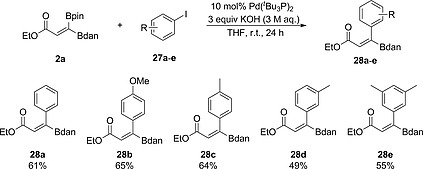
Chemoselective Suzuki–Miyaura cross‐coupling reactions of **2a** with aryl iodides. Isolated yields are given.

The synthesis of 1,1‐diborylalkenes bearing two different boryl groups (Bpin and Bdan) is particularly attractive because their differing reactivities allow selective and stepwise Suzuki–Miyaura cross‐couplings.[6a], 16, 17, 19, 22 Thus, Suzuki–Miyaura coupling of **2a** with aryl iodides **27a**–**e**, gave the corresponding (*Z*)‐alkenylboronates **28** as single isomers in moderate yields (Scheme 4). A 2D NOESY study of compound **28a** supports our assignment of the (*Z*)‐configuration (Figure S8).

## Conclusion

In conclusion, we have developed a simple and highly selective mixed diboration of terminal alkynes with BpinBdan catalyzed by inexpensive and readily available NaO*t*Bu. Diverse 1,1‐diborylacrylates and 1,1‐diborylacrylamides with two different boron protecting groups, which were difficult to prepare previously, were obtained in moderate to high yields with excellent atom‐economy. Our DFT calculations suggest a catalytic cycle of acetylene deprotonation, BpinBdan stepwise addition followed by protonation. Finally, Suzuki–Miyaura cross‐coupling reactions of the products occurred exclusively at the Bpin position affording trisubstituted alkenes.

https://www.ccdc.cam.ac.uk/services/structures?id=doi:10.1002/ejoc.202000128 1959477 (for **2a**), 1969050 (for **2e**), 1969051 (for **2j**), and 1969052 (for **2m**) contain the supplementary crystallographic data for this paper. These data can be obtained free of charge from http://www.ccdc.cam.ac.uk/structures.

## Supporting information

Supporting Information
