# Supplementary material for: Regio‐ and Stereoselective Synthesis of 1,1‐Diborylalkenes via Brønsted Base‐Catalyzed Mixed Diboration of Alkynyl Esters and Amides with BpinBdan
Source: European J Org Chem. 2020 Mar 18;2020(13):1941–6. doi: 10.1002/ejoc.202000128 (PMC7187442; doi:10.1002/ejoc.202000128)
Supplement: Supplementary file 1 — Supporting Information [file EJOC-2020-1941-s001.pdf]

European Journal of Organic Chemistry

Supporting Information

Regio- and Stereoselective Synthesis of 1,1-Diborylalkenes via Brønsted Base-Catalyzed Mixed Diboration of Alkynyl Esters and Amides with BpinBdan

Xiaocui Liu, Wenbo Ming, Xiaoling Luo,* Alexandra Friedrich, Jan Maier, Udo Radius,* Webster L. Santos,* and Todd B. Marder*

Table of Contents

I. General Information 2

II. Preparation of Starting Materials 3

Preparation of propiolates and propiolamides 3

Characterization of alkynes (**1**) 3

III. Optimization of the Reaction Conditions 6

IV. Substrate Scope 8

Experimental procedures 8

Characterization of products (**2**) 11

V. Investigations Concerning the Reaction Mechanism 17

Sequential stoichiometric reaction 17

Deuterium labelling experiment 18

Computational methods 20

VI. Synthetic Applications of the Mixed 1,1-Diborylalkene 24

Characterization of products (**28**) 26

VII. NMR Spectra 29

VIII. Cartesian Coordinates for All Optimized Geometries 64

IX. Single-crystal X-ray Diffraction 87

X. References 93

I. General Information

Reagents were purchased from Alfa-Aesar, Aldrich, ABCR or VWR, and were checked for purity by GC-MS and/or ^{1}H NMR spectroscopy and used as received. BpinBdan was synthesized from B_2pin_2 according to a literature procedure.[1] HPLC grade solvents were argon saturated and dried using an Innovative Technology Inc. Pure-Solv Solvent Purification System, and further deoxygenated using the freeze-pump-thaw method. $CDCl_3$ was purchased from Cambridge Isotope Laboratories. All manipulations were performed in an argon-filled glove box.

Automated flash chromatography was performed using a Biotage® Isolera Four system, on silica gel (Biotage SNAP cartridge KP-Sil 10 g and KP-Sil 25 g). Commercially available, precoated TLC plates (Polygram® Sil G/UV254) were purchased from Machery-Nagel. The removal of solvent was performed on a rotary evaporator *in vacuo* at a maximum temperature of 40 °C.

GC-MS analyses were performed using an Agilent 7890A gas chromatograph (column: HP-5MS 5% phenyl methyl siloxane, 30 m, ∅ 0.25 mm, film 0.25 μm; injector: 250 °C; oven: 80 °C (2 min), 80 °C to 180 °C (20 °C min^{-1}), 180 °C to 280 °C (50 °C min^{-1}), 280 °C (5 min); carrier gas: He (1.2 mL min^{-1})) equipped with an Agilent 5975C inert MSD with triple-axis detector operating in EI mode and an Agilent 7693A series auto sampler/injector. Elemental analyses were performed on a Leco CHNS-932 Elemental Analyzer in our Institute. High-resolution mass spectra were recorded using a Thermo Fischer Scientific Exactive Plus Orbitrap MS system (ASAP, ESI or HESI probe).

NMR spectra were recorded at ambient temperature using Bruker DRX-300 (^{1}H, 300 MHz; ^{13}C{^{1}H}, 75 MHz; ^{11}B, 96 MHz) or Bruker Avance 500 NMR (^{1}H, 500 MHz; ^{13}C{^{1}H}, 126 MHz; ^{11}B, 160 MHz; ^{19}F, 471 MHz) spectrometers. ^{1}H NMR chemical shifts are reported relative to TMS and were referenced *via* the residual proton resonance of the deuterated solvent ($CDCl_3$: 7.26 ppm) whereas ^{13}C{^{1}H} NMR spectra are reported relative to TMS *via* the carbon signal of the deuterated solvent ($CDCl_3$: 77.00 ppm). ^{11}B NMR chemical shifts

are quoted relative to $BF_3 \cdot Et_2O$ as the external standard. ^{19}F NMR chemical shifts are quoted relative to $CFCl_3$ as the external standard.

II. Preparation of Starting Materials

Preparation of propiolates and propiolamides

The propiolates and propiolamides were synthesized according to published procedures[2] and their ^{1}H and ^{13}C NMR spectra are in accordance with those in the literature (**4-1d**,[3] **4-1e**,[3] **4-1i**[4] and **4-1l**[5]).

Characterization of alkynes (1)

Furan-2-ylmethyl propiolate (1f, CAS number: 1447925-21-4)

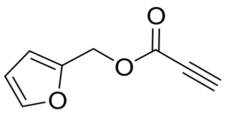

Flash column chromatography (hexane: ethyl acetate = 99:1) afforded the product **1f** (66%), pale yellow liquid.

^{1}H NMR (300 MHz, $CDCl_3$): δ = 7.43 (dd, J = 2, 1 Hz, 1H), 6.47 – 6.44 (m, 1H), 6.36 (dd, J = 3, 2 Hz, 1H), 5.16 (s, 2H), 2.91 (s, 1H).

^{13}C{^{1}H} NMR (75 MHz, $CDCl_3$): δ = 152.2, 148.0, 143.6, 111.6, 110.6, 75.4, 74.2, 59.3.

HRMS (ASAP): m/z for $C_8H_6O_3$ $[M+H]^+$ calcd: 151.0345, found: 151.0344.

Naphthalen-2-ylmethyl propiolate (1g, CAS number: 77119-37-0)

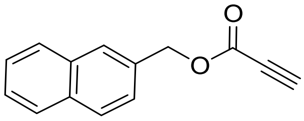

Flash column chromatography (hexane: ethyl acetate = 99:1) afforded the product **1g** (76%), white solid, m.p.: 124.4 °C.

^{1}H NMR (300 MHz, $CDCl_3$): δ = 7.96 – 7.71 (m, 4H), 7.57 – 7.43 (m, 3H), 5.39 (s, 2H), 2.91 (s, 1H).

^{13}C{^{1}H} NMR (75 MHz, $CDCl_3$): δ = 152.5, 133.3, 133.1, 131.9, 128.5, 128.0, 127.9, 127.7, 126.5, 126.4, 125.9, 75.1, 74.5, 68.0.

HRMS (ASAP): m/z for $C_{14}H_{10}O_2$ $[M+H]^+$ calcd: 211.0709, found: 211.0706.

Phenyl propiolate (1h, CAS number: 60998-71-2)

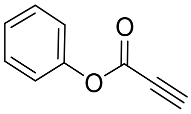

Flash column chromatography (hexane: ethyl acetate = 99:1) afforded the product **1h** (73%), pale yellow liquid.

^{1}H NMR (300 MHz, $CDCl_3$): δ = 7.46 – 7.37 (m, 2H), 7.32 – 7.23 (m, 1H), 7.20 – 7.11 (m, 2H), 3.08 (s, 1H).

^{13}C{^{1}H} NMR (75 MHz, $CDCl_3$): δ = 150.9, 149.8, 129.6, 126.6, 121.2, 76.8, 74.2.

HRMS (ASAP): m/z for $C_9H_6O_2$ $[M+H]^+$ calcd: 147.0441, found: 147.0438.

Hept-2-yn-1-yl propiolate (1j)

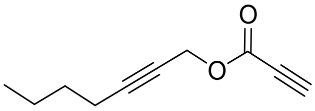

Flash column chromatography (hexane: ethyl acetate = 99:1) afforded the product **1j** (65%), colorless liquid.

^{1}H NMR (300 MHz, $CDCl_3$): δ = 4.76 (s, 2H), 2.92 (s, 1H), 2.22 (tt, *J* = 7, 2 Hz, 2H), 1.60 – 1.32 (m, 4H), 0.90 (t, *J* = 7 Hz, 3H).

^{13}C{^{1}H} NMR (75 MHz, $CDCl_3$): δ = 152.0, 88.9, 75.4, 74.1, 72.6, 54.5, 30.3, 21.9, 18.4, 13.5.

HRMS (ASAP): m/z for $C_{10}H_{12}O_2$ $[M+H]^+$ calcd: 165.0910, found: 165.0908.

3-Methylbut-2-en-1-yl propiolate (1k, CAS number: 118741-76-7)

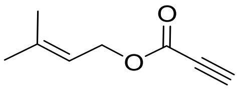

Flash column chromatography (hexane: ethyl acetate = 99:1) afforded the product **1k** (55%), pale yellow liquid.

^{1}H NMR (300 MHz, $CDCl_3$): δ = 5.41-5.34 (m, 1H), 4.67 (d, *J* = 8 Hz, 2H), 2.86 (s, 1H), 1.75 (s, 3H), 1.71 (s, 3H).

^{13}C{^{1}H} NMR (75 MHz, $CDCl_3$): δ = 152.7, 140.7, 117.3, 74.7, 74.4, 63.0, 25.7, 18.0.

HRMS (ASAP): m/z for $C_8H_{10}O_2$ [M] calcd: 138.0675, found: 138.0674.

1-(Piperidin-1-yl)prop-2-yn-1-one (1n, CAS number: 82038-68-4)

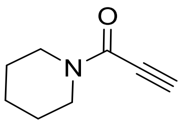

Flash column chromatography (hexane: ethyl acetate = 99:1) afforded the product **1n** (77%), pale yellow solid, m.p.: 104.4 °C.

^{1}H NMR (300 MHz, $CDCl_3$): δ = 3.66 (t, J = 6 Hz, 2H), 3.52 (t, J = 6 Hz, 2H), 3.10 (s, 1H), 1.68-1.40 (m, 6H).

^{13}C{^{1}H} NMR (75 MHz, $CDCl_3$): δ = 151.5, 78.9, 75.5, 48.0, 42.2, 26.2, 25.1, 24.3.

HRMS (ASAP): m/z for $C_8H_{11}NO$ $[M+H]^+$ calcd: 138.0913, found: 138.0911.

III. Optimization of the Reaction Conditions

Table S1: Screening of bases for the mixed 1,1-diboration of alkynes.[a]

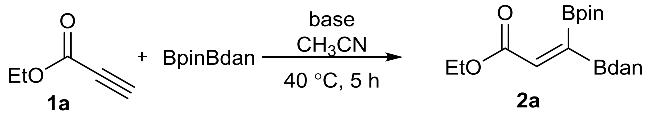

Entry	Base	Yield **2a** (%)[b]
1	LiOMe	0
2	LiOAc	0
3	DMAP	5
4	K_2CO_3	0
5	Li_2CO_3	0
6	KF	0

[a] Standard conditions: in an argon-filled glove box, BpinBdan (0.2 mmol), **1a** (0.24 mmol, 1.2 equiv), base (10 mol%), CH_3CN (2 mL), at 40 °C for 5 h. [b] The yield was determined by GC-MS using *n*-dodecane as the internal calibration standard.

Table S2: Screening of the amount of base for the mixed 1,1-diboration of alkynes.[a]

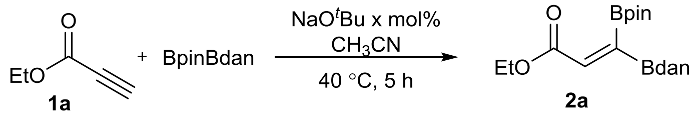

Entry	NaO*t*Bu (x mol%)	Yield **2a** (%)[b]
1	40	40
2	60	24
3	80	<5

[a] Standard conditions: in an argon-filled glove box, BpinBdan (0.2 mmol), **1a** (0.24 mmol, 1.2 equiv), base (x mol%), CH_3CN (2 mL), at 40 °C for 5 h. [b] The yield was determined by GC-MS using *n*-dodecane as the internal calibration standard.

Table S3: Screening of reaction time for the mixed 1,1-diboration of alkynes.[a]

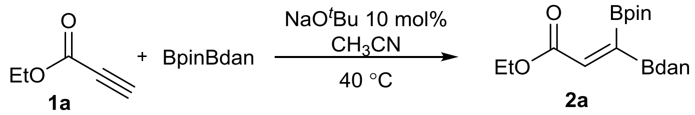

Entry	Time (h)	Yield **2a** (%)[b]
1	1	53
2	2	56 (54)
3	3	64 (60)
4	4	70
5	10	86 (72)

[a] Standard conditions: in an argon-filled glove box, BpinBdan (0.2 mmol), **1a** (0.24 mmol, 1.2 equiv), NaO^{t}Bu (10 mol%), CH_3CN (2 mL), at 40 °C. [b] The yield was determined by GC-MS using *n*-dodecane as the internal calibration standard. Isolated yields are given in parentheses.

IV. Substrate Scope

Experimental procedures

General procedures for products 2.

In a 10 mL thick-walled reaction tube equipped with a magnetic stirring bar, BpinBdan (59 mg, 0.2 mmol), base (2 mg, 0.02 mmol) and CH_3CN (2 mL) were added. Then, alkynes **1** (0.24 mmol) were added and the tube was sealed with a crimped septum cap. The reaction was heated at 40 °C under argon for 5 h. The reaction mixture was then diluted with Et_2O (4 mL) and filtered through a plug of celite (Ø 3 mm × 8 mm) in air with copious washing (Et_2O). The solvents were removed *in vacuo*, and the residue was purified by flash column chromatography on silica gel.

Experimental procedure for the synthesis of 2a on a gram scale.

In a 10 mL thick-walled reaction tube equipped with a magnetic stirring bar, BpinBdan (1.470 g, 5 mmol), base (48 mg, 0.5 mmol) and CH_3CN (10 mL) were added. Then, ethyl propiolate **1a** (588 mg, 6 mmol) was added and the tube was sealed with a crimped septum cap. The reaction was heated at 40 °C under argon for 5 h. The reaction mixture was then diluted with Et_2O (30 mL) and filtered through a plug of celite (Ø 3 mm × 8 mm) in air with copious washing (Et_2O). The solvents were removed *in vacuo*, and the residue was purified by flash column chromatography on silica gel (hexane: ethyl acetate = 90:10) giving **2a** as a yellow solid (1.47 g, 75%).

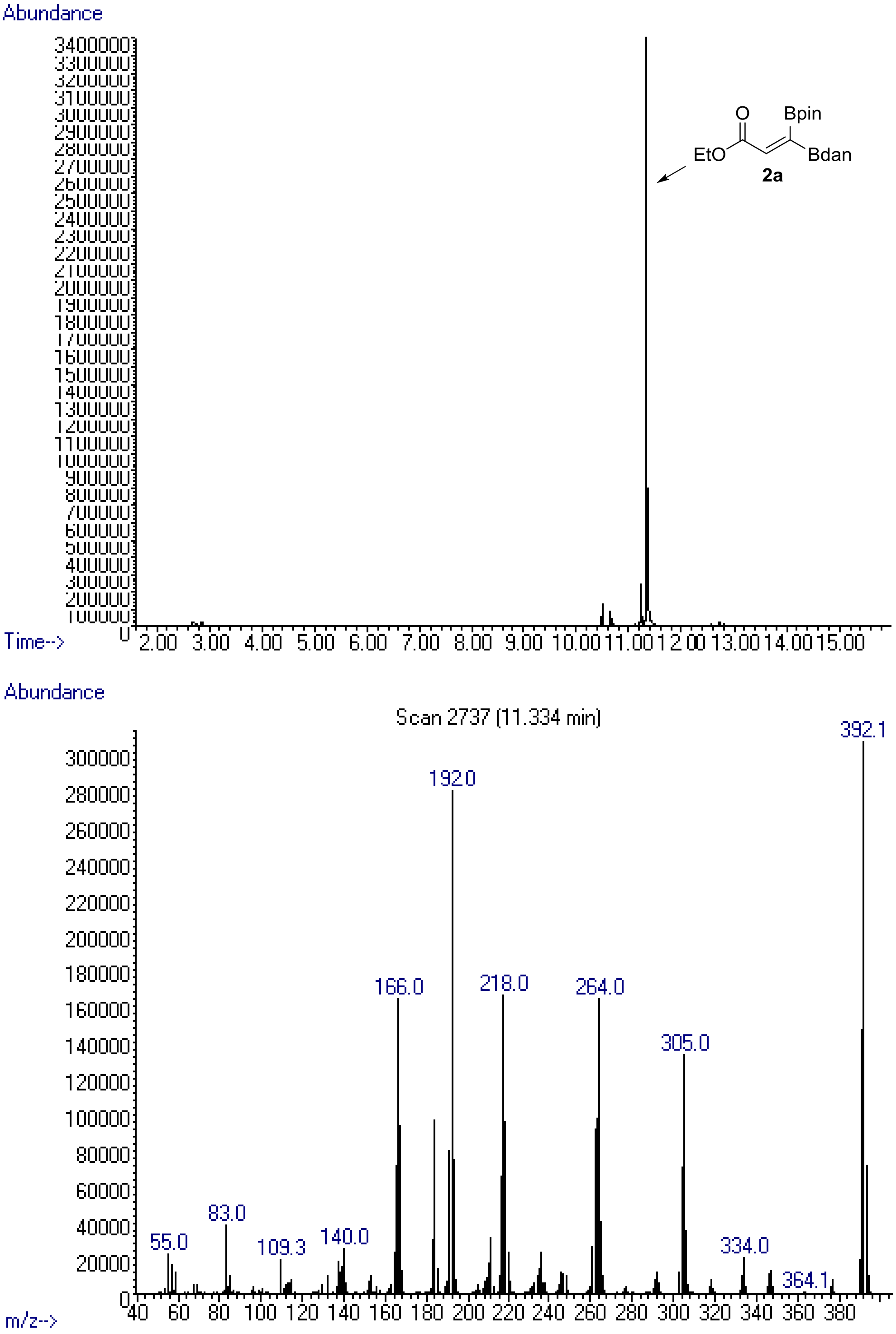

Figure S1. GC-MS of the crude material including the main product **2a**.

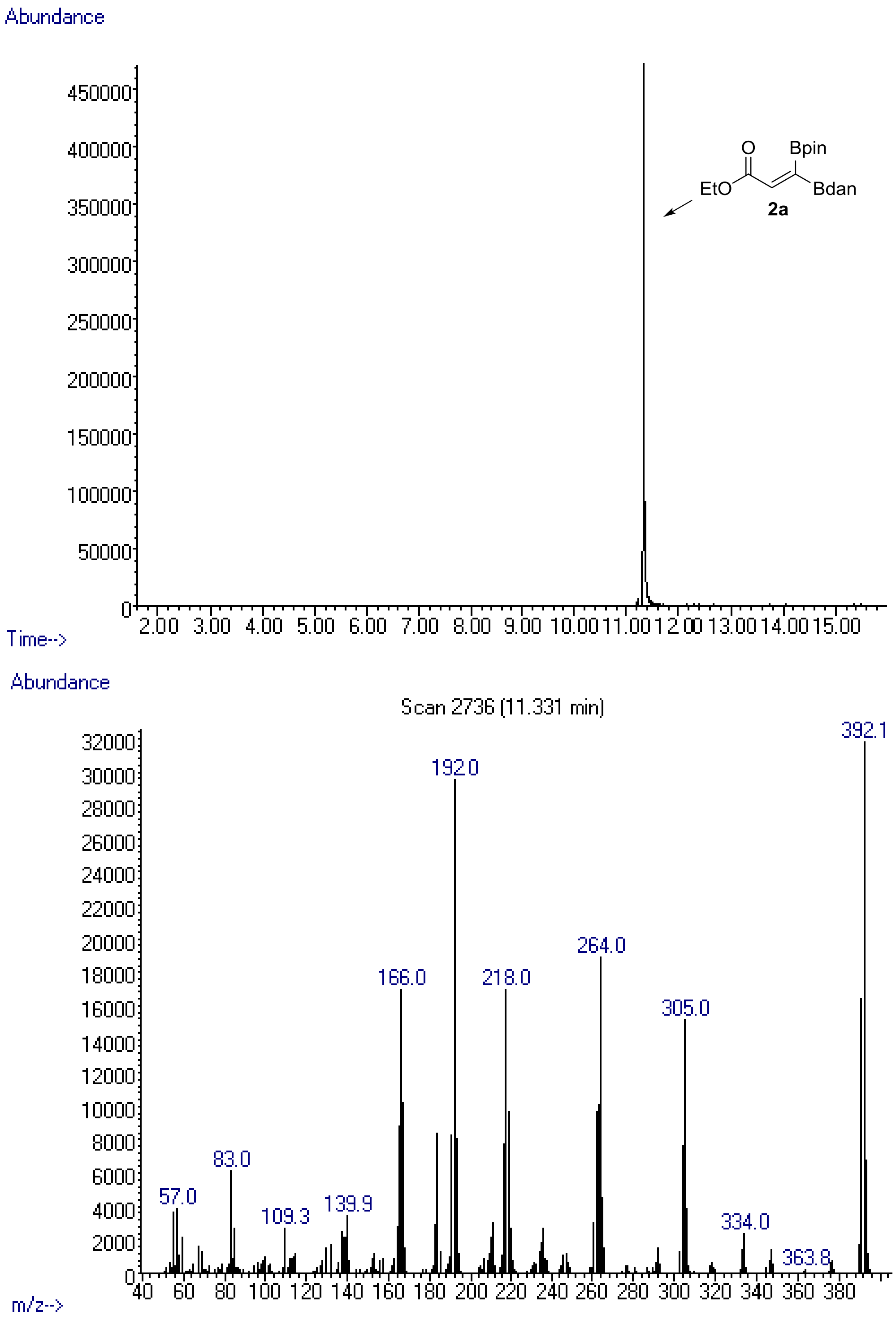

Figure S2. GC-MS of pure compound **2a**.

Characterization of products (2)

Ethyl(*Z*)-3-(1H-naphtho[1,8-de][1,3,2]diazaborinin-2(3H)-yl)-3-(4,4,5,5-tetramethyl-1,3,2-dioxaborolan-2-yl)acrylate (2a)

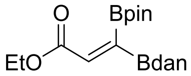

Flash column chromatography (hexane: ethyl acetate = 90:10) afforded the product **2a** (59.6 mg, 76%), yellow solid, m.p.: 174.4 °C.

^{1}H NMR (500 MHz, $CDCl_3$): δ = 7.10 (dd, J = 8, 7 Hz, 2H), 7.03 (dd, J = 8, 1 Hz, 2H), 6.59 (s, 1H), 6.31 (dd, J = 7, 1 Hz, 2H), 5.89 (s, 2H), 4.25 (q, J = 7 Hz, 2H), 1.40 (s, 12H), 1.31 (t, J = 7 Hz, 3H).

^{13}C{^{1}H} NMR (126 MHz, $CDCl_3$): δ = 166.9, 140.6, 136.3, 136.3, 127.5, 120.1, 118.0, 106.1, 84.2, 61.0, 24.9, 14.2.

^{11}B NMR (160 MHz, $CDCl_3$): δ = 31.6, 28.7.

HRMS (ASAP): m/z for $C_{21}H_{27}B_2N_2O_4$ $[M+H]^+$ calcd: 393.2151, found: 393.2142.

Anal. Calcd for $C_{21}H_{26}B_2N_2O_4$: C, 64.33; H, 6.68; N, 7.39; found: C, 64.10; H, 6.78; N, 7.16.

Methyl(*Z*)-3-(1H-naphtho[1,8-de][1,3,2]diazaborinin-2(3H)-yl)-3-(4,4,5,5-tetramethyl-1,3,2-dioxaborolan-2-yl)acrylate (2b)

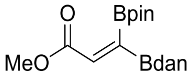

Flash column chromatography (hexane: ethyl acetate = 90:10) afforded the product **2b** (56.7 mg, 75%), yellow solid, m.p.: 177.0 °C.

^{1}H NMR (500 MHz, $CDCl_3$): δ = 7.10 (dd, J = 8, 7 Hz, 2H), 7.03 (dd, J = 8, 1 Hz, 2H), 6.59 (s, 1H), 6.32 (dd, J = 7, 1 Hz, 2H), 5.90 (s, 2H), 3.79 (s, 3H), 1.41 (s, 12H).

^{13}C{^{1}H} NMR (126 MHz, $CDCl_3$): δ = 167.3, 140.6, 136.3, 135.7, 127.5, 120.1, 118.0, 106.1, 84.3, 52.0, 24.9.

^{11}B NMR (160 MHz, $CDCl_3$): δ = 31.8, 28.7.

HRMS (ASAP): m/z for $C_{20}H_{25}B_2N_2O_4$ $[M+H]^+$ calcd: 379.1995, found: 379.1992.

Anal. Calcd for $C_{20}H_{24}B_2N_2O_4$: C, 63.54; H, 6.40; N, 7.41; found: C, 63.33; H, 6.60; N, 7.65.

Tert-butyl(*Z*)-3-(1H-naphtho[1,8-de][1,3,2]diazaborinin-2(3H)-yl)-3-(4,4,5,5-tetramethyl-1,3,2-dioxaborolan-2-yl)acrylate (2c)

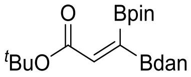

Flash column chromatography (hexane: ethyl acetate = 90:10) afforded the product **2c** (65.6 mg, 78%), yellow solid, m.p.: 181.5 °C.

^{1}H NMR (500 MHz, $CDCl_3$): δ = 7.09 (dd, J = 8, 7 Hz, 2H), 7.02 (dd, J = 8, 1 Hz, 2H), 6.54 (s, 1H), 6.30 (dd, J = 7, 1 Hz, 2H), 5.88 (s, 2H), 1.50 (s, 9H), 1.40 (s, 12H).

$^{13}C\{^1H\}$ NMR (126 MHz, $CDCl_3$): δ = 166.4, 140.7, 138.5, 136.3, 127.5, 120.0, 117.9, 106.0, 84.0, 81.2, 28.1, 24.9.
^{11}B NMR (160 MHz, $CDCl_3$): δ = 32.2, 28.8.
HRMS (ASAP): m/z for $C_{23}H_{31}B_2N_2O_4$ $[M+H]^+$ calcd: 421.2464, found: 421.2473.
Anal. Calcd for $C_{23}H_{30}B_2N_2O_4$: C, 65.76; H, 7.20; N, 6.67; found: C, 65.72; H, 7.39; N, 6.71.

Cyclohexyl(*Z*)-3-(1H-naphtho[1,8-de][1,3,2]diazaborinin-2(3H)-yl)-3-(4,4,5,5-tetramethyl-1,3,2-dioxaborolan-2-yl)acrylate (2d)

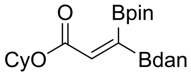

Flash column chromatography (hexane: ethyl acetate = 90:10) afforded the product **2d** (66.0 mg, 74%), yellow solid, m.p.: 244.6 °C.
^1H NMR (500 MHz, $CDCl_3$): δ = 7.13 – 7.06 (m, 2H), 7.02 (dd, *J* = 8, 1 Hz, 2H), 6.59 (s, 1H), 6.31 (dd, *J* = 7, 1 Hz, 2H), 5.90 (s, 2H), 4.88 – 4.83 (m, 1H), 1.94 – 1.82 (m, 2H), 1.79 – 1.69 (m, 2H), 1.60 – 1.17 (m, 18H).
$^{13}C\{^1H\}$ NMR (126 MHz, $CDCl_3$): δ = 166.4, 140.7, 137.0, 136.3, 127.5, 120.1, 118.0, 106.1, 84.2, 73.3, 31.6, 25.4, 24.9, 23.7.
^{11}B NMR (160 MHz, $CDCl_3$): δ = 32.5, 28.7
HRMS (ASAP): m/z for $C_{25}H_{32}B_2N_2O_4$ $[M+H]^+$ calcd: 447.2621, found: 447.2605.
Anal. Calcd for $C_{25}H_{32}B_2N_2O_4$: C, 67.30; H, 7.23; N, 6.28; found: C, 67.72; H, 7.39; N, 6.51.

Benzyl(*Z*)-3-(1H-naphtho[1,8-de][1,3,2]diazaborinin-2(3H)-yl)-3-(4,4,5,5-tetramethyl-1,3,2-dioxaborolan-2-yl)acrylate (2e)

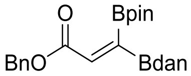

Flash column chromatography (hexane: ethyl acetate = 90:10) afforded the product **2e** (70.8 mg, 78%), yellow solid, m.p.: 221.7 °C.
^1H NMR (500 MHz, $CDCl_3$): δ = 7.51 – 7.30 (m, 5H), 7.10 (dd, *J* = 8, 7 Hz, 2H), 7.03 (dd, *J* = 8, 1 Hz, 2H), 6.65 (s, 1H), 6.31 (dd, *J* = 7, 1 Hz, 2H), 5.90 (s, 2H), 5.23 (s, 2H), 1.41 (s, 12H).
$^{13}C\{^1H\}$ NMR (126 MHz, $CDCl_3$): δ = 166.7, 140.6, 136.3, 135.8, 135.5, 128.5, 128.3, 128.3, 127.5, 120.1, 118.0, 106.1, 84.3, 66.9, 24.9.
^{11}B NMR (160 MHz, $CDCl_3$): δ = 32.4, 28.7.
HRMS (ASAP): m/z for $C_{26}H_{29}B_2N_2O_4$ $[M+H]^+$ calcd: 455.2308, found: 455.2321.
Anal. Calcd for $C_{26}H_{28}B_2N_2O_4$: C, 68.76; H, 6.21; N, 6.17; found: C, 68.73; H, 6.35; N, 6.23.

Furan-2-ylmethyl(*Z*)-3-(1H-naphtho[1,8-de][1,3,2]diazaborinin-2(3H)-yl)-3-(4,4,5,5-tetramethyl-1,3,2-dioxaborolan-2-yl)acrylate (2f)

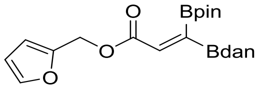

Flash column chromatography (hexane: ethyl acetate = 90:10) afforded the product **2f** (5 h: 38.2 mg, 43%; 10 h: 44.4 mg, 50%), yellow solid, m.p.: 181.0 °C.

^{1}H NMR (500 MHz, $CDCl_3$): δ = 7.43 (dd, *J* = 2, 1 Hz, 1H), 7.09 (dd, *J* = 8, 7 Hz, 2H), 7.02 (dd, *J* = 8, 1 Hz, 2H), 6.60 (s, 1H), 6.42 (dd, *J* = 3, 1 Hz, 1H), 6.37 (dd, *J* = 3, 2 Hz, 1H), 6.30 (dd, *J* = 7, 1 Hz, 2H), 5.88 (s, 2H), 5.17 (s, 2H), 1.41 (s, 12H).

^{13}C{^{1}H} NMR (126 MHz, $CDCl_3$): δ = 166.3, 149.1, 143.3, 140.5, 136.3, 135.6, 127.5, 120.1, 118.1, 110.9, 110.6, 106.1, 84.3, 58.6, 24.9.

^{11}B NMR (160 MHz, $CDCl_3$): δ = 30.9, 28.6

HRMS (ASAP): m/z for $C_{24}H_{26}B_2N_2O_5$ $[M+H]^+$ calcd: 445.2101, found: 445.2087.

Anal. Calcd for $C_{24}H_{26}B_2N_2O_5$: C, 64.91; H, 5.90; N, 6.31; found: C, 64.48; H, 5.93; N, 6.27.

Naphthalen-2-ylmethyl(*Z*)-3-(1H-naphtho[1,8-de][1,3,2]diazaborinin-2(3H)-yl)-3-(4,4,5,5-tetramethyl-1,3,2-dioxaborolan-2-yl)acrylate (2g)

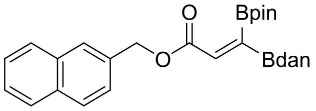

Flash column chromatography (hexane: ethyl acetate = 90:10) afforded the product **2g** (63.5 mg, 63%), white solid, m.p.: 256.6 °C.

^{1}H NMR (500 MHz, $CDCl_3$): δ = 7.86 – 7.84 (m, 4H), 7.57 – 7.39 (m, 3H), 7.10 (dd, *J* = 8, 7 Hz, 2H), 7.03 (dd, *J* = 8, 1 Hz, 2H), 6.68 (s, 1H), 6.31 (dd, *J* = 7, 1 Hz, 2H), 5.91 (s, 2H), 5.39 (s, 2H), 1.41 (s, 12H).

^{13}C{^{1}H} NMR (126 MHz, $CDCl_3$): δ = 166.7, 140.6, 136.3, 135.8, 133.2, 133.1, 133.0, 128.4, 128.0, 127.7, 127.5, 127.4, 126.3, 126.3, 125.8, 120.1, 118.1, 106.2, 84.3, 67.0, 24.9.

^{11}B NMR (160 MHz, $CDCl_3$): δ = 32.0, 28.5

HRMS (ASAP): m/z for $C_{30}H_{30}B_2N_2O_4$ $[M+H]^+$ calcd: 505.2464, found: 505.2452.

Anal. Calcd for $C_{30}H_{30}B_2N_2O_4$: C, 71.47; H, 6.00; N, 5.56; found: C, 70.94; H, 5.98; N, 5.56.

Phenyl(*Z*)-3-(1H-naphtho[1,8-de][1,3,2]diazaborinin-2(3H)-yl)-3-(4,4,5,5-tetramethyl-1,3,2-dioxaborolan-2-yl)acrylate (2h)

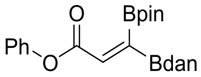

Flash column chromatography (hexane: ethyl acetate = 90:10) afforded the product **2h** (10 h: 57.2 mg, 65%), yellow solid, m.p.: 220.5 °C.

^{1}H NMR (500 MHz, $CDCl_3$): *δ* = 7.43 – 7.34 (m, 2H), 7.27 – 7.21 (m, 1H), 7.18 – 7.09 (m, 4H), 7.05 (dd, *J* = 8, 1 Hz, 2H), 6.80 (s, 1H), 6.34 (dd, *J* = 7, 1 Hz, 2H), 5.96 (s, 2H), 1.35 (s, 12H).

^{13}C{^{1}H} NMR (126 MHz, $CDCl_3$): *δ* = 164.8, 150.6, 140.5, 136.3, 135.5, 129.4, 127.5, 125.9, 121.5, 120.1, 118.2, 106.2, 84.5, 24.9.

^{11}B NMR (160 MHz, $CDCl_3$): *δ* = 31.5, 28.6.

HRMS (ASAP): m/z for $C_{25}H_{26}B_2N_2O_4$ $[M+H]^+$ calcd: 441.2151, found:441.2136.

Anal. Calcd for $C_{25}H_{26}B_2N_2O_4$: C, 68.23; H, 5.95; N, 6.37; found: C, 68.04; H, 5.95; N, 6.43.

Naphthalen-2-yl(*Z*)-3-(1H-naphtho[1,8-de][1,3,2]diazaborinin-2(3H)-yl)-3-(4,4,5,5-tetramethyl-1,3,2-dioxaborolan-2-yl)acrylate (2i)

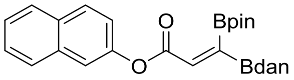

Flash column chromatography (hexane: ethyl acetate = 90:10) afforded the product **2i** (73.5 mg, 75%), yellow solid, m.p.: 222.8 °C.

^{1}H NMR (500 MHz, $CDCl_3$): *δ* = 7.97 – 7.76 (m, 3H), 7.63 (d, *J* = 2 Hz, 1H), 7.52 – 7.46 (m, 2H), 7.30 (dd, *J* = 9, 2 Hz, 1H), 7.12 (dd, *J* = 8, 7 Hz, 2H), 7.06 (dd, *J* = 8, 1 Hz, 2H), 6.86 (s, 1H), 6.36 (dd, *J* = 7, 1 Hz, 2H), 5.99 (s, 2H), 1.35 (s, 12H).

^{13}C{^{1}H} NMR (126 MHz, $CDCl_3$): *δ* = 165.0, 148.2, 140.5, 136.3, 135.4, 133.7, 131.5, 129.4, 127.7, 127.7, 127.5, 126.5, 125.7, 121.1, 120.1, 118.6, 118.2, 106.2, 84.5, 24.9.

^{11}B NMR (160 MHz, $CDCl_3$): *δ* = 31.7, 28.7.

HRMS (ASAP): m/z for $C_{29}H_{28}B_2N_2O_4$ $[M+H]^+$ calcd: 491.2308, found:491.2311

Anal. Calcd for $C_{29}H_{28}B_2N_2O_4$: C, 71.06; H, 5.76; N, 5.72; found: C, 70.54; H, 5.81; N, 5.74.

Hept-2-yn-1-yl(*Z*)-3-(1H-naphtho[1,8-de][1,3,2]diazaborinin-2(3H)-yl)-3-(4,4,5,5-tetramethyl-1,3,2-dioxaborolan-2-yl)acrylate (2j)

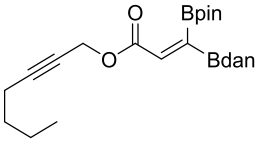

Flash column chromatography (hexane: ethyl acetate = 90:10) afforded the product **2j** (56.8 mg, 62%), yellow solid, m.p.: 152.1 °C.

^{1}H NMR (500 MHz, $CDCl_3$): *δ* = 7.10 (dd, *J* = 8, 7 Hz, 2H), 7.03 (dd, *J* = 8, 1 Hz, 2H), 6.62 (s,

1H), 6.31 (dd, J = 7, 1 Hz, 2H), 5.89 (s, 2H), 4.78 (s, 2H), 2.30 – 2.14 (m, 2H), 1.57 – 1.46 (m, 2H), 1.45 – 1.37 (m, 14H), 0.92 (t, J = 7 Hz, 3H).
^{13}C{^{1}H} NMR (126 MHz, $CDCl_3$): δ = 166.1, 140.5, 136.3, 135.5, 127.5, 120.1, 118.1, 106.2, 88.1, 84.3, 73.6, 53.4, 30.4, 24.9, 21.9, 18.4, 13.6.
^{11}B NMR (160 MHz, $CDCl_3$): δ = 32.2, 28.5.
HRMS (ASAP): m/z for $C_{26}H_{32}B_2N_2O_4$ $[M+H]^+$ calcd: 459.2621, found:459.2606.
Anal. Calcd for $C_{26}H_{32}B_2N_2O_4$: C, 68.16; H, 7.04; N, 6.11; found: C, 68.16; H, 7.08; N, 6.18.

3-Methylbut-2-en-1-yl(*Z*)-3-(1H-naphtho[1,8-de][1,3,2]diazaborinin-2(3H)-yl)-3-(4,4,5,5-tetramethyl-1,3,2-dioxaborolan-2-yl)acrylate (2k)

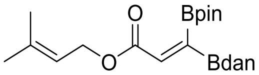

Flash column chromatography (hexane: ethyl acetate = 90:10) afforded the product **2k** (62.2 mg, 72%), yellow solid, m.p.: 155.1 °C.
^{1}H NMR (500 MHz, $CDCl_3$): δ = 7.09 (dd, J = 8, 7 Hz, 2H), 7.02 (dd, J = 8, 1 Hz, 2H), 6.59 (s, 1H), 6.30 (dd, J = 7, 1 Hz, 2H), 5.88 (s, 2H), 5.39-5.36 (m, 1H), 4.68 (d, J = 7 Hz, 2H), 1.77 (s, 3H), 1.72 (s, 3H), 1.41 (s, 12H).
^{13}C{^{1}H} NMR (126 MHz, $CDCl_3$): δ = 166.9, 140.6, 139.5, 136.3, 136.2, 127.5, 120.1, 118.3, 118.0, 106.1, 84.2, 62.0, 25.8, 24.9, 18.0.
^{11}B NMR (160 MHz, $CDCl_3$): δ = 32.5, 29.1.
HRMS (ASAP): m/z for $C_{24}H_{30}B_2N_2O_4$ $[M+H]^+$ calcd: 433.2464, found:433.2447.
Anal. Calcd for $C_{24}H_{30}B_2N_2O_4$: C, 66.71; H, 7.00; N, 6.48; found: C, 66.84; H, 7.21; N, 6.39.

Allyl(*Z*)-3-(1H-naphtho[1,8-de][1,3,2]diazaborinin-2(3H)-yl)-3-(4,4,5,5-tetramethyl-1,3,2-dioxaborolan-2-yl)acrylate (2l)

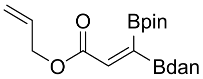

Flash column chromatography (hexane: ethyl acetate = 90:10) afforded the product **2l** (60.6 mg, 75%), yellow solid, m.p.: 181.5 °C.
^{1}H NMR (500 MHz, $CDCl_3$): δ = 7.10 (dd, J = 8, 7 Hz, 2H), 7.03 (dd, J = 8, 1 Hz, 2H), 6.62 (s, 1H), 6.32 (dd, J = 7, 1 Hz, 2H), 6.06 – 5.86 (m, 3H), 5.37-5.33 (m, 1H), 5.28-5.25 (m, 1H), 4.69 (ddd, J = 6, 1, 1 Hz, 2H), 1.41 (s, 12H).
^{13}C{^{1}H} NMR (126 MHz, $CDCl_3$): δ = 166.5, 140.6, 136.3, 135.8, 131.9, 127.5, 120.1, 118.5, 118.0, 106.1, 84.3, 65.7, 24.9.
^{11}B NMR (160 MHz, $CDCl_3$): δ = 32.2, 28.9.
HRMS (ASAP): m/z for $C_{22}H_{26}B_2N_2O_4$ $[M+H]^+$ calcd: 405.2151, found:405.2137.
Anal. Calcd for $C_{22}H_{26}B_2N_2O_4$: C, 65.39; H, 6.49; N, 6.93; found: C, 65.22; H, 6.59; N, 6.98.

(*Z*)-N-methyl-3-(1H-naphtho[1,8-de][1,3,2]diazaborinin-2(3H)-yl)-N-phenyl-3-(4,4,5,5-tetramethyl-1,3,2-dioxaborolan-2-yl)acrylamide (2m)

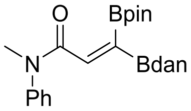

Flash column chromatography (hexane: ethyl acetate = 85:15) afforded the product **2m** (5 h: 57.1 mg, 63%; 10 h: 78.8 mg, 87%), yellow solid, m.p.: 256.6 °C.

^{1}H NMR (500 MHz, $CDCl_3$): *δ* = 7.42 – 7.35 (m, 3H), 7.10 (dd, *J* = 8, 7 Hz, 2H), 7.01 – 6.93 (m, 4H), 6.53 (s, 2H), 6.35 (dd, *J* = 7, 1 Hz, 2H), 6.24 (s, 1H), 3.25 (s, 3H), 1.31 (s, 12H).

^{13}C{^{1}H} NMR (126 MHz, $CDCl_3$): *δ* = 172.5, 141.4, 140.7, 136.4, 130.0, 129.0, 128.9, 127.5, 126.4, 120.2, 117.1, 105.7, 80.9, 39.2, 25.7.

^{11}B NMR (160 MHz, $CDCl_3$): *δ* = 29.2, 17.2.

HRMS (ASAP): m/z for $C_{26}H_{29}B_2N_3O_3$ $[M+H]^+$ calcd: 454.2468, found:454.2456.

Anal. Calcd for $C_{26}H_{29}B_2N_3O_3$: C, 68.91; H, 6.45; N, 9.27; found: C, 68.88; H, 6.51; N, 9.25.

(*Z*)-3-(1H-naphtho[1,8-de][1,3,2]diazaborinin-2(3H)-yl)-1-(piperidin-1-yl)-3-(4,4,5,5-tetramethyl-1,3,2-dioxaborolan-2-yl)prop-2-en-1-one (2n)

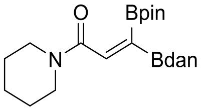

Flash column chromatography (hexane: ethyl acetate = 85:15) afforded the product **2n** (5 h: 25.0 mg, 29%; 10 h: 43.1 mg, 50%), yellow solid, m.p.: 154.0 °C.

^{1}H NMR (500 MHz, $CDCl_3$): *δ* = 7.11 – 7.03 (m, 2H), 6.97 (dd, *J* = 8, 1 Hz, 2H), 6.78 (s, 1H), 6.41 (s, 2H), 6.32 (dd, *J* = 7, 1 Hz, 2H), 3.58 – 3.53 (m, 4H), 1.67 – 1.47 (m, 6H), 1.32 (s, 12H).

^{13}C{^{1}H} NMR (126 MHz, $CDCl_3$): *δ* = 171.0, 141.4, 136.4, 127.6, 126.5, 120.2, 117.2, 105.7, 80.6, 46.9, 46.3, 26.1, 25.7, 25.0, 23.7.

^{11}B NMR (160 MHz, $CDCl_3$): *δ* = 29.9, 15.4.

HRMS (ASAP): m/z for $C_{24}H_{31}B_2N_3O_3$ $[M+H]^+$ calcd: 432.2624, found:432.2610.

Anal. Calcd for $C_{24}H_{31}B_2N_3O_3$: C, 66.86; H, 7.25; N, 9.75; found: C, 66.39; H, 7.28; N, 9.65.

V. Investigations Concerning the Reaction Mechanism

Sequential stoichiometric reaction

A mixture of **1a** (19.6 mg, 0.2 mmol) in THF (1 mL) was cooled to -78 °C. Then ^{n}BuLi (80 μL, 2.5 M in hexane, 0.2 mmol) was added dropwise at -78 °C. After stirring for 30 min, BpinBdan (58.8 mg, 0.2 mmol) in THF (1 mL) was added dropwise at –78 °C. Then, the mixture was warmed to ambient temperature with stirring for 1 h. Subsequently, ^{t}BuOH (19 μL, 0.2 mmol) was added and the mixture was stirred for another 1 h. The solvent was removed under reduced pressure to give a brown oil. The yield of **2a** was determined to be 23% by ^{1}H NMR analysis using 1,3,5-trioxacyclohexane (0.09 mmol) as an internal standard (Figure S3). This stoichiometric reaction is consistent with the formation of intermediate acetylide **A** via deprotonation of the terminal alkyne.

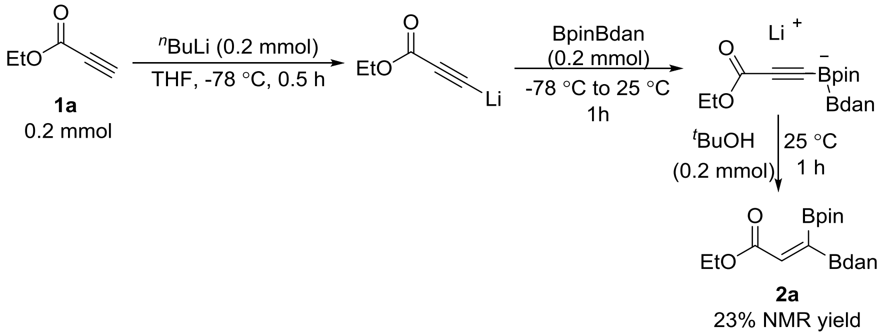

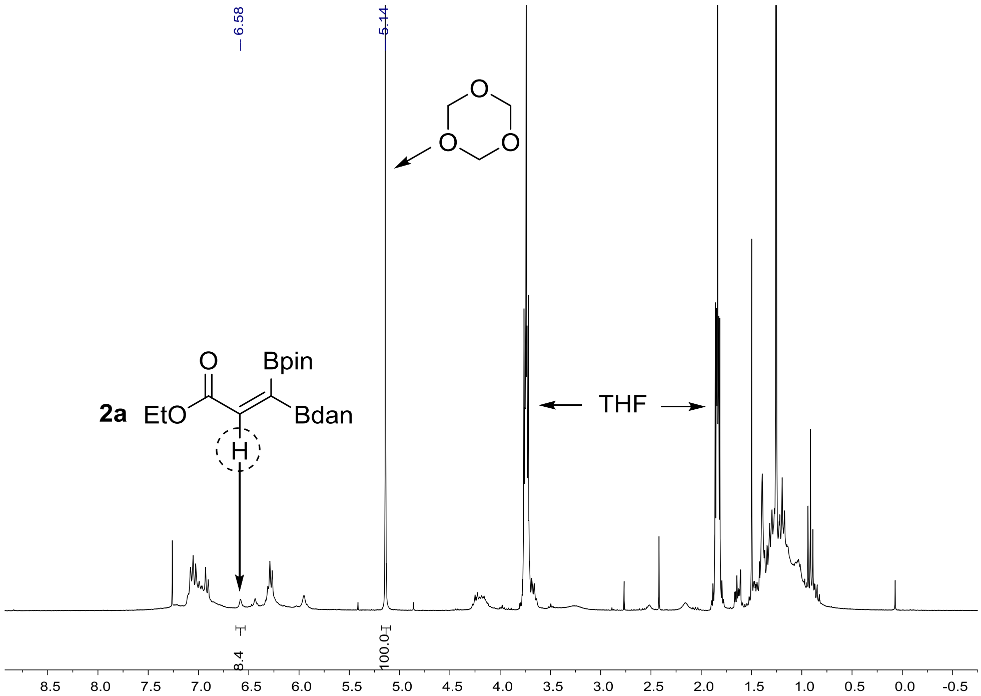

Figure S3. ^{1}H NMR spectrum of the crude material including **2a** (300 MHz, $CDCl_3$); 1,3,5-trioxacyclohexane (0.09 mmol) was used as an internal standard.

Deuterium labelling experiment

Using deuterated ethyl propiolate **1a-*d*** as the substrate (deuterium content = 90%; see Figure S4) under the standard reaction conditions, gave product **2a-*d*** with 50% D at the alkenyl position and 45% D at the N-H of Bdan (Figures S5 and S6). Thus, H/D exchange between ^{t}BuOD and the N-H of Bdan makes it impossible to determine whether the proton on the alkene comes from N-H of Bdan or from ^{t}BuOH directly.

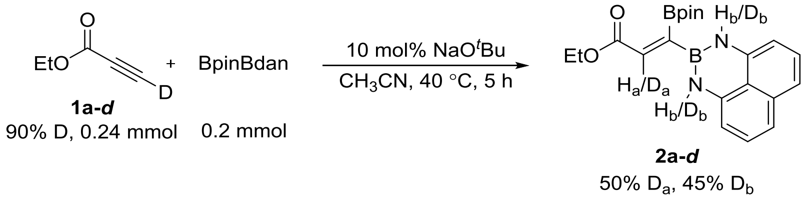

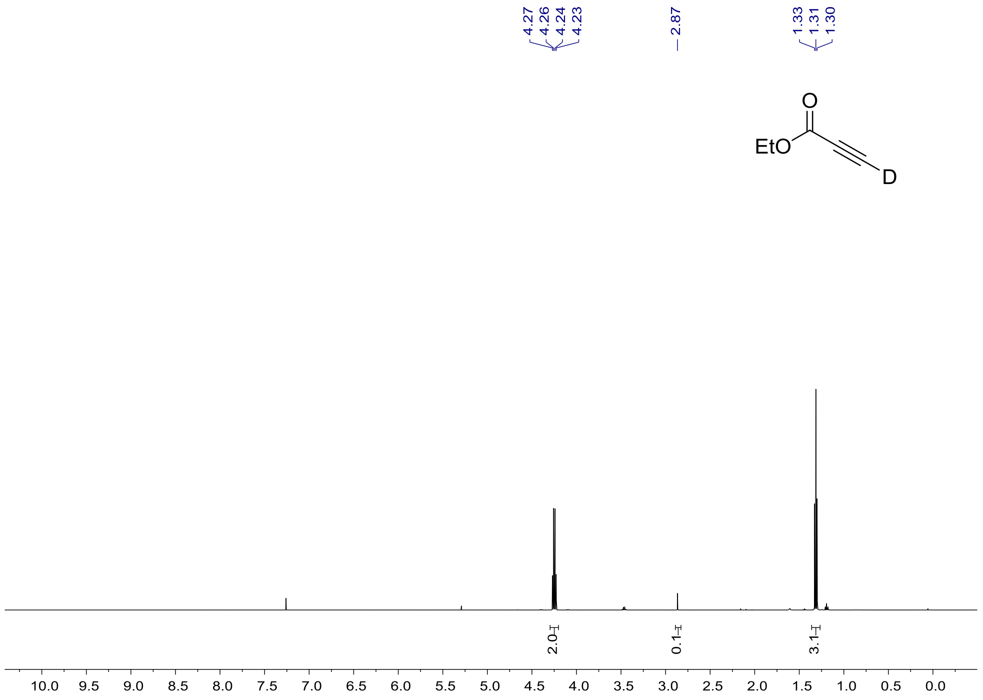

Figure S4. ^{1}H NMR spectrum of **1a-*d***. ^{1}H NMR (500 MHz, $CDCl_3$) δ = 4.25 (q, J = 7 Hz, 2H), 2.87 (s, 0.1H), 1.31 (t, J = 7 Hz, 3H).

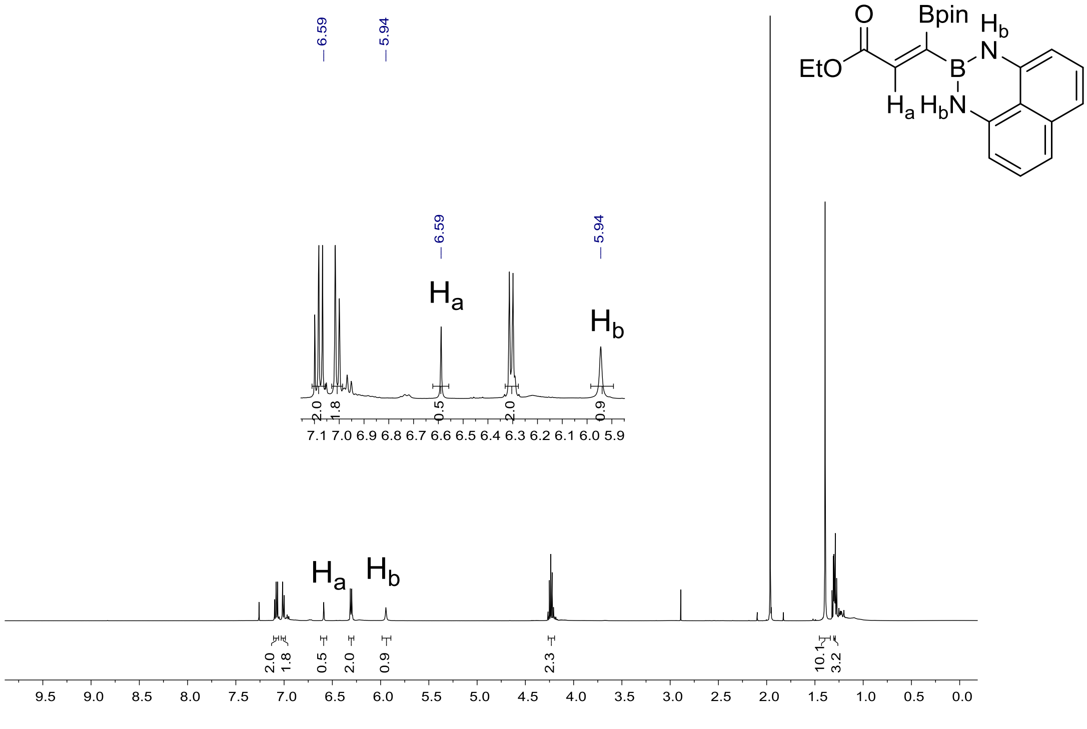

Figure S5. ^{1}H NMR spectrum (500 MHz, $CDCl_3$) of **2a-*d*** (reaction mixture).

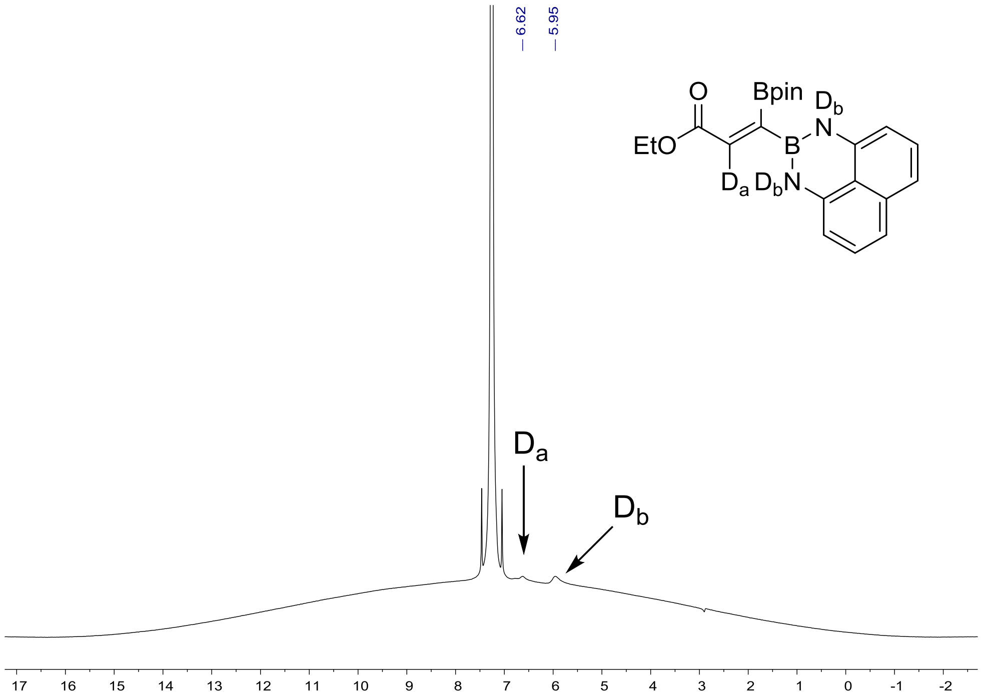

Figure S6. ^{2}H NMR spectrum (500 MHz, $CDCl_3$) of **2a-*d*** (reaction mixture).

Computational methods

DFT calculations were carried out with the Gaussian09 package.[6] Geometry optimization was performed with the B3LYP-D3 functional[7] and 6-31+G(d) basis set in MeCN solvent (using the SMD [8] solvent model). Frequency analysis was carried out at the same level to verify the stationary points as an intermediate or transition state and to obtain the thermodynamic energy corrections assuming a standard state of 1 atm and 298.15 K. Intrinsic reaction coordinates (IRC) [9] were calculated to confirm the connection between the transition state and the correct reactant/product. Single-point calculations were carried out with the M11 functional [10] and 6-311+G(d,p) basis set in MeCN solvent (using the SMD solvent model).

Due to the different migration directions relative to the carboxyl group in intermediates **5** and **9**, there are four paths to realize 1,2-migration of Bpin or Bdan moiety and generate allenylic axial chiral isomers **7** and **21**. As shown in Figure 2 and Figure S7, the two paths to form **21** via transition states **19-ts** and **20-ts** have a slightly higher barrier than those to form **7**. In the following hydrogen transfer step, the relative free energies of transition states **22-ts** and **23-ts** are very close to their isomers **11-ts** and **13-ts** given in Figure 2.

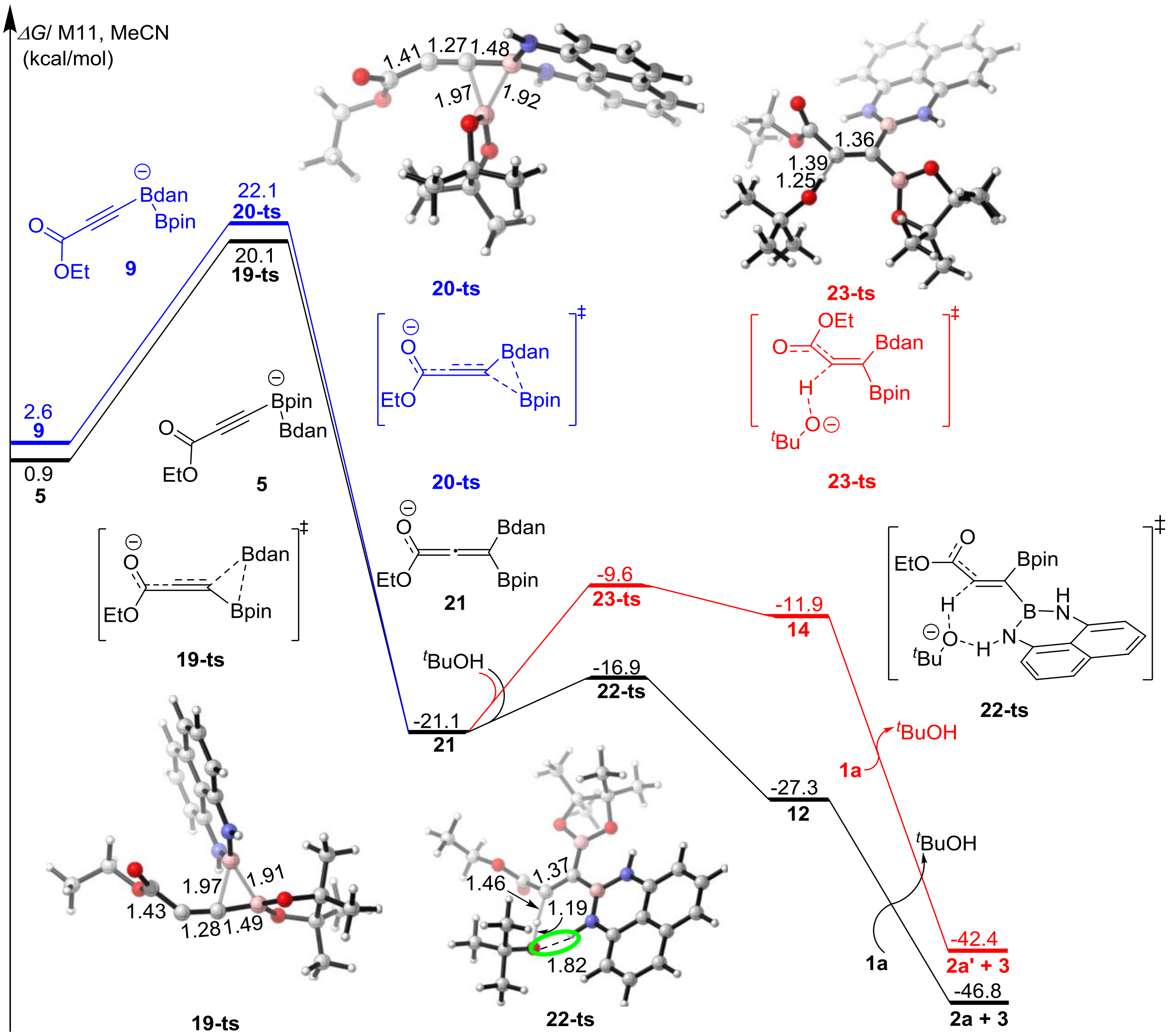

Figure S7. The other two pathways for the 1,2-migration step to form allenylic intermediate **21**.

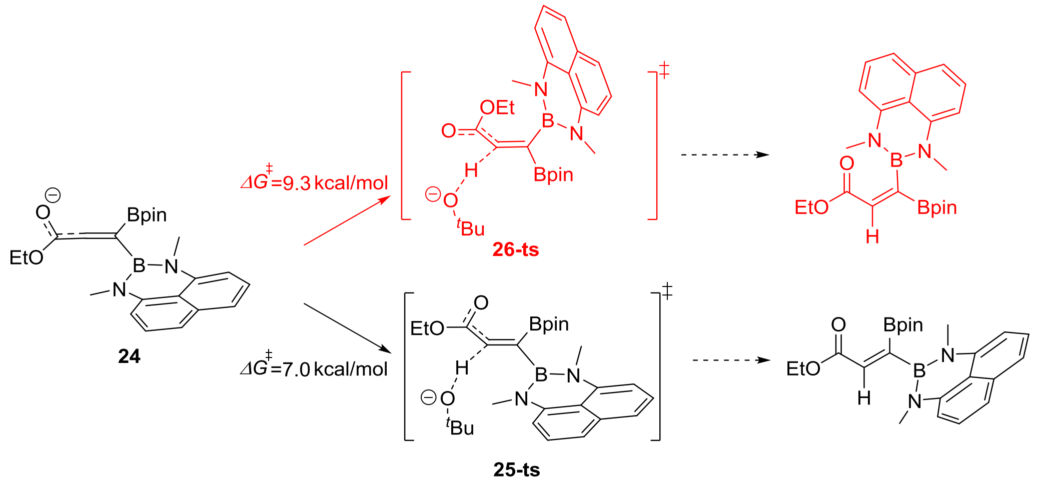

Figure S8. The activation free energies ($\Delta G^{\neq}$) of protonation transition states **25-ts** and **26-ts** with NH replaced by NMe in the Bdan group, calculated at the M11/(6-311+G(d, p), SMD)//B3LYP-D3/(6-31+G(d) ,SMD) level.

In order to examine the effect of the NH interaction with ^{t}BuOH in the protonation step, the hydrogen of NH was replaced by methyl; thus a proton from ^{t}BuOH would be transferred to allenolate **24** without the NH interaction. As shown in Figure S8, **25-ts** has a lower free energy than **26-ts** by 2.3 kcal/mol, which indicates that the stereoselectivity is unchanged and the main product is the (Z)-diborylalkene isomer. Compared with Figure 2 and Figure S7, the energy barrier becomes higher by about 3.0 kcal/mol for the path to form the (Z)-diborylalkene product without the NH interaction with ^{t}BuOH. In addition, the gap between the barriers of the two paths to **2a** and **2a'** becomes smaller. These results demonstrate that the NH interaction with ^{t}BuOH can promote the generation of (Z)-diborylalkene products, although it may not the stereoselectivity controlling factor.

Table S4: Thermal correction of Gibbs free energy (TCG, hartree), total electronic energy $E_{(elec\text{-}B3LYP\text{-}D3)}$ at the B3LYP-D3/6-31+G(d) level in MeCN, total electronic energy $E_{(elec\text{-}M11\text{-}MeCN)}$ at the M11/6-311+G(d,p) level in MeCN, and the B3LYP-D3 calculated imaginary frequencies for the transition states (IF).

Compounds	TCG	$E_{(elec\text{-}B3LYP\text{-}D3)}$	$E_{(elec\text{-}M11\text{-}MeCN)}$	IF
**1a**	0.067239	-344.552855	-344.449678	
**2a**	0.39447	-1276.418952	-1275.914057	
**2a′**	0.395832	-1276.415869	-1275.908338	
**^{t}BuO$^-$**	0.093025	-233.187008	-233.091116	
**^{t}BuOH**	0.106429	-233.707109	-233.61377	
**BpinBdan**	0.300975	-931.759865	-931.363488	
**3**	0.055484	-344.062121	-343.964192	
**4-ts**	0.376921	-1275.823646	-1275.3277	-158.09
**5**	0.375395	-1275.839073	-1275.345131	
**6-ts**	0.379028	-1275.822548	-1275.320184	-421.12
**7**	0.38098	-1275.88506	-1275.382682	
**8-ts**	0.376145	-1275.822161	-1275.326022	-193.37
**9**	0.374809	-1275.838201	-1275.34186	
**10-ts**	0.37694	-1275.818832	-1275.313498	-462.95
**11-ts**	0.50481	-1509.608835	-1509.010149	-1220.68
**12**	0.506144	-1509.62823	-1509.028199	
**13-ts**	0.503347	-1509.596228	-1508.996653	-1475.6
**14**	0.506108	-1275.856058	-1509.003681	
**15-ts**	0.374959	-1275.652392	-1275.355929	-1757.8
**16**	0.381419	-1275.909563	-1275.406993	
**17-ts**	0.504242	-1509.626116	-1509.027199	-997.9
**18-ts**	0.501275	-1509.578297	-1508.980996	-1712.62
**19-ts**	0.37864	-1275.820885	-1275.317821	-409.67
**20-ts**	0.377622	-1275.818997	-1275.313597	-477.96
**21**	0.380779	-1275.884969	-1275.385697	
**22-ts**	0.504261	-1509.609093	-1509.009806	-1221.55
**23-ts**	0.5026	-1509.596871	-1508.996473	-1471.55
**24**	0.432714	-1354.497338	-1353.945759	
**25-ts**	0.555995	-1588.216947	-1587.56517	-1448.05
**26-ts**	0.554733	-1588.211759	-1587.560284	-1427.57

VI. Synthetic Applications of the Mixed 1,1-Diborylalkene

General procedures for products 28

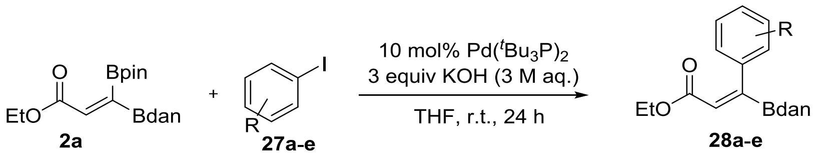

In a glove box, a tube (20 mL) containing Pd(^{t}Bu$_3$P)$_2$ (5.1 mg, 0.01 mol), **2a** (39.2 mg, 0.1 mmol), aryl iodides **27** (1.1 equiv) and dry THF (1 mL) was capped with a septum and then removed from the glove box. Degassed aqueous KOH (100 μL, 3 M, 0.3 mmol) was added via syringe, and the mixture was stirred at room temperature for 24 h. Then, the mixture was filtered through a pad of Celite and washed through with Et_2O (20 mL). The filtrate was concentrated under vacuum, and the residue was purified by flash column chromatography to yield a yellow liquid.

NOE study of compound 28a

A ^{1}H-^{1}H NOESY study of compound **28a** shows correlations between H_a and H_b, H_a and H_c, as well as H_a and H_d. The NOESY spectrum shows a strong correlation signal between the olefinic proton H_b and the NH proton (H_a), but not the protons of the phenyl group (H_d) (Figure S8).

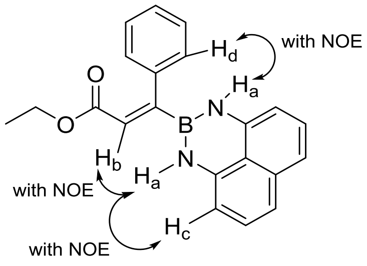

28a

H$_a$ 5.66 ppm
H$_b$ 6.42 ppm
H$_c$ 6.27 ppm
H$_d$ 7.18 ppm

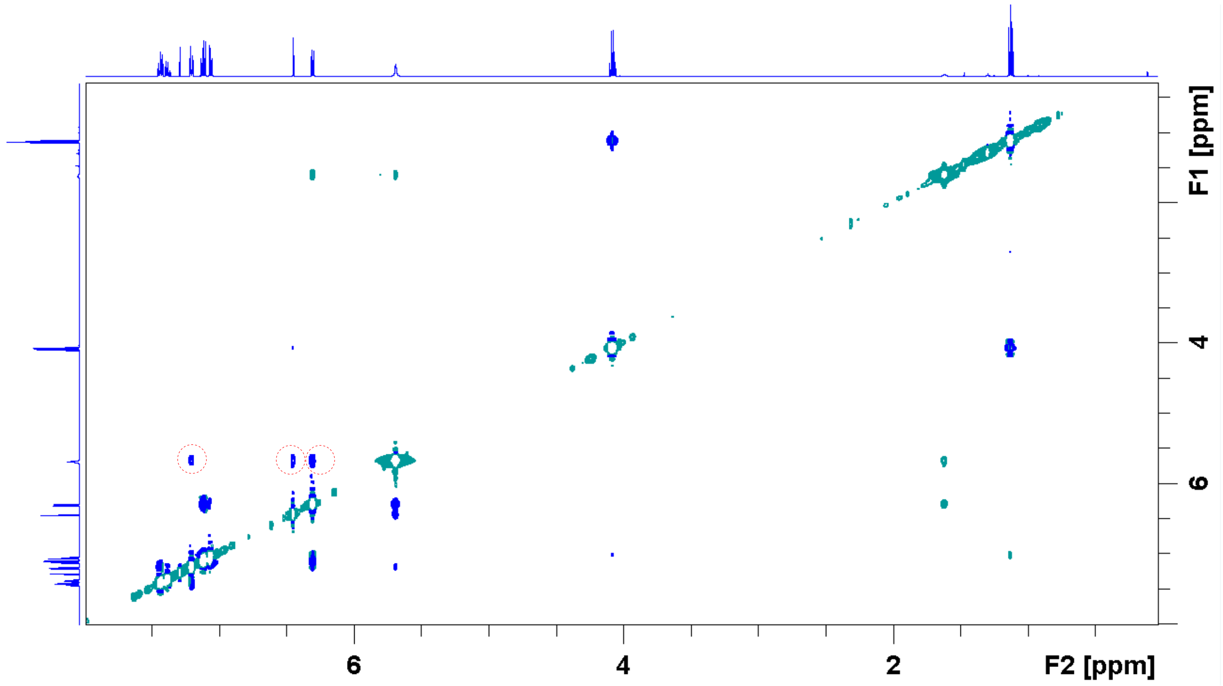

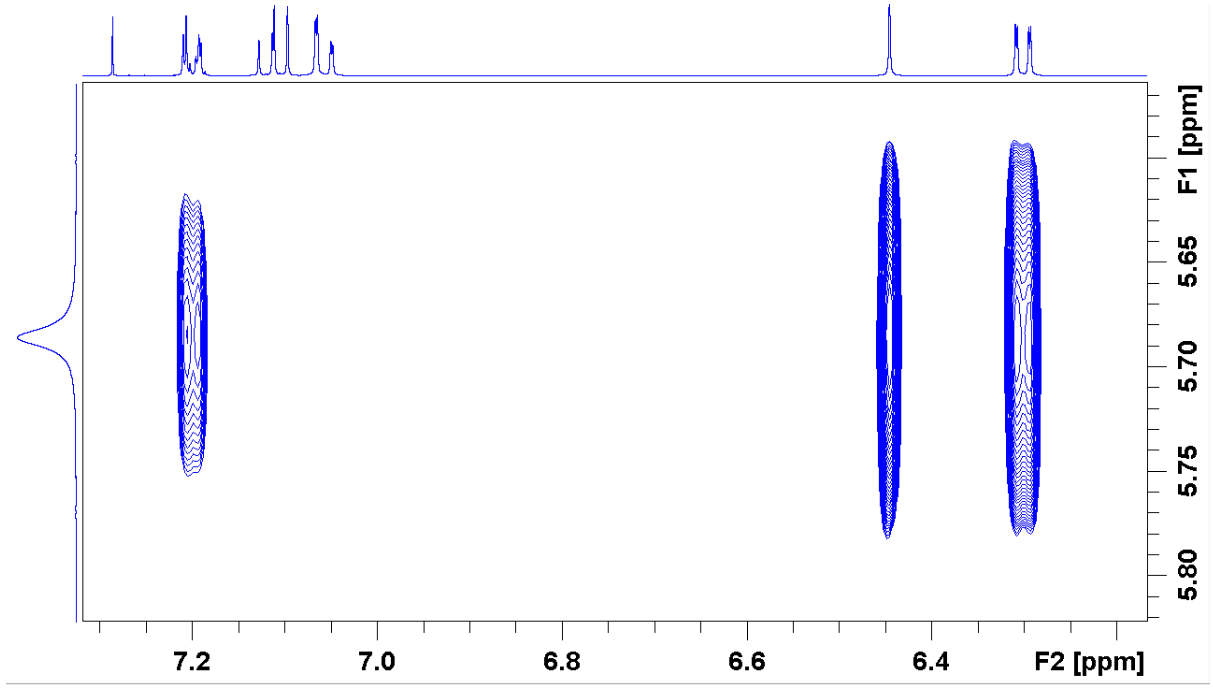

Figure S8. A 2D NOESY spectrum of compound **28a**.

Characterization of products (28)

Ethyl (*Z*)-3-(1H-naphtho[1,8-de][1,3,2]diazaborinin-2(3H)-yl)-3-phenylacrylate (28a)

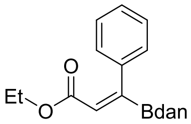

Flash column chromatography (hexane: ethyl acetate = 90:10) afforded the product **28a** (41.7 mg, 61%), yellow liquid.

^{1}H NMR (300 MHz, $CDCl_3$): δ = 7.45 – 7.31 (m, 3H), 7.21 – 7.14 (m, 2H), 7.13 – 7.00 (m, 4H), 6.42 (s, 1H), 6.28 (dd, *J* = 7, 1 Hz, 2H), 5.66 (s, 2H), 4.05 (q, *J* = 7 Hz, 2H), 1.10 (t, *J* = 7 Hz, 3H).

^{13}C{^{1}H} NMR (75 MHz, $CDCl_3$): δ = 165.6, 140.4, 139.3, 136.2, 128.3, 127.5, 127.4, 127.2, 126.8, 120.0, 118.2, 106.3, 60.3, 13.9.

^{11}B NMR (96 MHz, $CDCl_3$): δ = 28.0.

HRMS (ASAP): m/z for $C_{21}H_{19}B_1N_2O_2$ $[M+H]^+$ calcd: 343.1612, found:343.1598.

Anal. Calcd for $C_{21}H_{19}B_1N_2O_2$: C, 73.71; H, 5.60; N, 8.19; found: C, 73.35; H, 5.57; N, 8.10.

Ethyl (*Z*)-3-(4-methoxyphenyl)-3-(1H-naphtho[1,8-de][1,3,2]diazaborinin-2(3H)-yl)acrylate (28b)

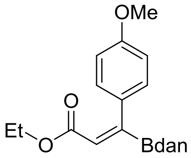

Flash column chromatography (hexane: ethyl acetate = 90:10) afforded the product **28b** (48.4 mg, 65%), yellow liquid.

^{1}H NMR (300 MHz, $CDCl_3$): δ = 7.20 – 7.00 (m, 6H), 6.96 – 6.90 (m, 2H), 6.36 (s, 1H), 6.28 (dd, *J* = 7, 1 Hz, 2H), 5.67 (s, 2H), 4.09 (q, *J* = 7 Hz, 2H), 3.85 (s, 3H), 1.16 (t, *J* = 7 Hz, 3H).

^{13}C{^{1}H} NMR (75 MHz, $CDCl_3$): δ = 165.8, 159.2, 140.5, 136.3, 131.1, 129.1, 127.5, 125.9, 120.0, 118.1, 113.7, 106.2, 60.2, 55.2, 14.0.

^{11}B NMR (96 MHz, $CDCl_3$): δ = 27.8.

HRMS (ASAP): m/z for $C_{22}H_{21}B_1N_2O_3$ $[M+H]^+$ calcd: 373.1718, found: 373.1704.

Anal. Calcd for $C_{22}H_{21}B_1N_2O_3$: C, 70.99; H, 5.69; N, 7.53; found: C, 71.48; H, 5.77; N, 7.68.

Ethyl (*Z*)-3-(1H-naphtho[1,8-de][1,3,2]diazaborinin-2(3H)-yl)-3-(p-tolyl)acrylate (28c)

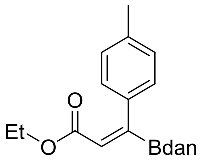

Flash column chromatography (hexane: ethyl acetate = 90:10) afforded the product **28c** (45.6 mg, 64%), yellow liquid.

^{1}H NMR (300 MHz, $CDCl_3$): δ = 7.22 – 7.19 (m, 2H), 7.12 – 7.00 (m, 6H), 6.38 (s, 1H), 6.27 (dd, *J* = 7, 1 Hz, 2H), 5.67 (s, 2H), 4.08 (q, *J* = 7 Hz, 2H), 2.40 (s, 3H), 1.14 (t, *J* = 7 Hz, 3H).

^{13}C{^{1}H} NMR (75 MHz, $CDCl_3$): δ = 165.7, 140.5, 137.2, 136.2, 136.1, 129.0, 127.5, 127.3, 126.3, 120.0, 118.1, 106.2, 60.2, 21.3, 14.0.

^{11}B NMR (96 MHz, $CDCl_3$): δ = 27.5.

HRMS (ASAP): m/z for $C_{22}H_{21}B_1N_2O_2$ $[M+H]^+$ calcd: 357.1769, found: 357.1760.

Anal. Calcd for $C_{22}H_{21}B_1N_2O_2$: C, 74.18; H, 5.94; N, 7.86; found: C, 73.85; H, 5.77; N, 7.74.

Ethyl (*Z*)-3-(1H-naphtho[1,8-de][1,3,2]diazaborinin-2(3H)-yl)-3-(m-tolyl)acrylate (28d)

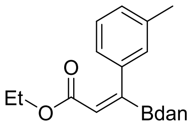

Flash column chromatography (hexane: ethyl acetate = 90:10) afforded the product **28d** (34.9 mg, 49%), yellow liquid.

^{1}H NMR (300 MHz, $CDCl_3$): δ = 7.30 (d, *J* = 8 Hz, 1H), 7.15 (d, *J* = 8 Hz, 1H), 7.15 – 6.98 (m, 4H), 7.02 – 6.92 (m, 2H), 6.39 (s, 1H), 6.28 (dd, *J* = 7, 1 Hz, 2H), 5.66 (s, 2H), 4.05 (q, *J* = 7 Hz, 2H), 2.38 (s, 3H), 1.10 (t, *J* = 7 Hz, 3H).

^{13}C{^{1}H} NMR (75 MHz, $CDCl_3$): δ = 165.7, 140.5, 139.2, 137.8, 136.3, 128.2, 127.8, 127.5, 126.6, 124.3, 120.0, 118.2, 106.2, 60.2, 21.5, 13.9.

^{11}B NMR (96 MHz, $CDCl_3$): δ = 28.3.

HRMS (ASAP): m/z for $C_{22}H_{21}B_1N_2O_2$ $[M+H]^+$ calcd: 357.1769, found: 357.1754.

Anal. Calcd for $C_{22}H_{21}B_1N_2O_2$: C, 74.18; H, 5.94; N, 7.86; found: C, 74.55; H, 6.07; N, 8.05.

Ethyl (*Z*)-3-(3,5-dimethylphenyl)-3-(1H-naphtho[1,8-de][1,3,2]diazaborinin-2(3H)-yl)acrylate (28e)

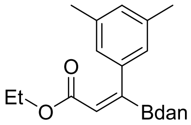

Flash column chromatography (hexane: ethyl acetate = 90:10) afforded the product **28e** (40.7 mg, 55%), yellow liquid.

^{1}H NMR (300 MHz, $CDCl_3$): δ = 7.15 – 6.99 (m, 4H), 6.97 (br s, 1H), 6.77 (br s, 2H), 6.38 (s, 1H), 6.28 (dd, J = 7, 1 Hz, 2H), 5.67 (s, 2H), 4.06 (q, J = 7 Hz, 2H), 2.34 (s, 6H), 1.11 (t, J = 7 Hz, 3H).
^{13}C{^{1}H} NMR (75 MHz, $CDCl_3$): δ = 165.8, 140.5, 139.2, 137.7, 136.3, 129.1, 127.5, 126.5, 124.9, 120.0, 118.1, 106.2, 60.2, 21.4, 13.9.
^{11}B NMR (96 MHz, $CDCl_3$): δ = 27.8.
HRMS (ASAP): m/z for $C_{23}H_{24}B_1N_2O_2$ $[M+H]^+$ calcd: 371.1925, found: 371.1913.
Anal. Calcd for $C_{23}H_{24}B_1N_2O_2$: C, 74.61; H, 6.26; N, 7.57; found: C, 74.48; H, 5.97; N, 7.45.

VII. NMR Spectra

^{1}H NMR spectrum (300 MHz, $CDCl_3$) of **1f**

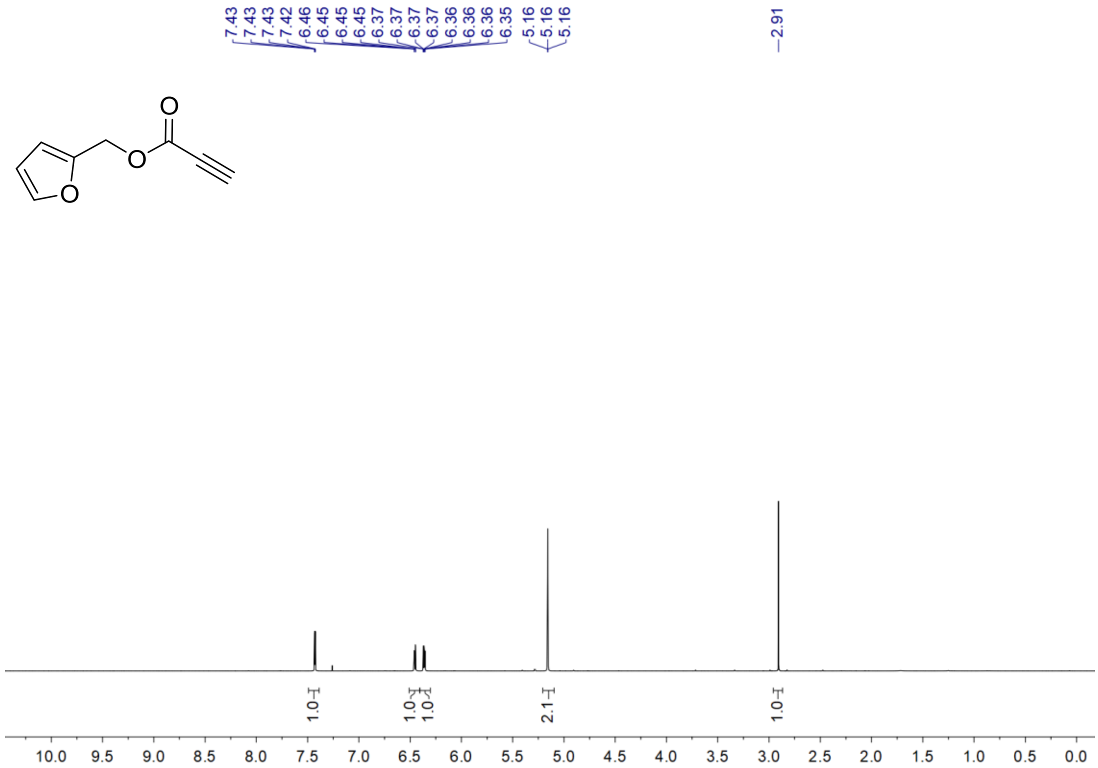

$^{13}C\{^{1}H\}$ NMR spectrum (75 MHz, $CDCl_3$) of **1f**

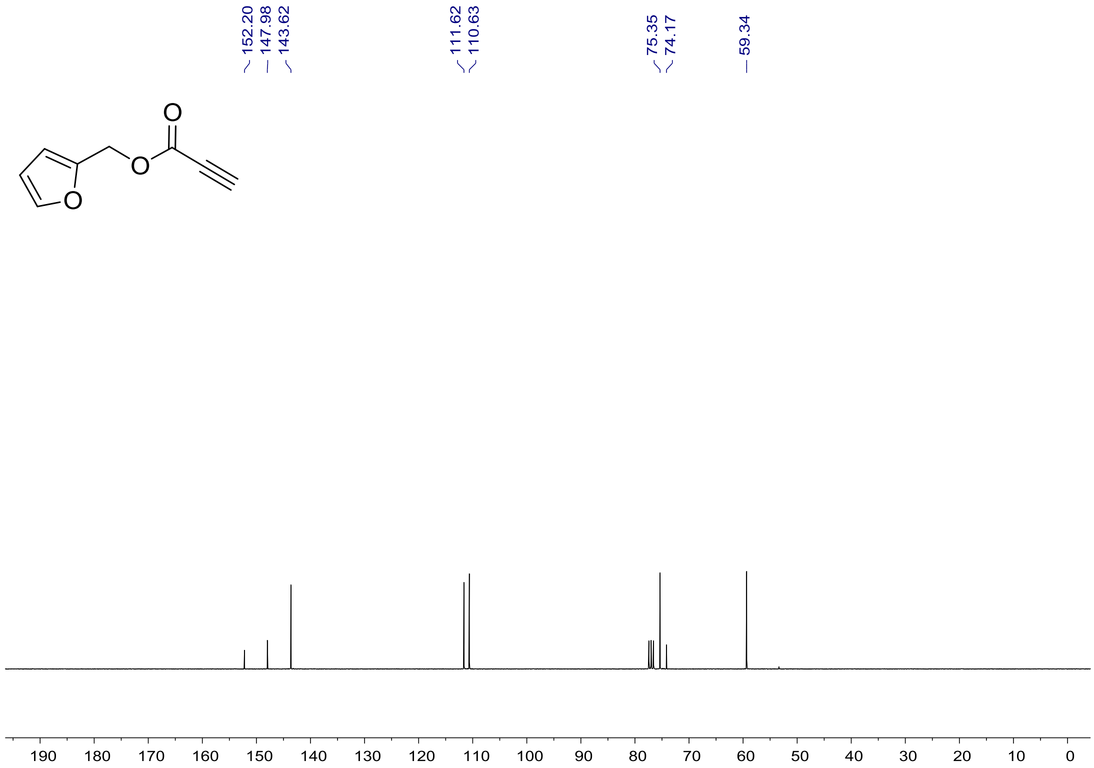

^{1}H NMR spectrum (300 MHz, $CDCl_3$) of **1g**

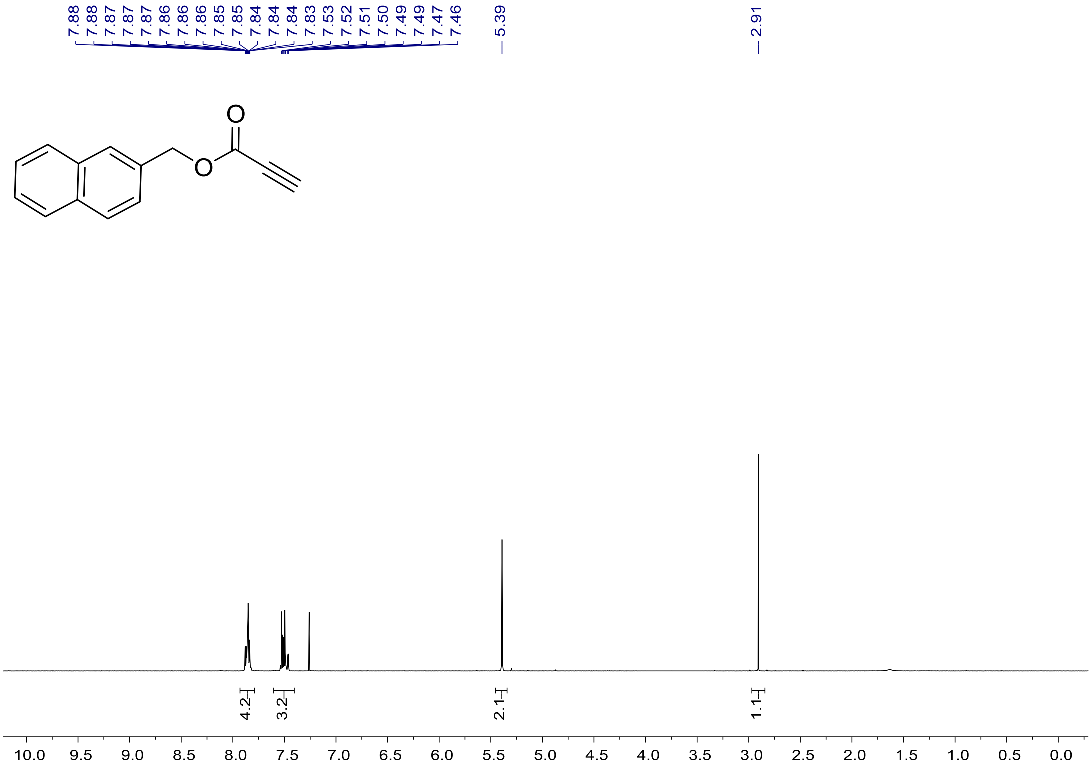

$^{13}C\{^{1}H\}$ NMR spectrum (75 MHz, $CDCl_3$) of **1g**

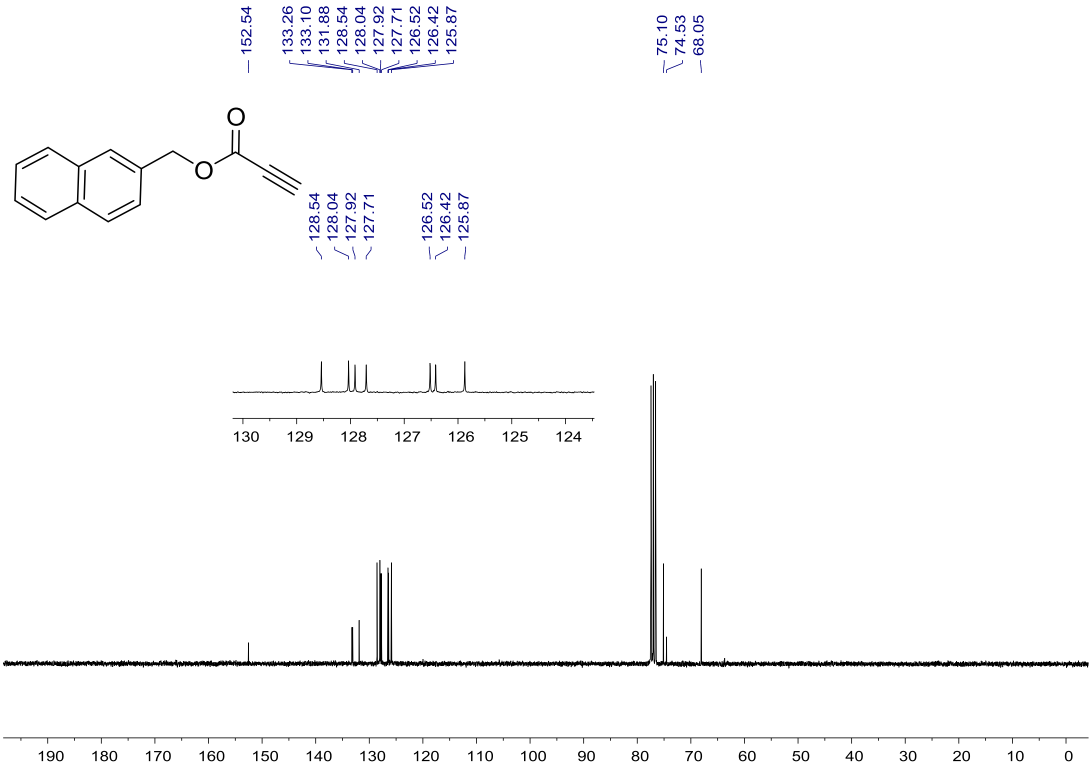

^{1}H NMR spectrum (300 MHz, $CDCl_3$) of **1h**

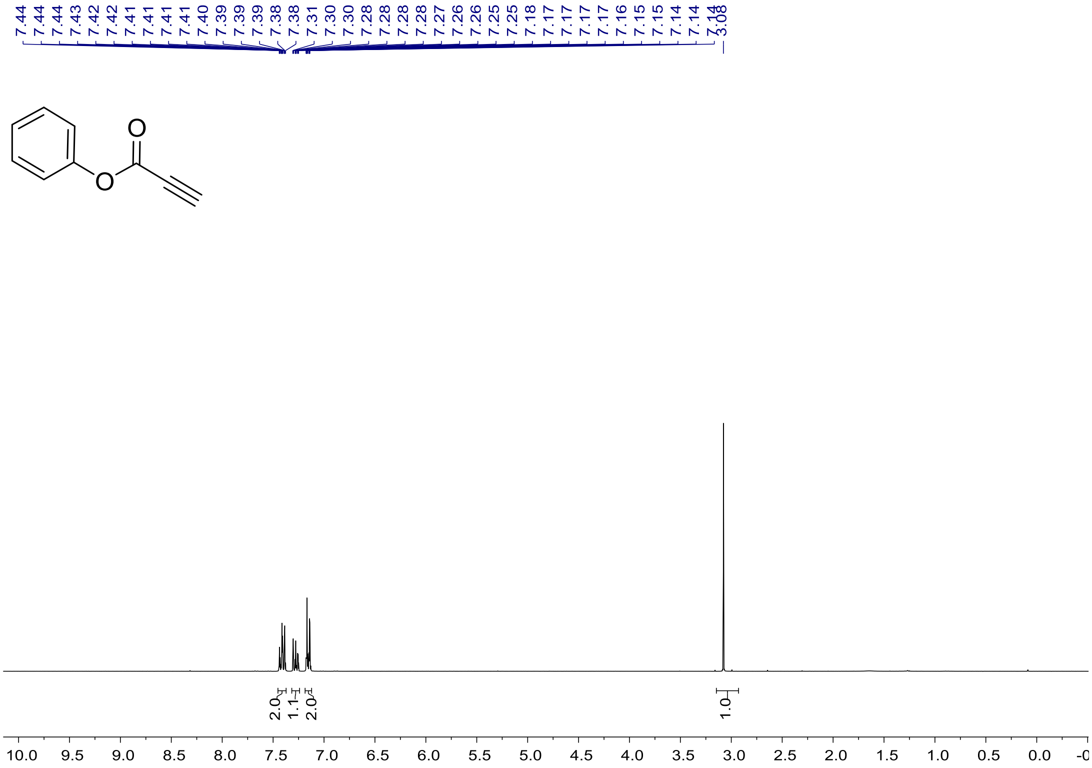

$^{13}C\{^1H\}$ NMR spectrum (75 MHz, $CDCl_3$) of **1h**

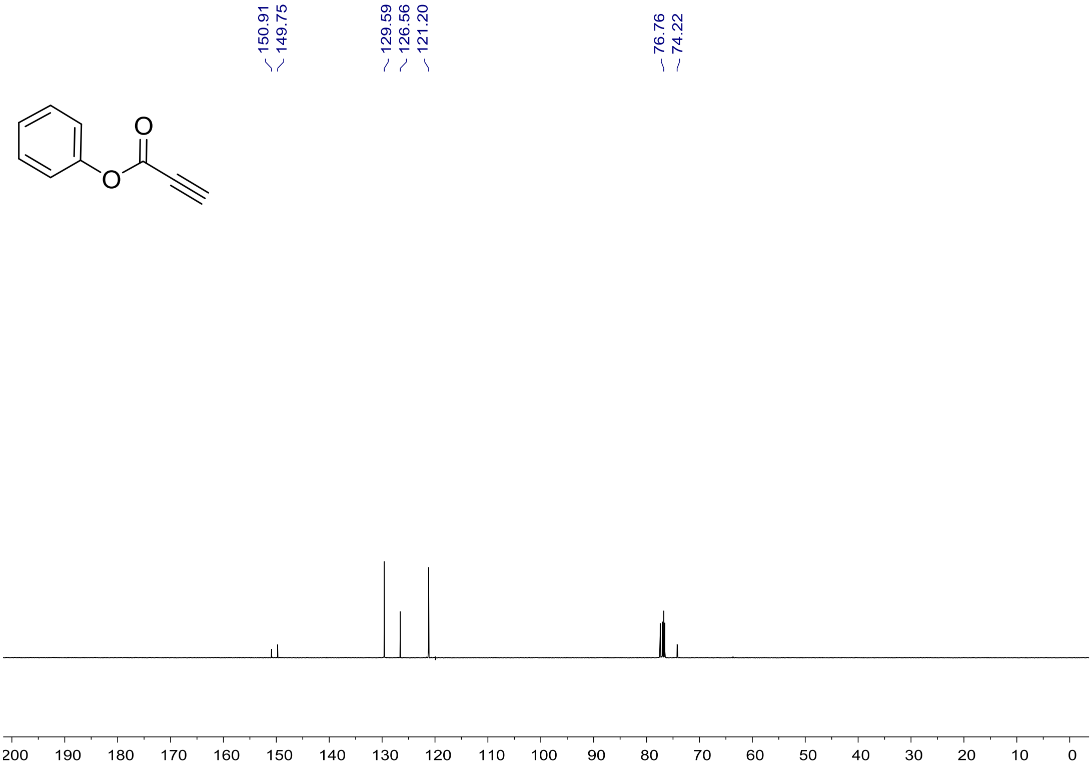

^{1}H NMR spectrum (300 MHz, $CDCl_3$) of **1j**

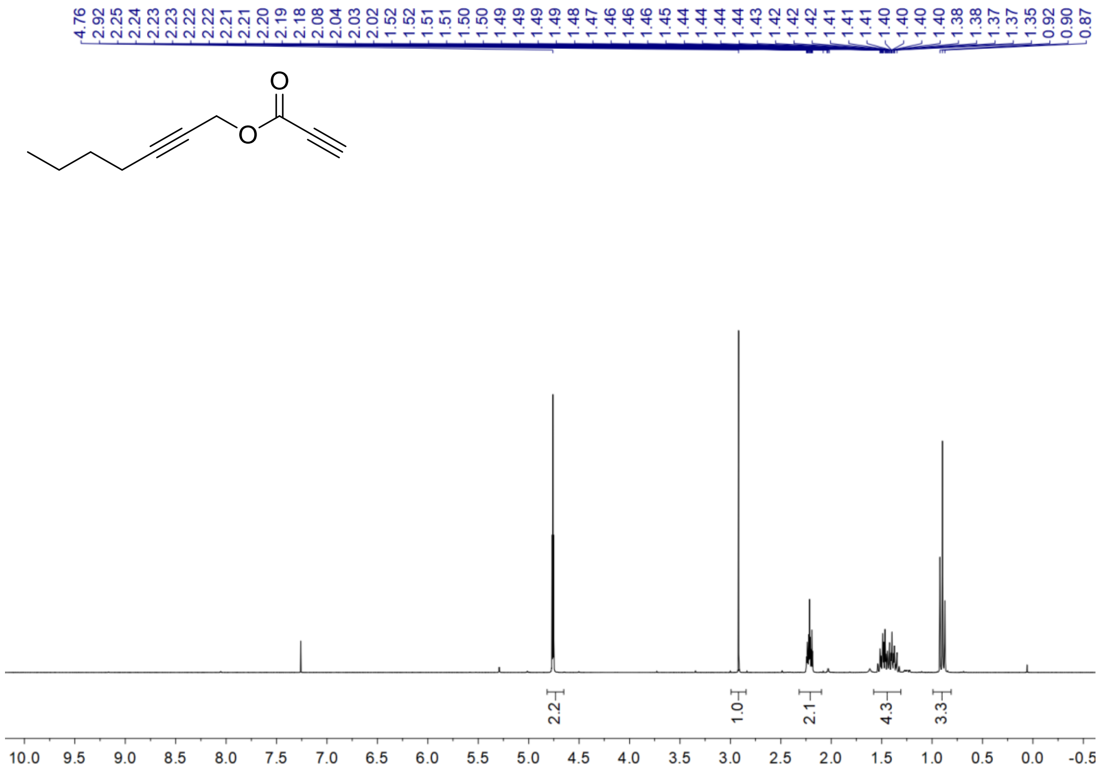

^{13}C{^{1}H} NMR spectrum (75 MHz, $CDCl_3$) of **1j**

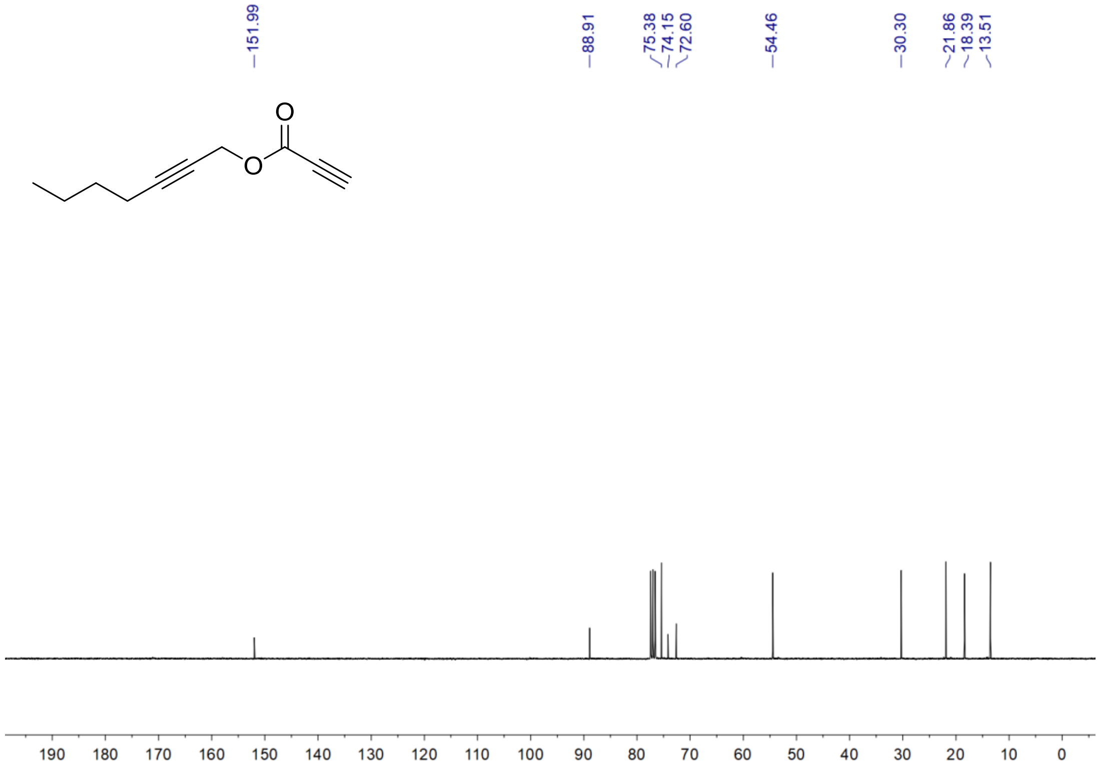

^{1}H NMR spectrum (300 MHz, $CDCl_3$) of **1k**

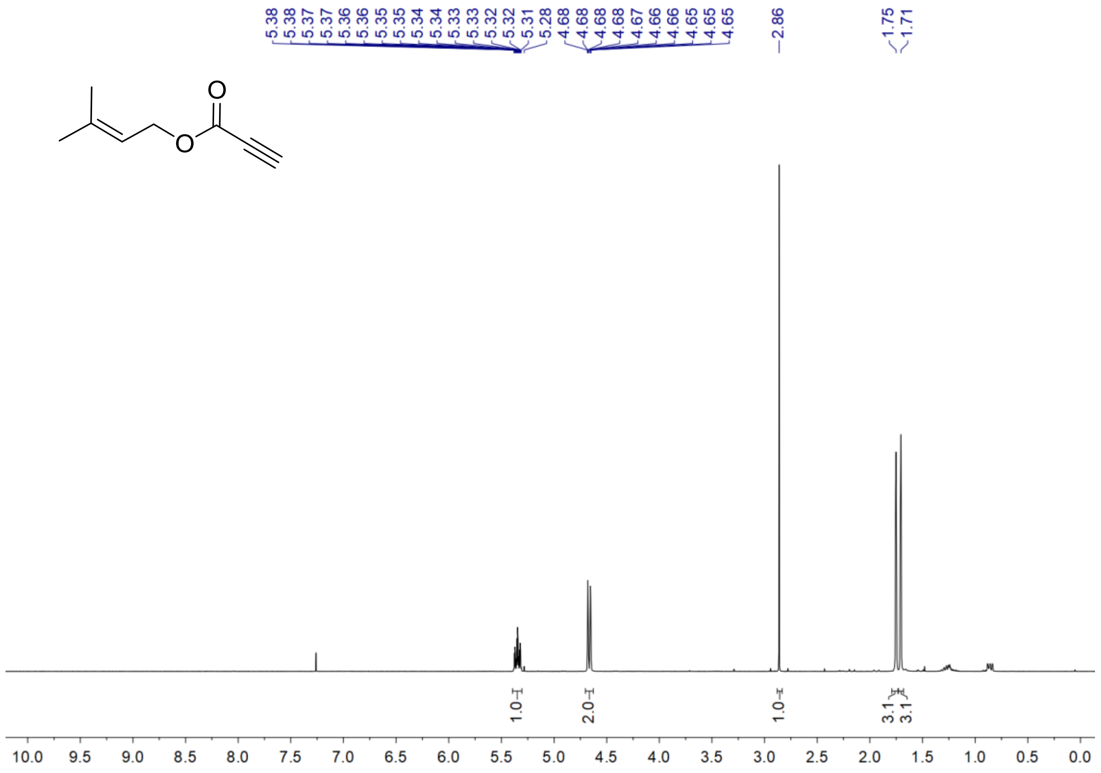

$^{13}C\{^{1}H\}$ NMR spectrum (75 MHz, $CDCl_3$) of **1k**

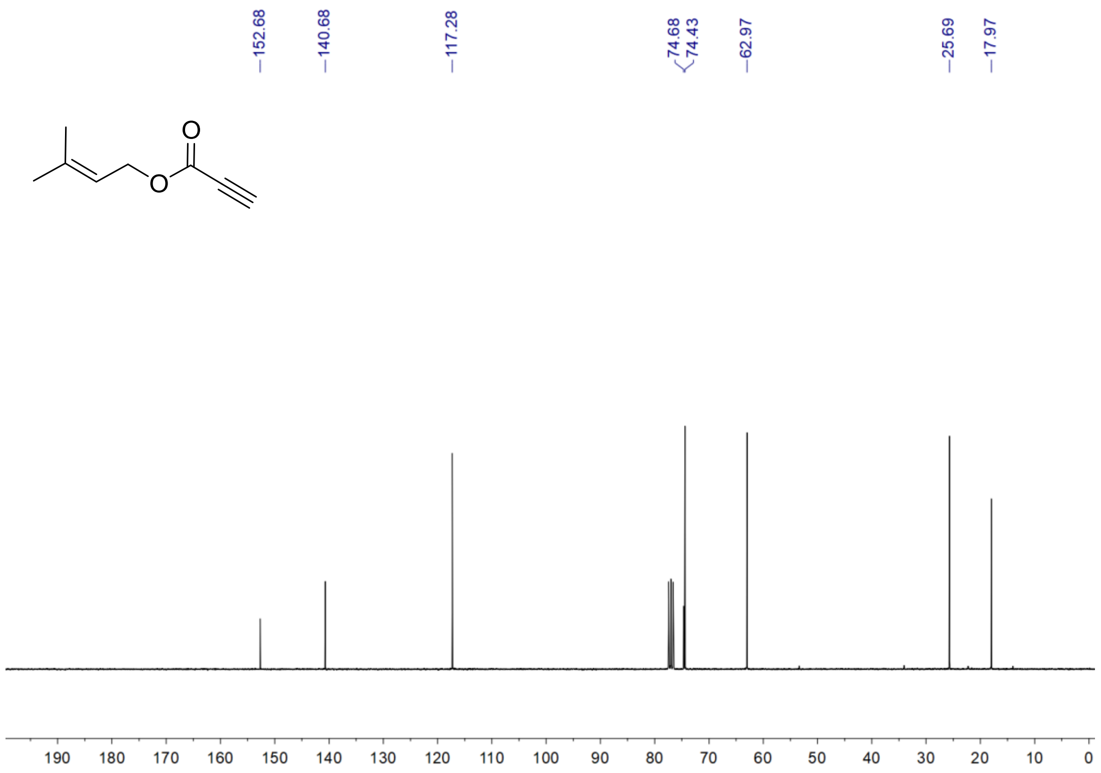

^{1}H NMR spectrum (300 MHz, $CDCl_3$) of **1n**

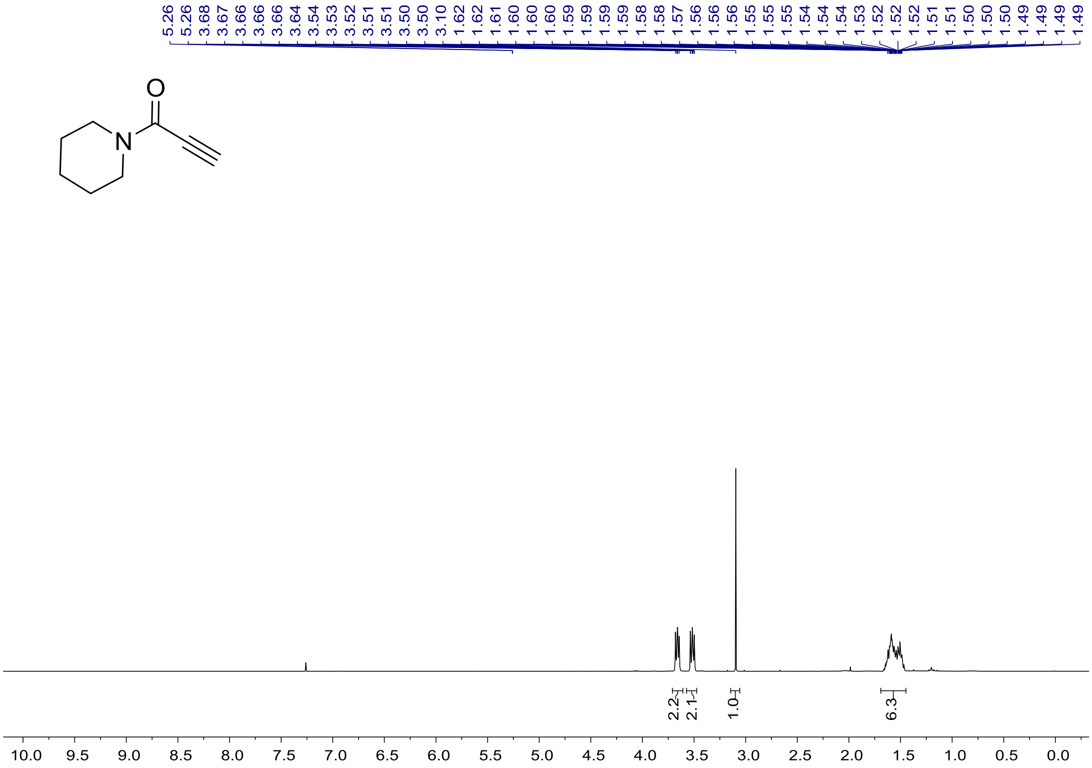

$^{13}C\{^1H\}$ NMR spectrum (75 MHz, $CDCl_3$) of **1n**

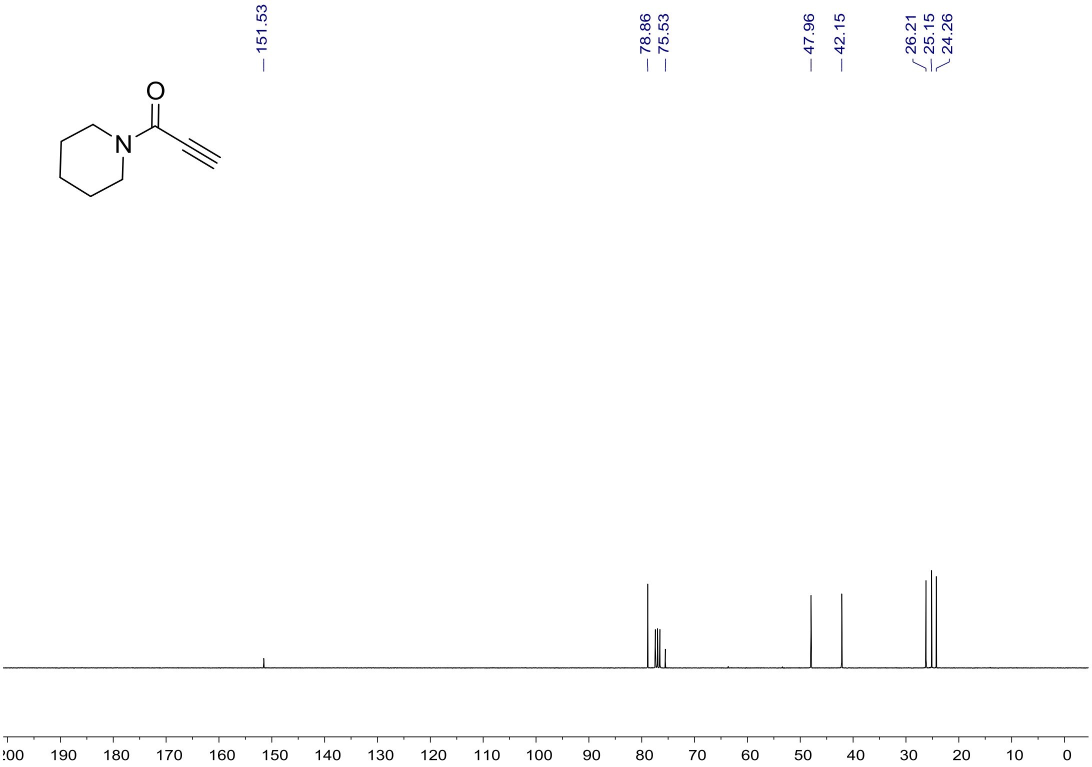

^{1}H NMR spectrum (500 MHz, $CDCl_3$) of **2a**

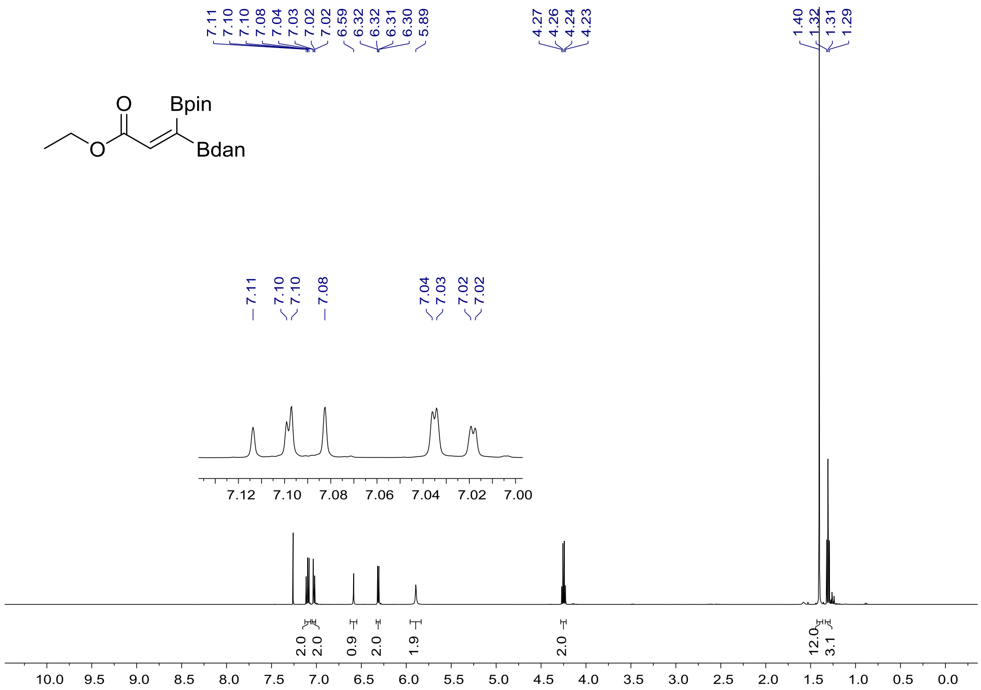

^{13}C{^{1}H} NMR spectrum (126 MHz, $CDCl_3$) of **2a**

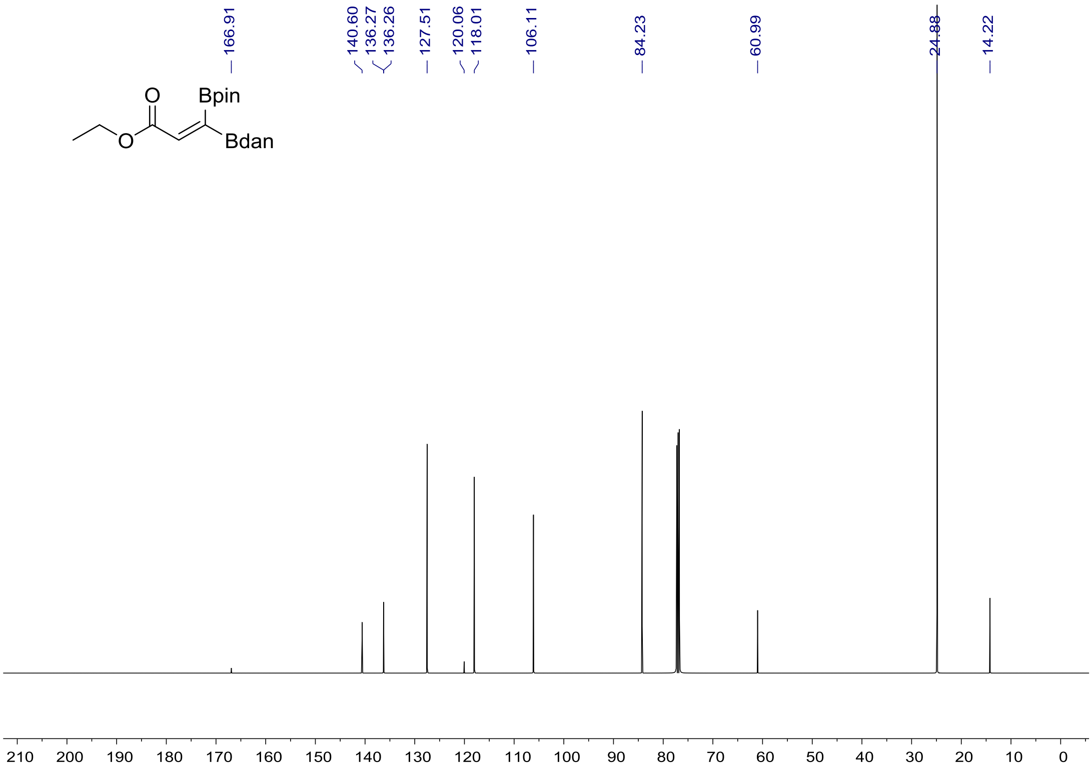

^{11}B NMR spectrum (160 MHz, CDCl$_3$) of **2a**

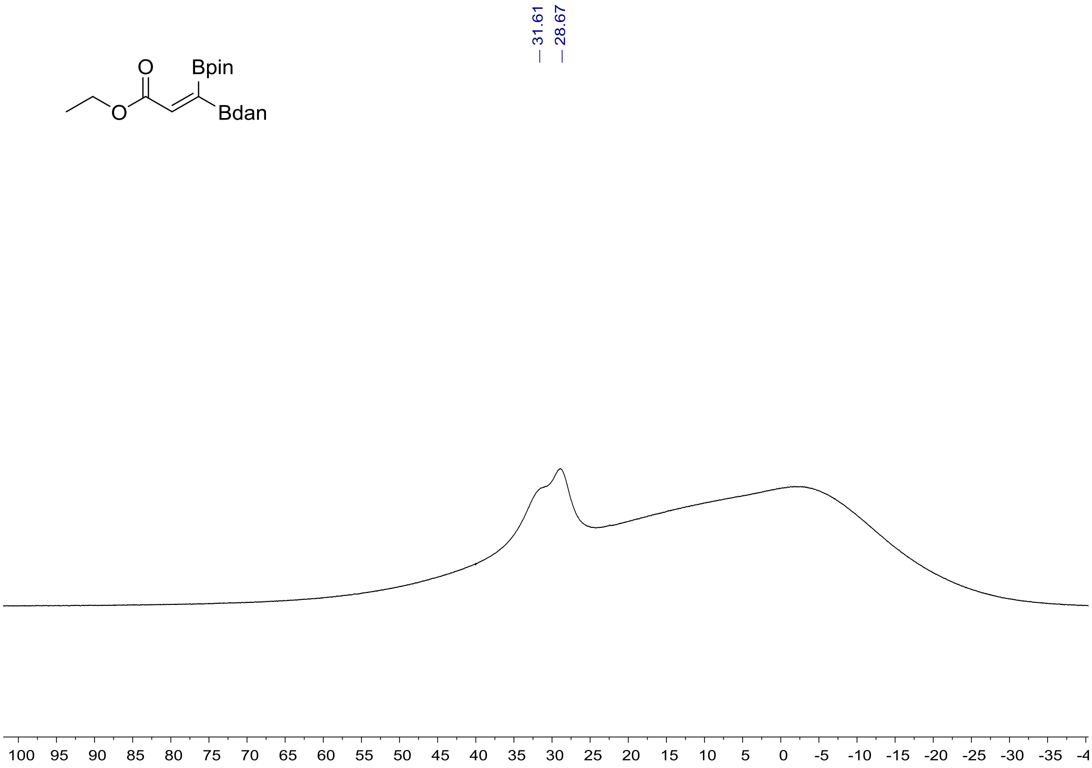

^{1}H NMR spectrum (500 MHz, CDCl$_3$) of **2b**

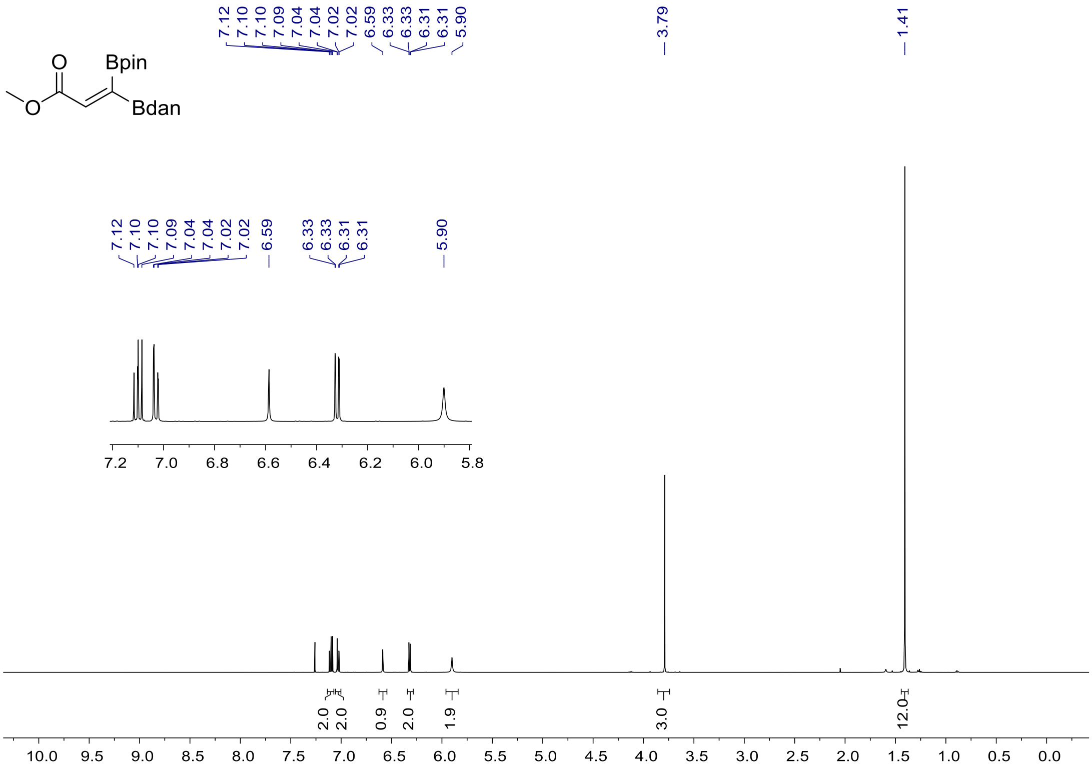

$^{13}C\{^1H\}$ NMR spectrum (126 MHz, $CDCl_3$) of **2b**

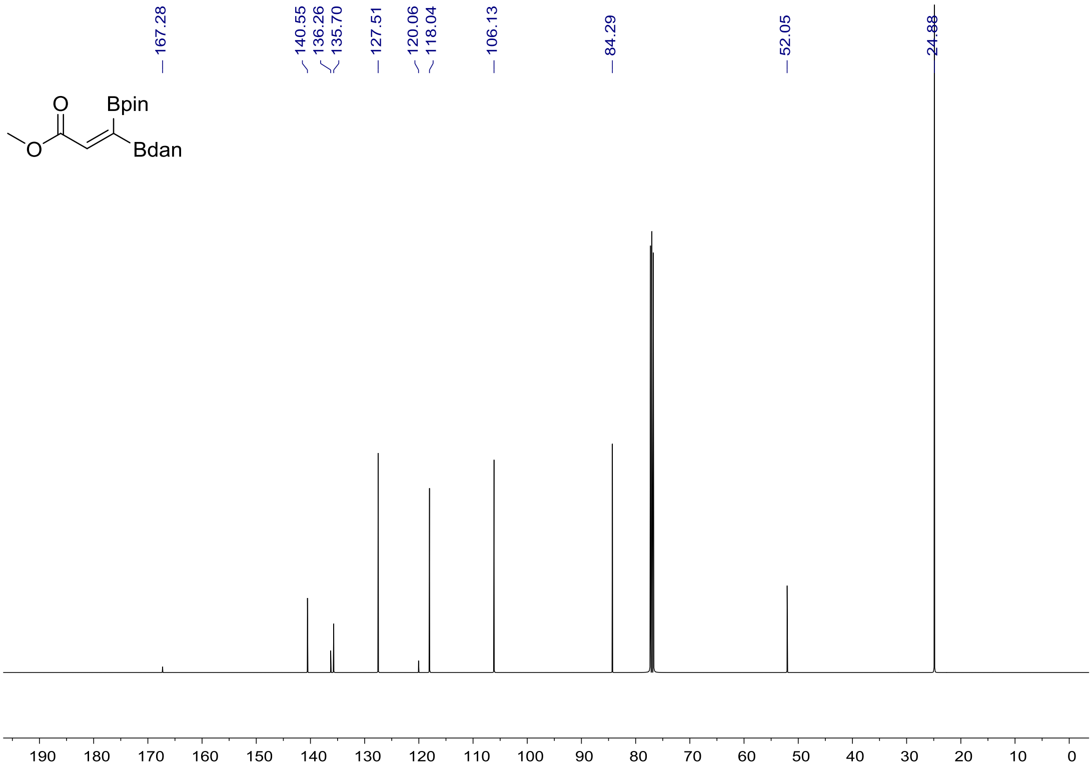

^{11}B NMR spectrum (160 MHz, $CDCl_3$) of **2b**

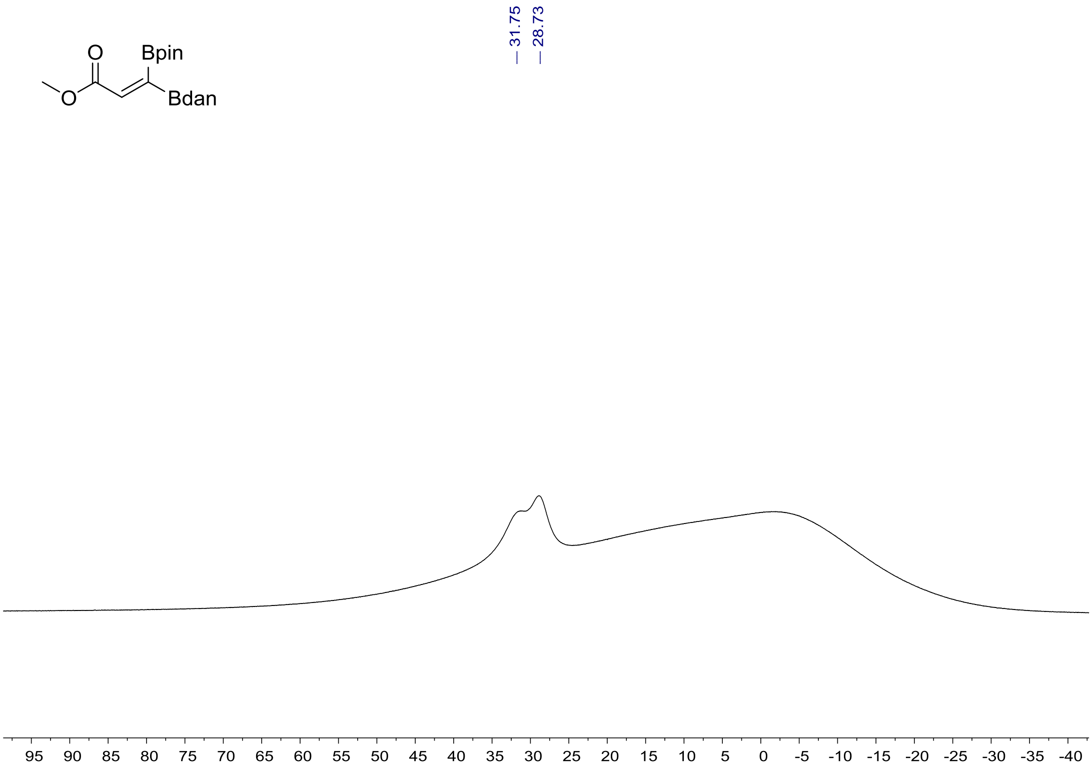

^{1}H NMR spectrum (500 MHz, $CDCl_3$) of **2c**

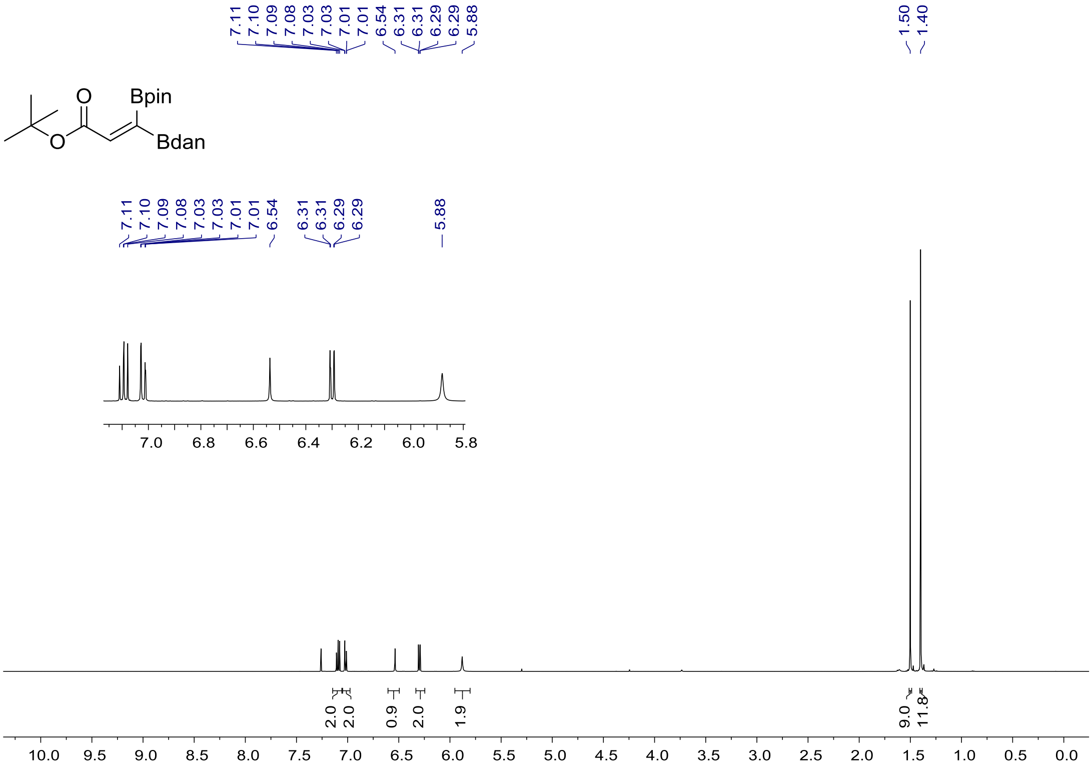

^{13}C{^{1}H} NMR spectrum (126 MHz, $CDCl_3$) of **2c**

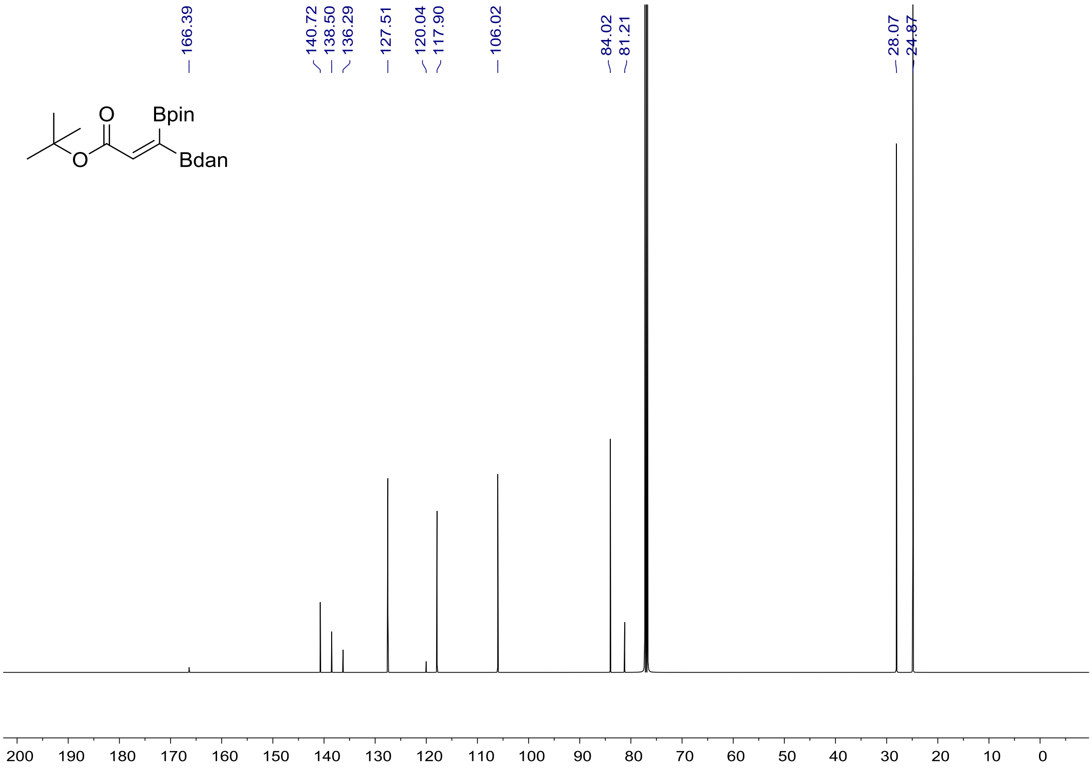

^{11}B NMR spectrum (160 MHz, $CDCl_3$) of **2c**

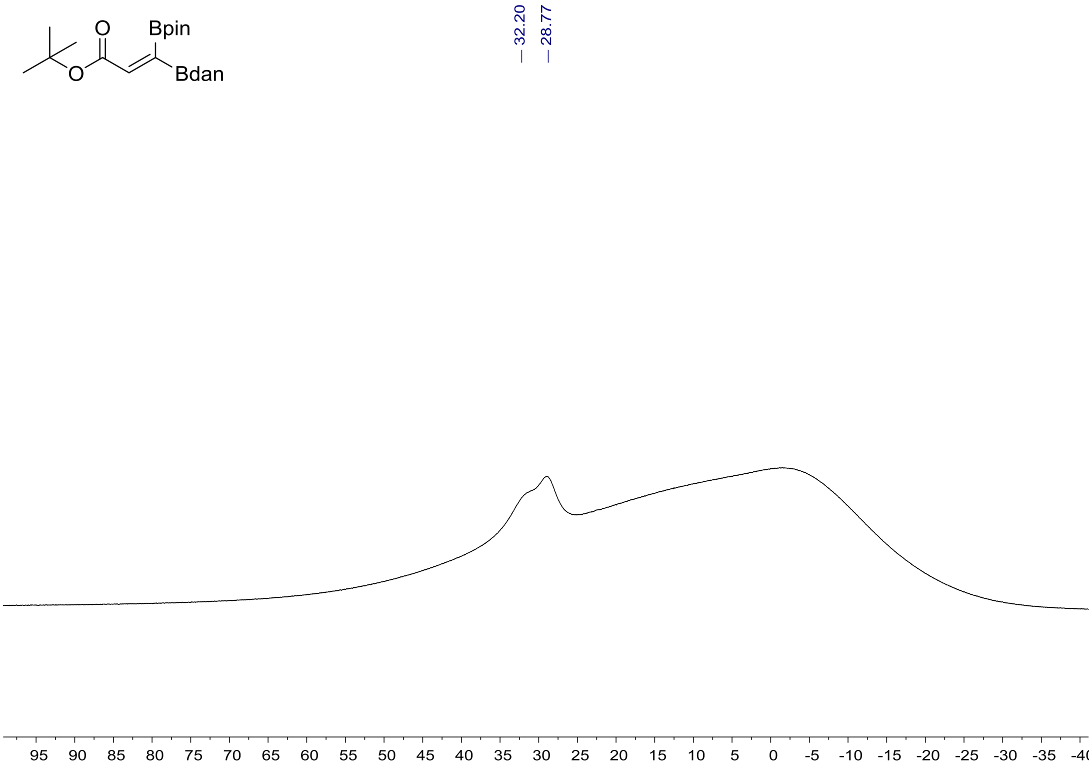

^{1}H NMR spectrum (500 MHz, $CDCl_3$) of **2d**

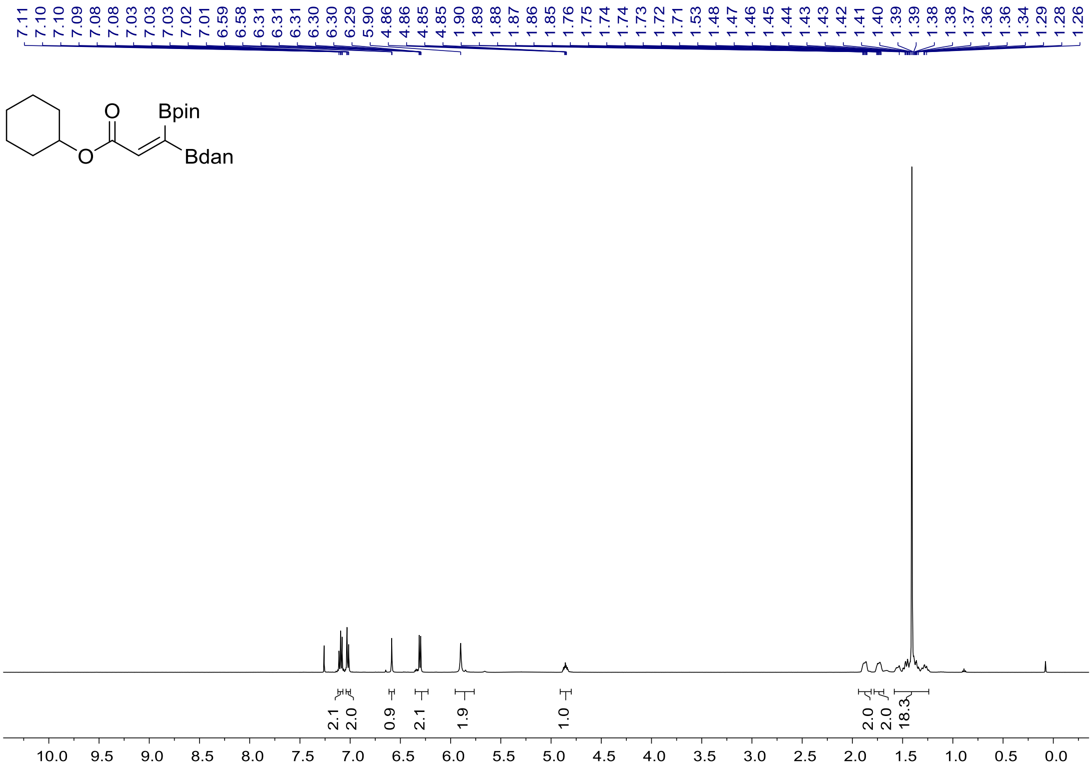

$^{13}C\{^{1}H\}$ NMR spectrum (126 MHz, $CDCl_3$) of **2d**

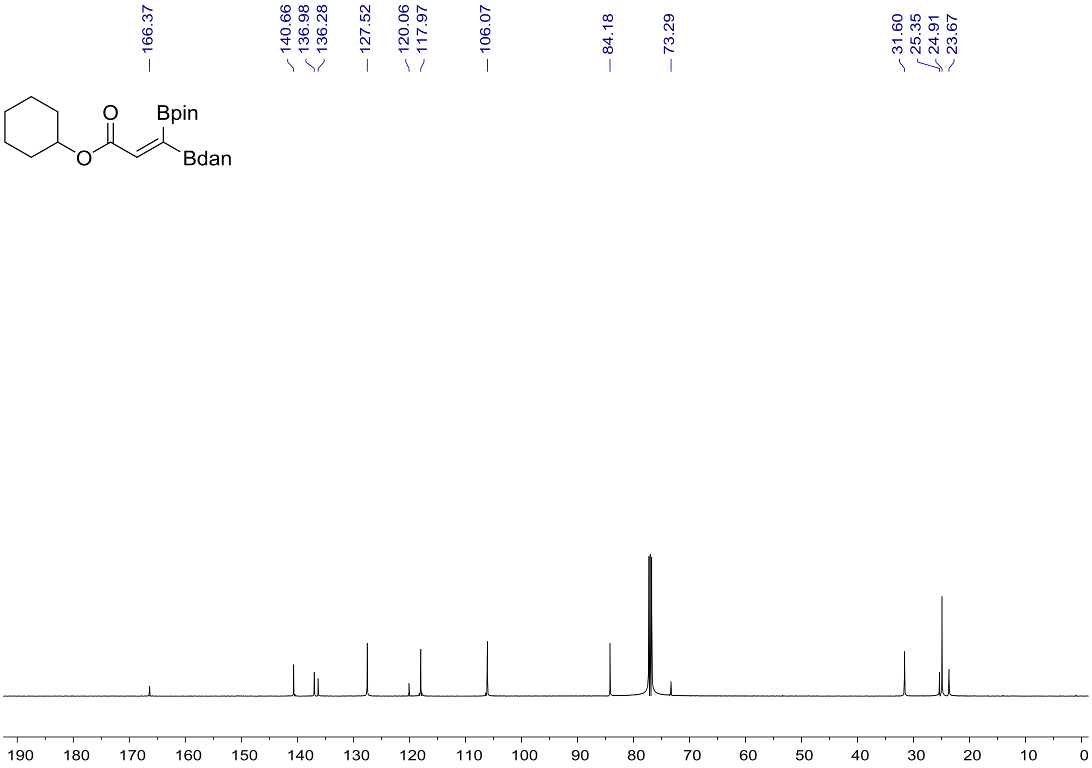

^{11}B NMR spectrum (160 MHz, $CDCl_3$) of **2d**

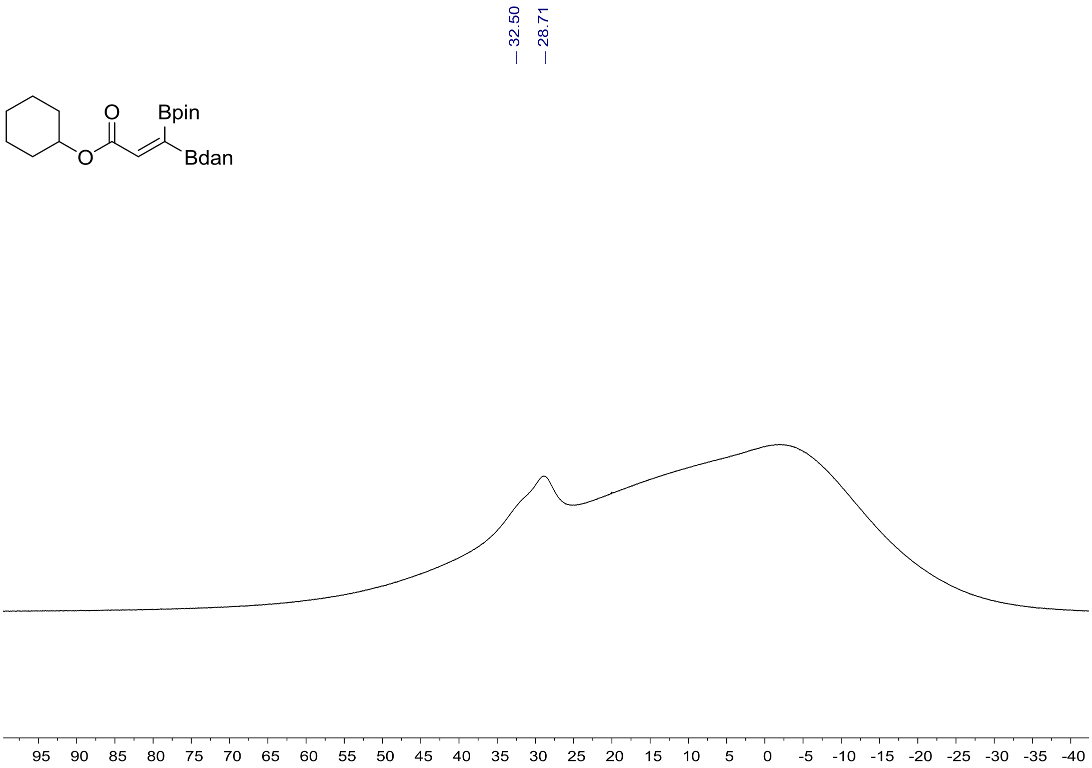

^{1}H NMR spectrum (500 MHz, $CDCl_3$) of **2e**

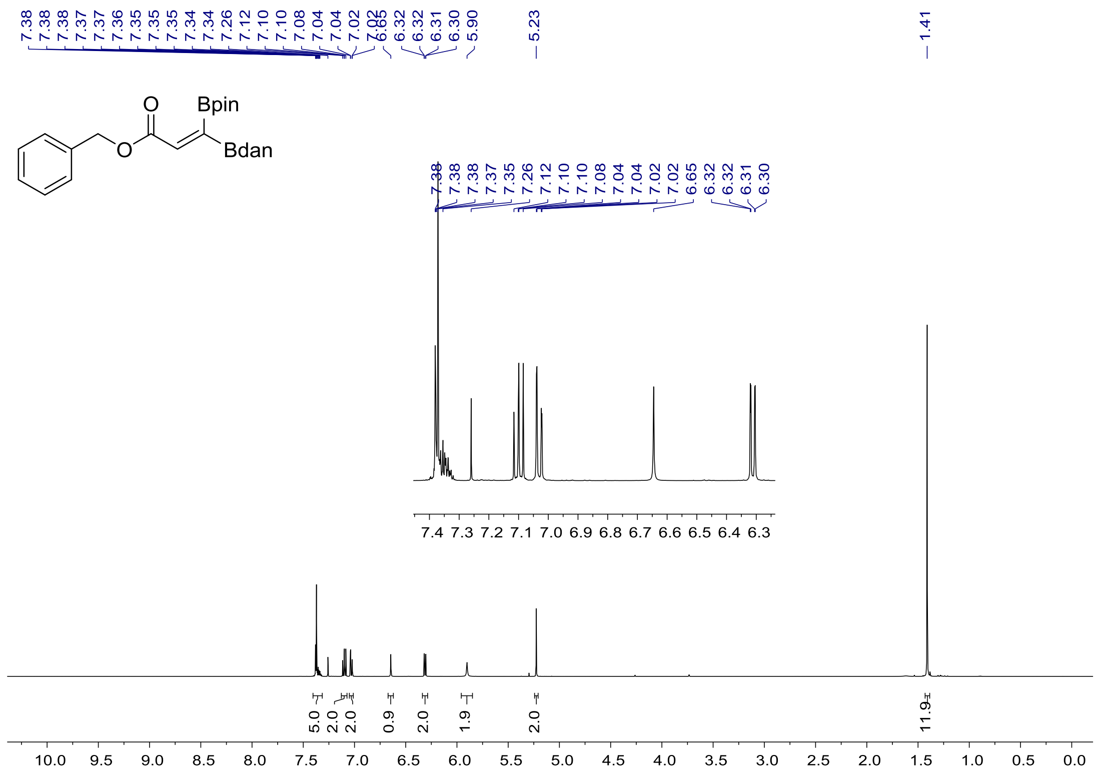

$^{13}C\{^{1}H\}$ NMR spectrum (126 MHz, $CDCl_3$) of **2e**

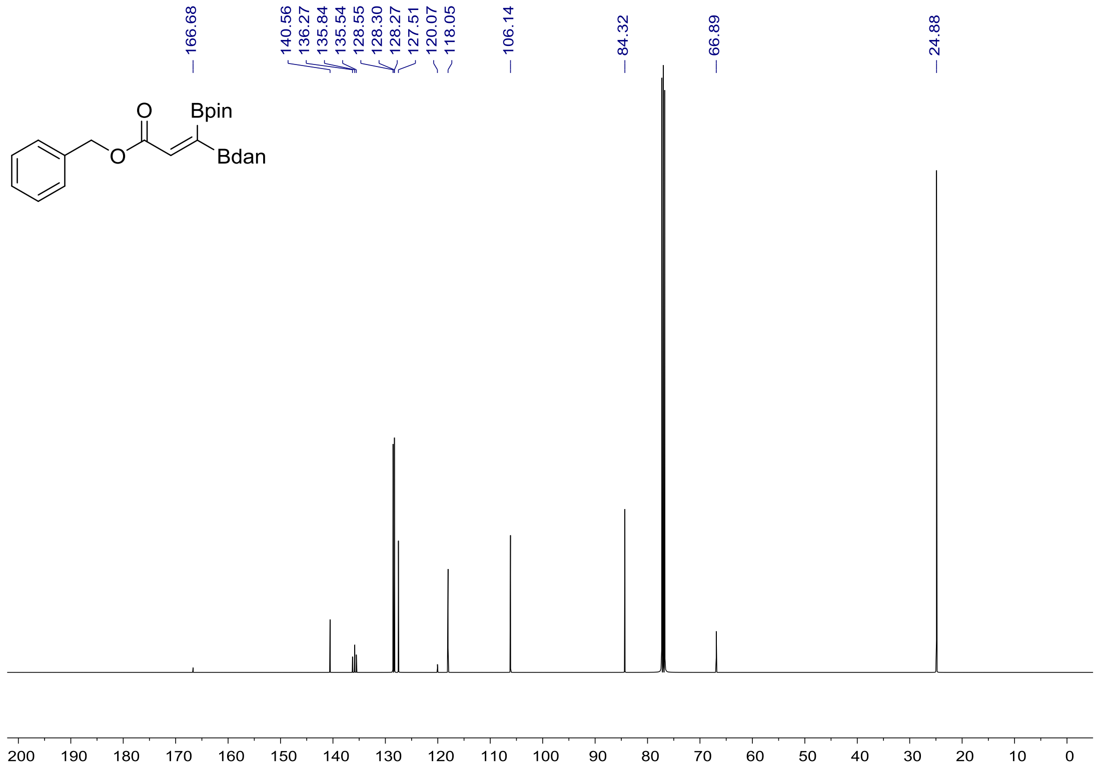

^{11}B NMR spectrum (96 MHz, $CDCl_3$) of **2e**

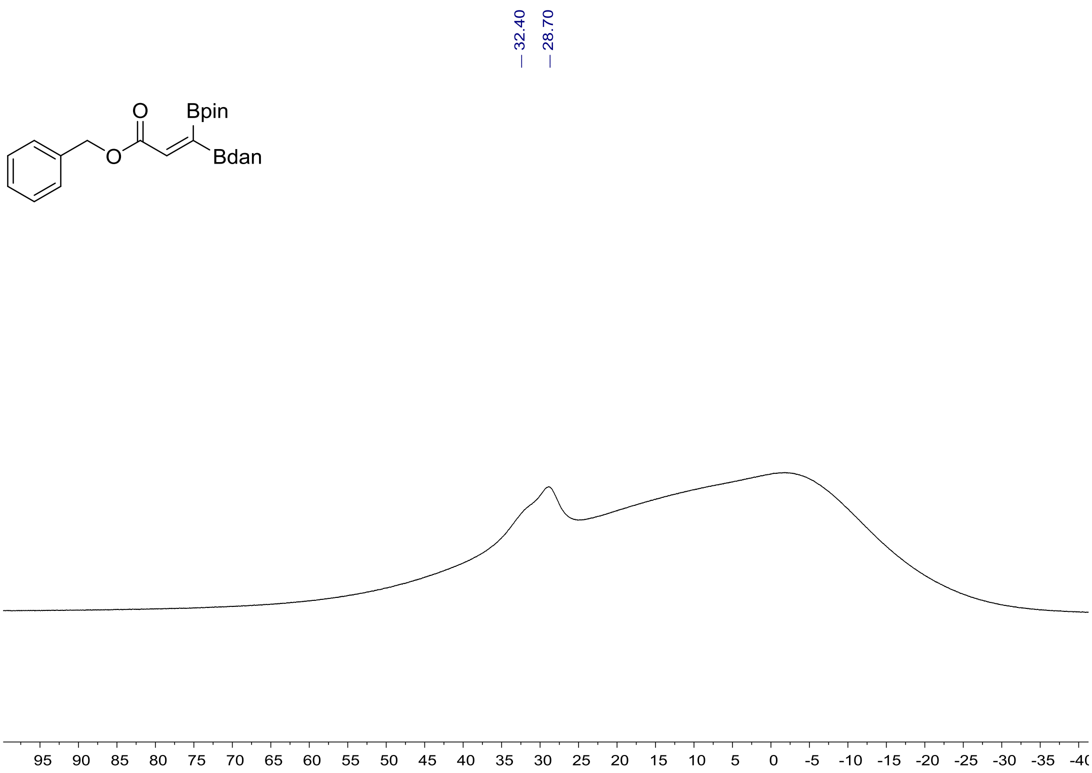

^{1}H NMR spectrum (500 MHz, $CDCl_3$) of **2f**

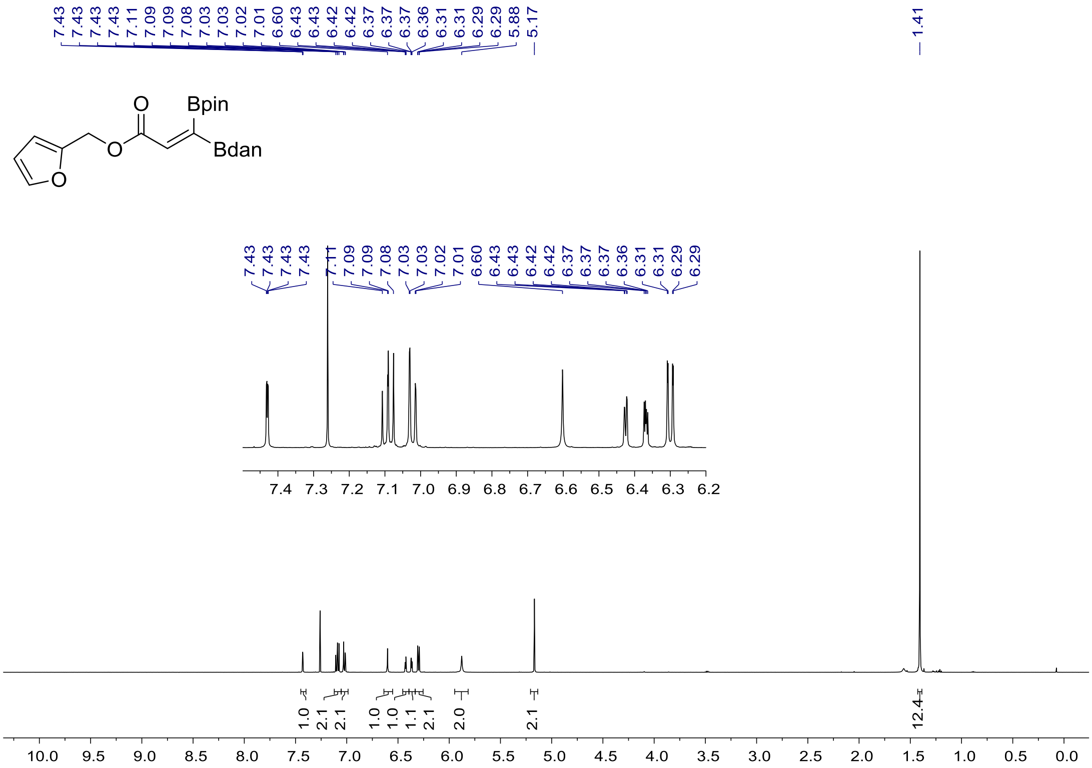

$^{13}C\{^1H\}$ NMR spectrum (126 MHz, $CDCl_3$) of **2f**

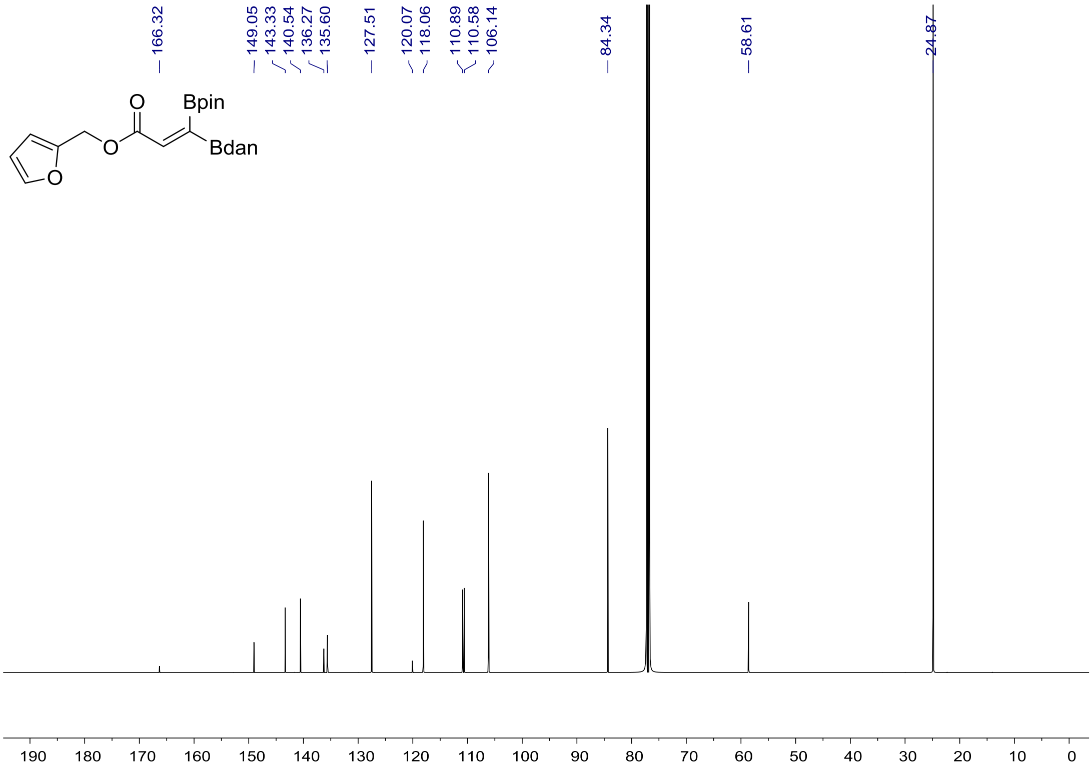

^{11}B NMR spectrum (160 MHz, $CDCl_3$) of **2f**

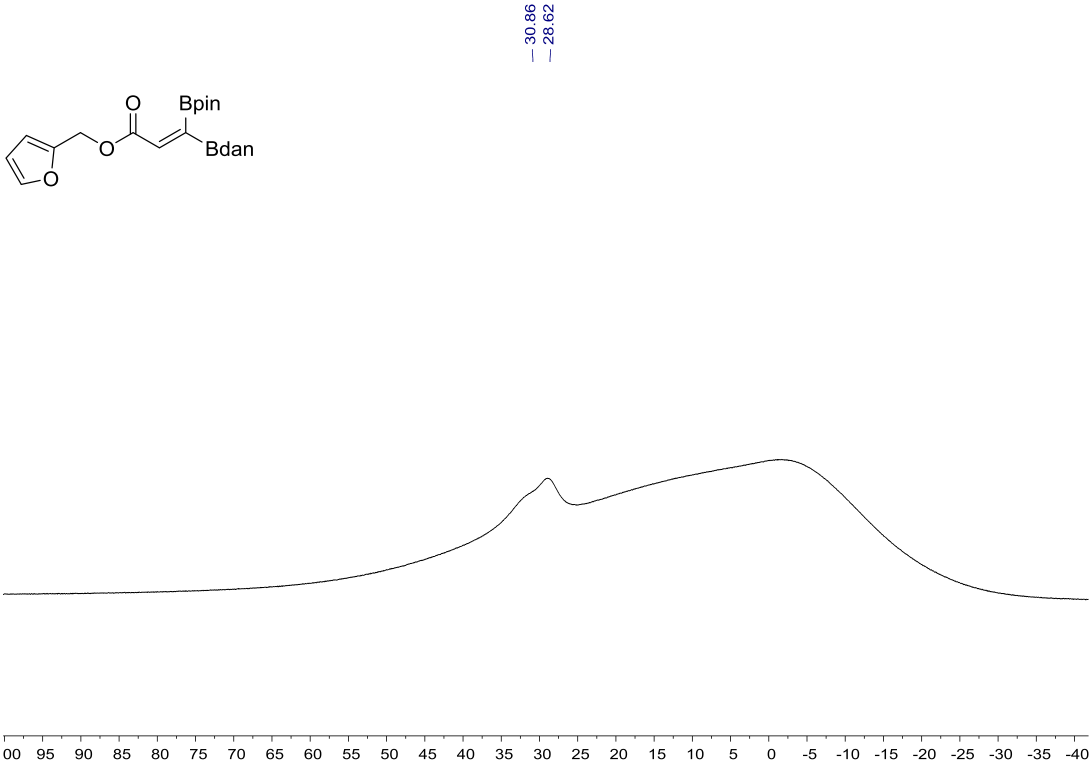

^{1}H NMR spectrum (500 MHz, $CDCl_3$) of **2g**

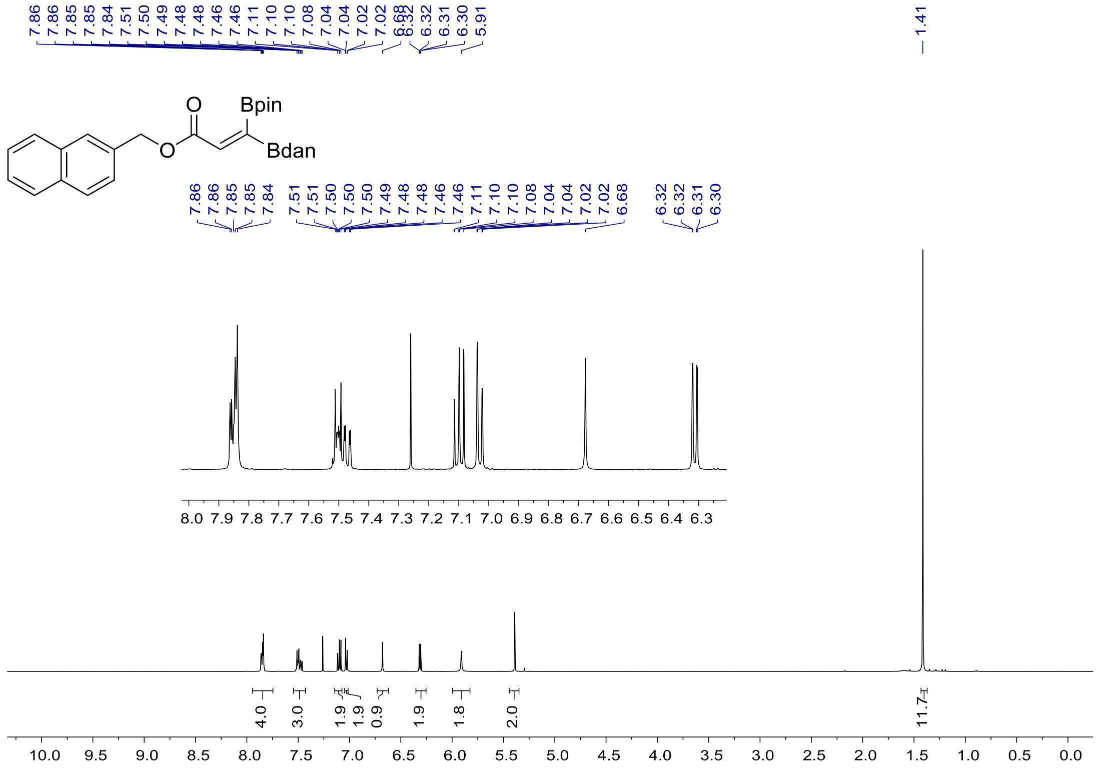

$^{13}C\{^{1}H\}$ NMR spectrum (126 MHz, $CDCl_3$) of **2g**

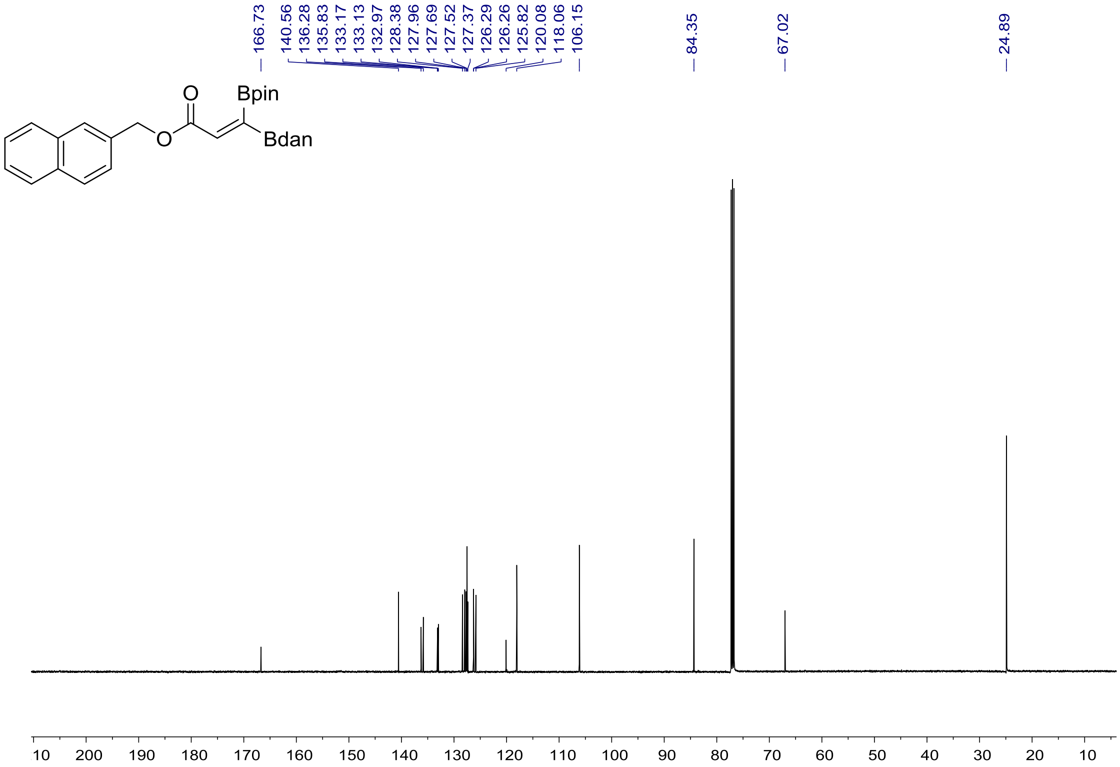

^{11}B NMR spectrum (160 MHz, $CDCl_3$) of **2g**

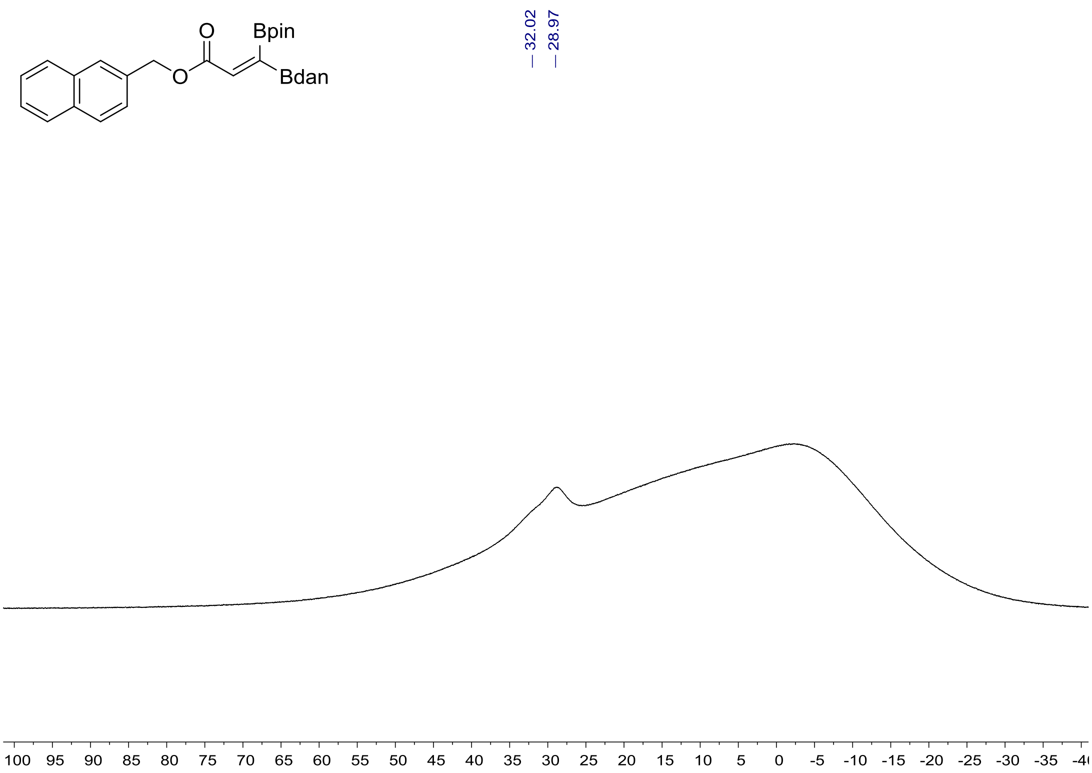

^{1}H NMR spectrum (500 MHz, $CDCl_3$) of **2h**

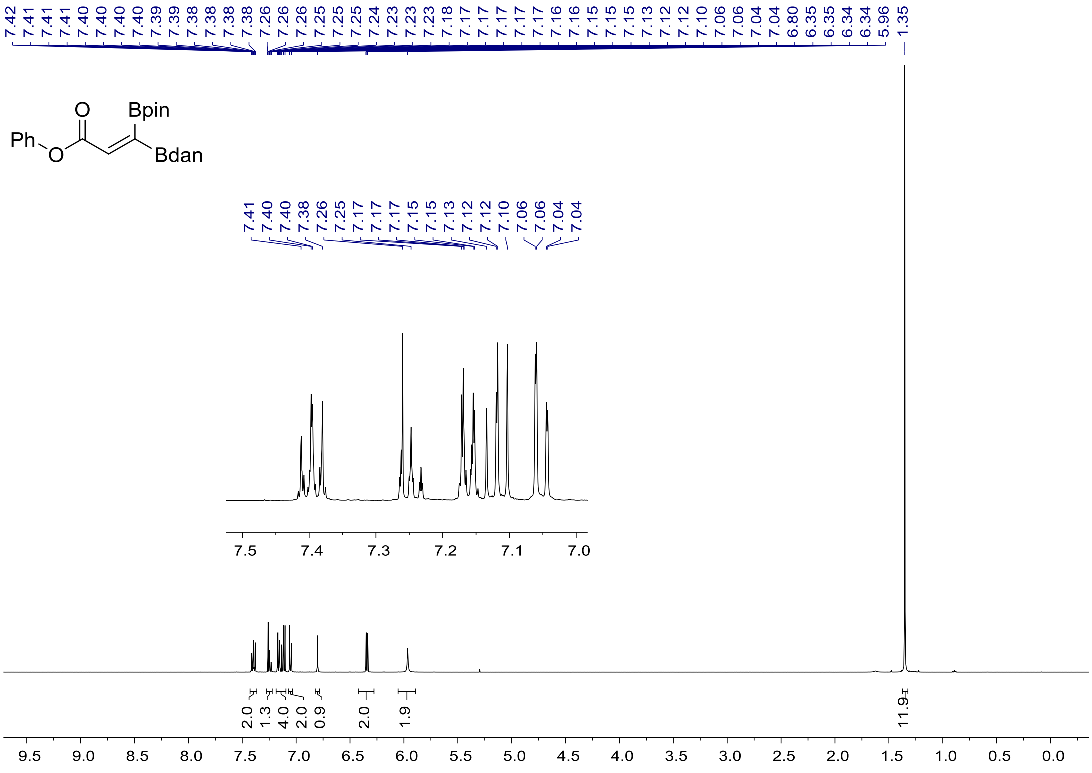

$^{13}C\{^1H\}$ NMR spectrum (126 MHz, $CDCl_3$) of **2h**

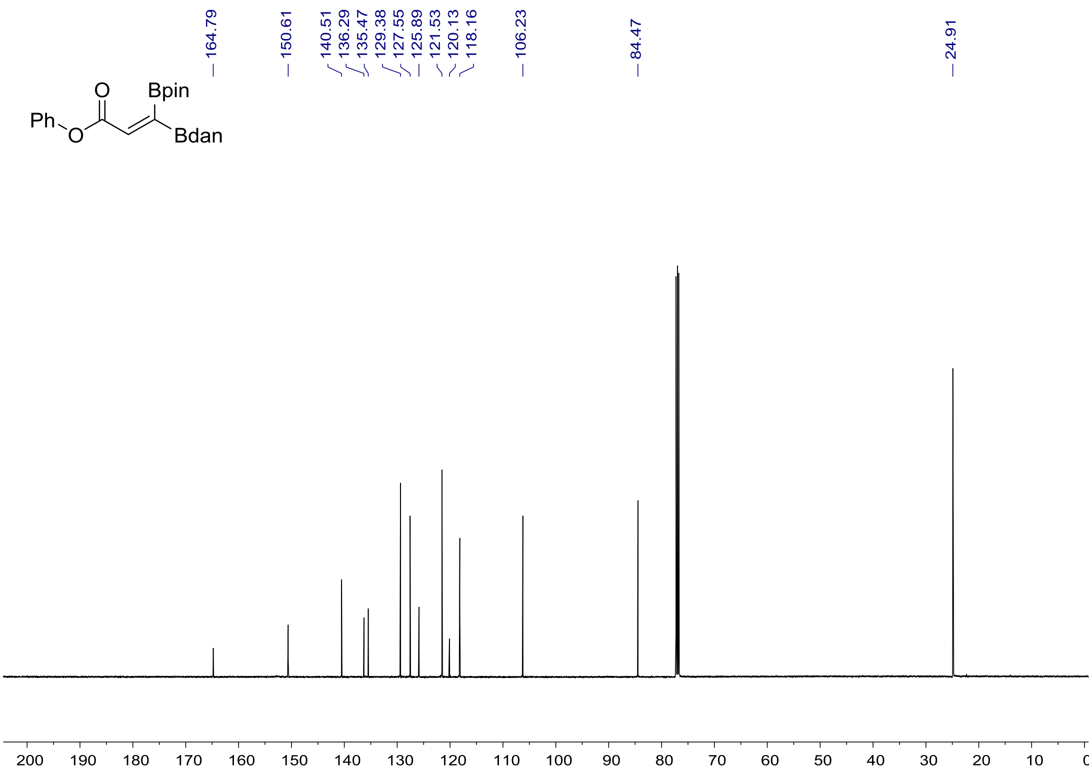

^{11}B NMR spectrum (160 MHz, $CDCl_3$) of **2h**

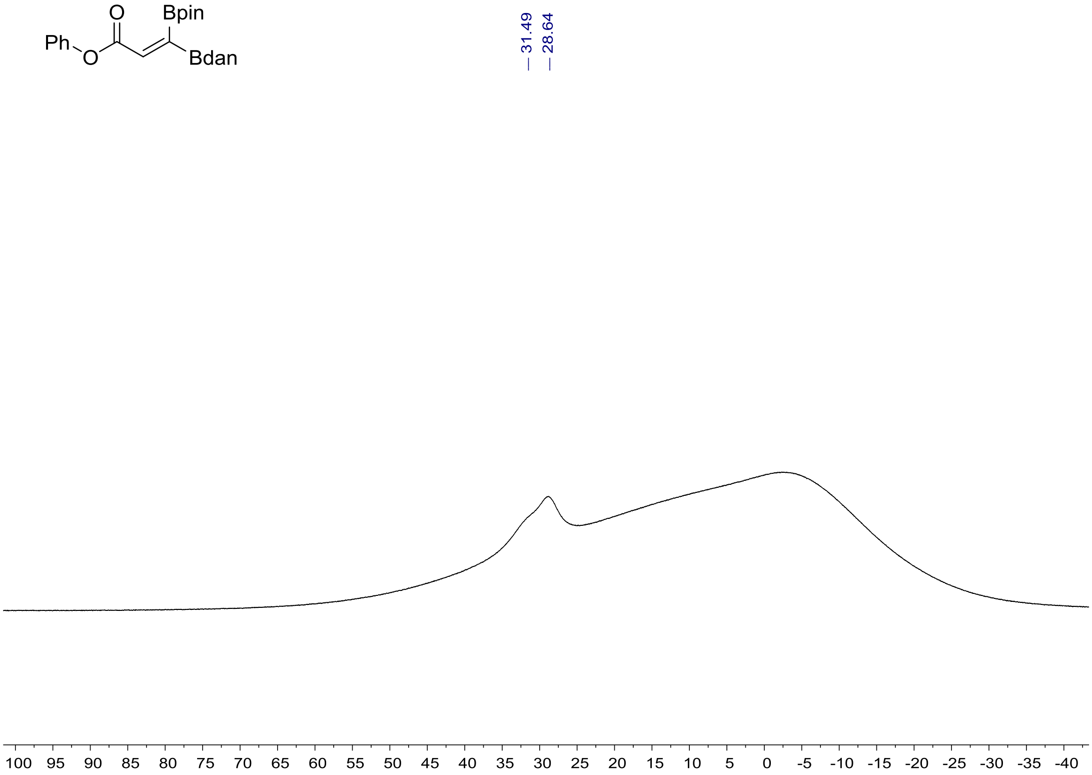

^{1}H NMR spectrum (500 MHz, $CDCl_3$) of **2i**

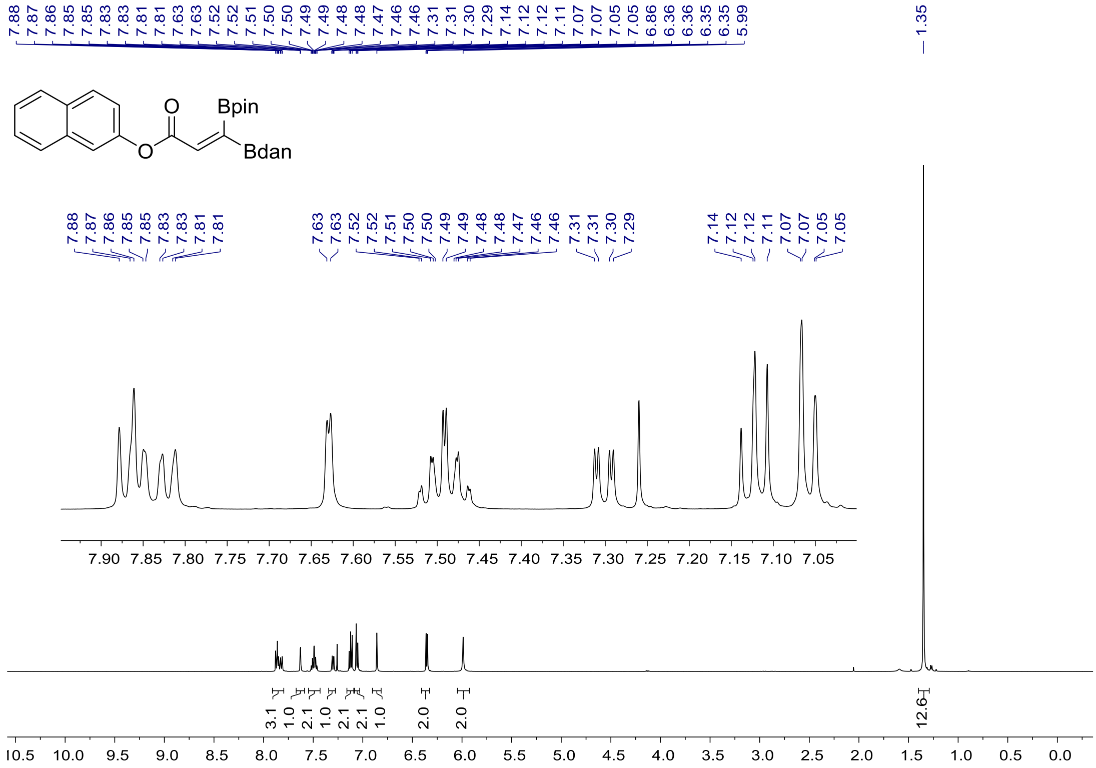

$^{13}C\{^{1}H\}$ NMR spectrum (126 MHz, $CDCl_3$) of **2i**

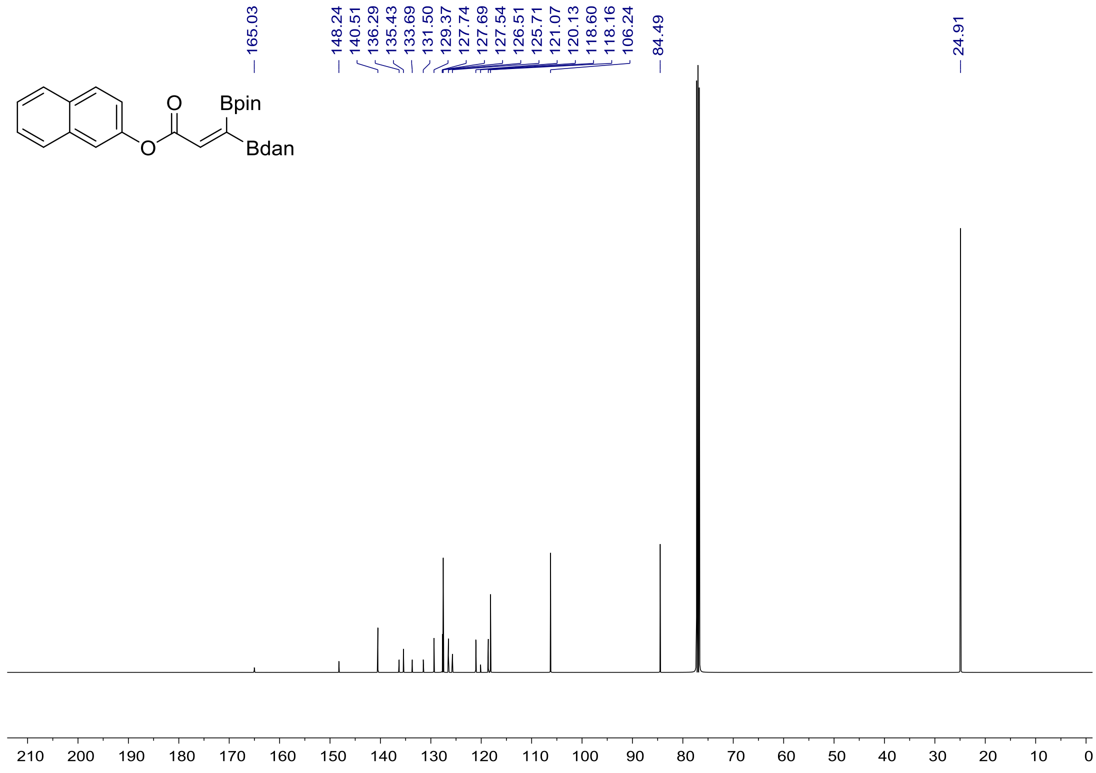

^{11}B NMR spectrum (160 MHz, $CDCl_3$) of **2i**

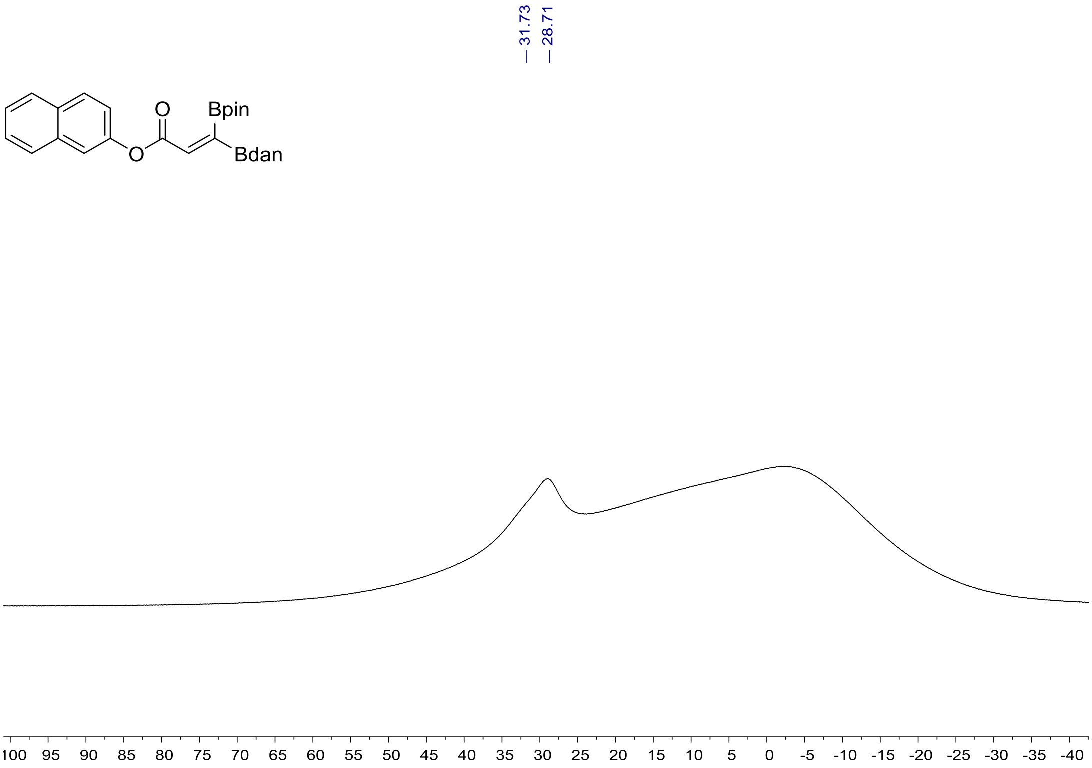

^{1}H NMR spectrum (500 MHz, $CDCl_3$) of **2j**

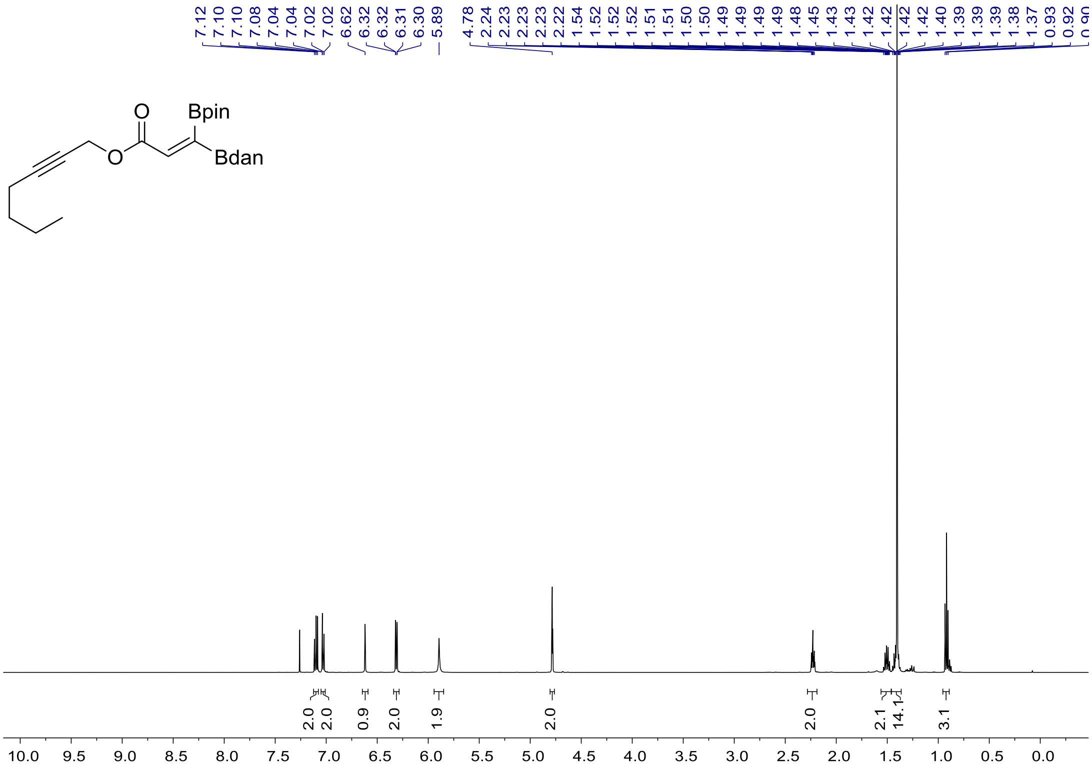

$^{13}C\{^1H\}$ NMR spectrum (126 MHz, $CDCl_3$) of **2j**

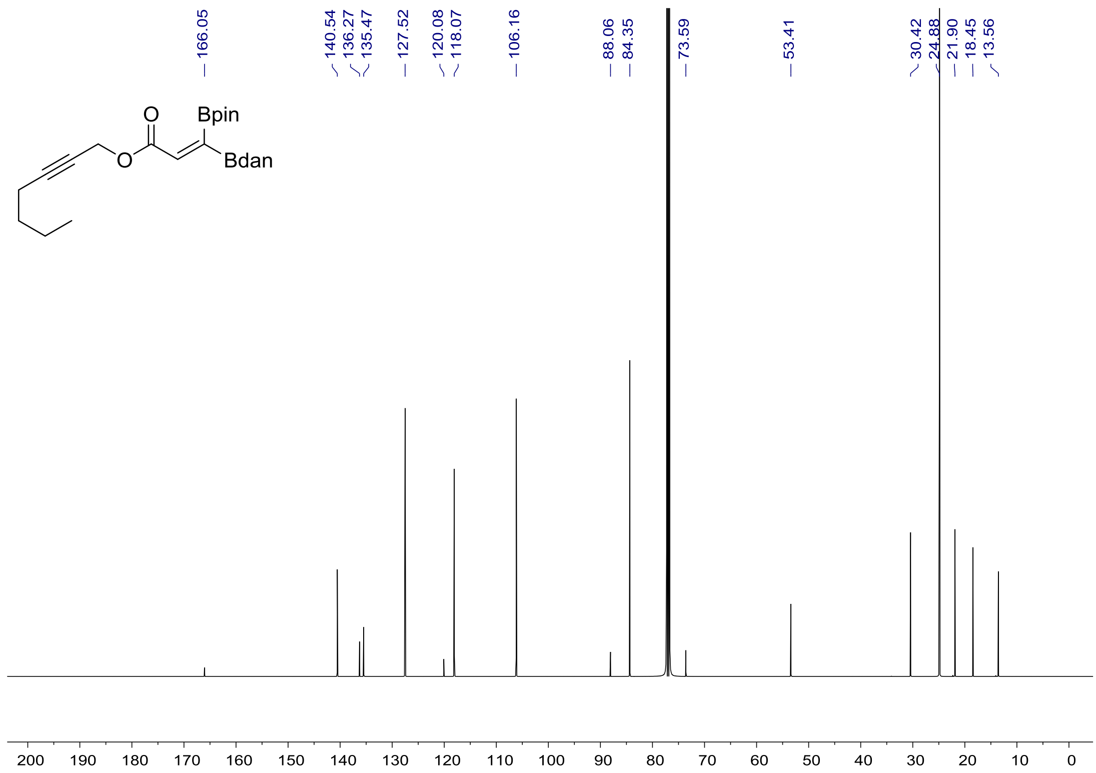

^{11}B NMR spectrum (160 MHz, $CDCl_3$) of **2j**

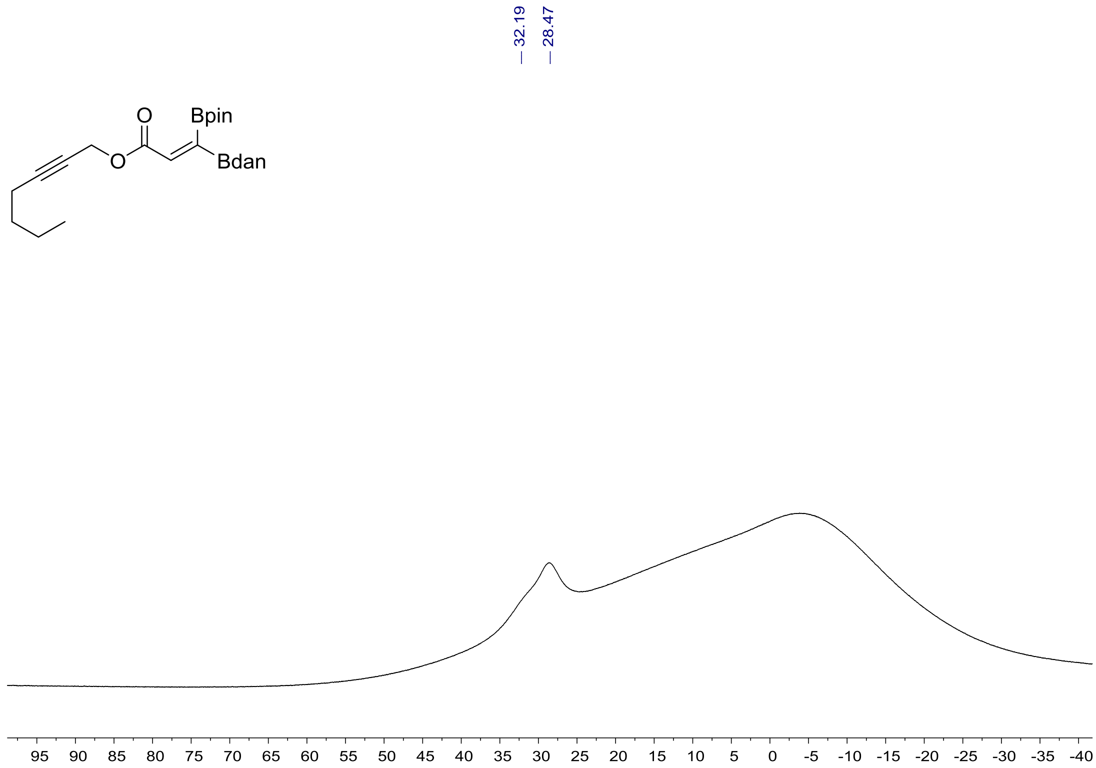

^{1}H NMR spectrum (500 MHz, $CDCl_3$) of **2k**

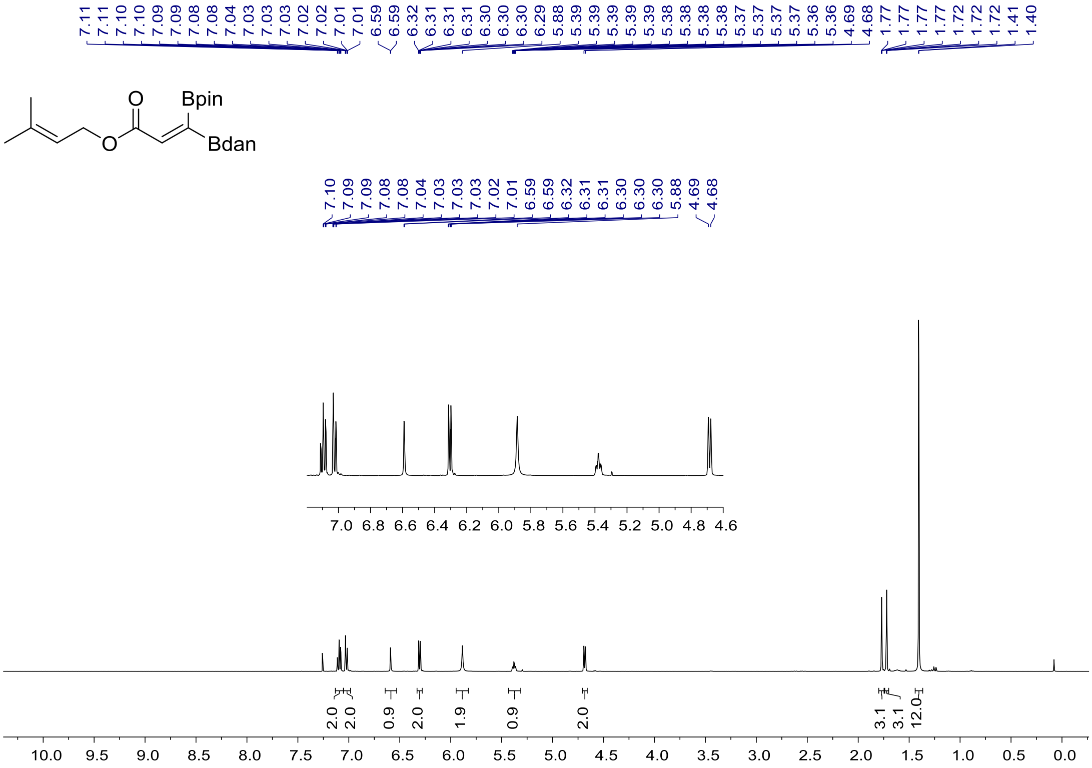

^{13}C{^{1}H} NMR spectrum (126 MHz, $CDCl_3$) of **2k**

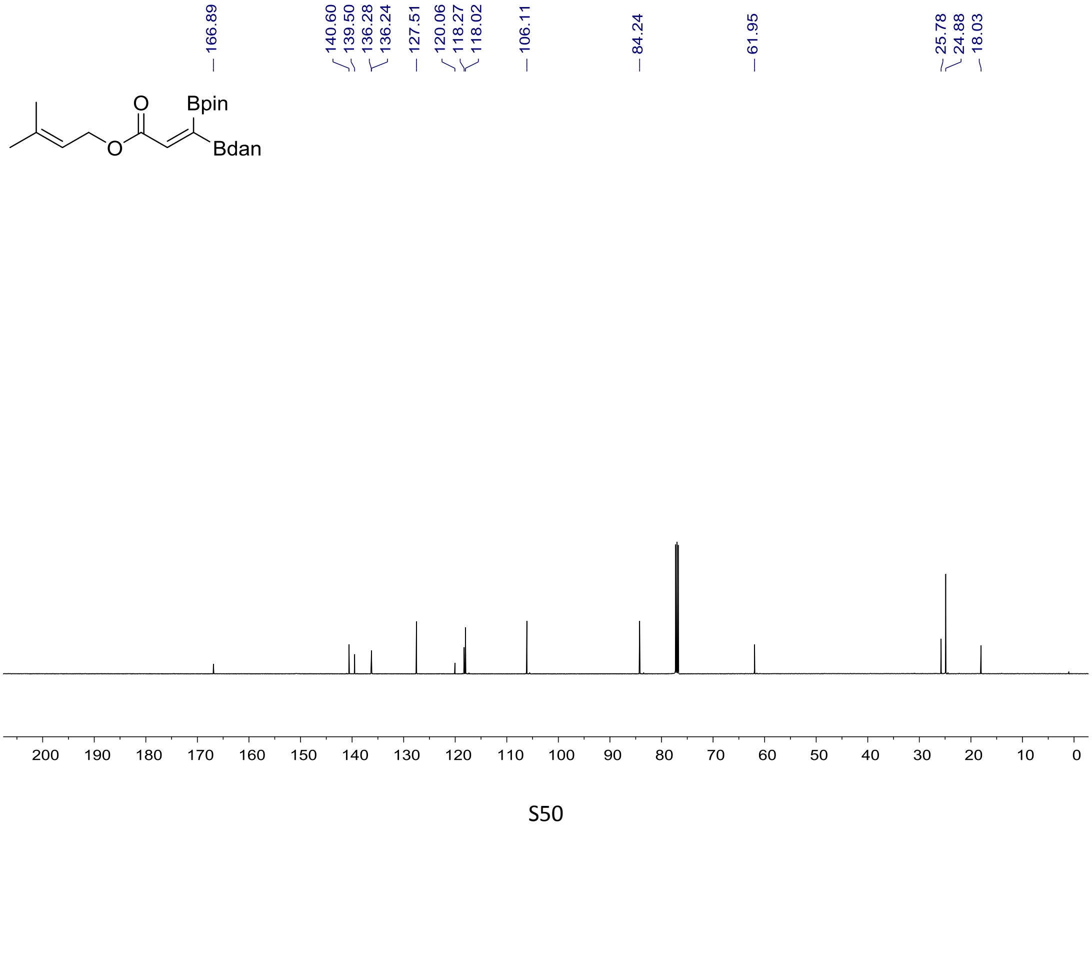

^{11}B NMR spectrum (160 MHz, $CDCl_3$) of **2k**

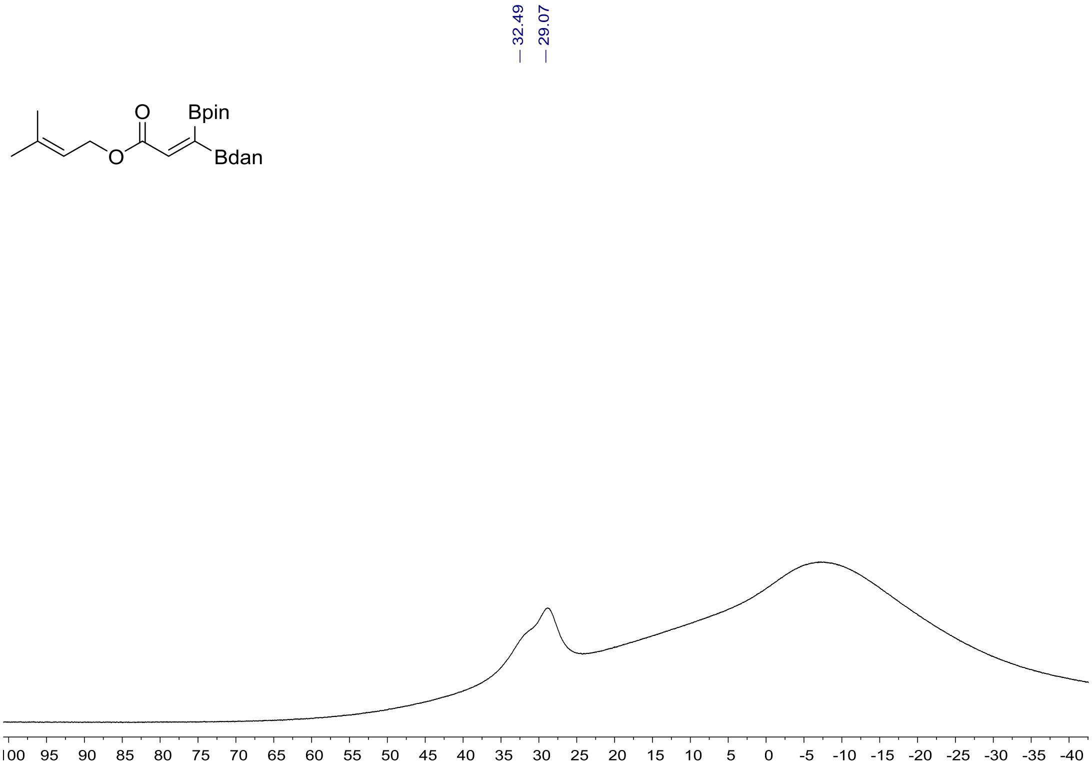

^{1}H NMR spectrum (500 MHz, $CDCl_3$) of **2l**

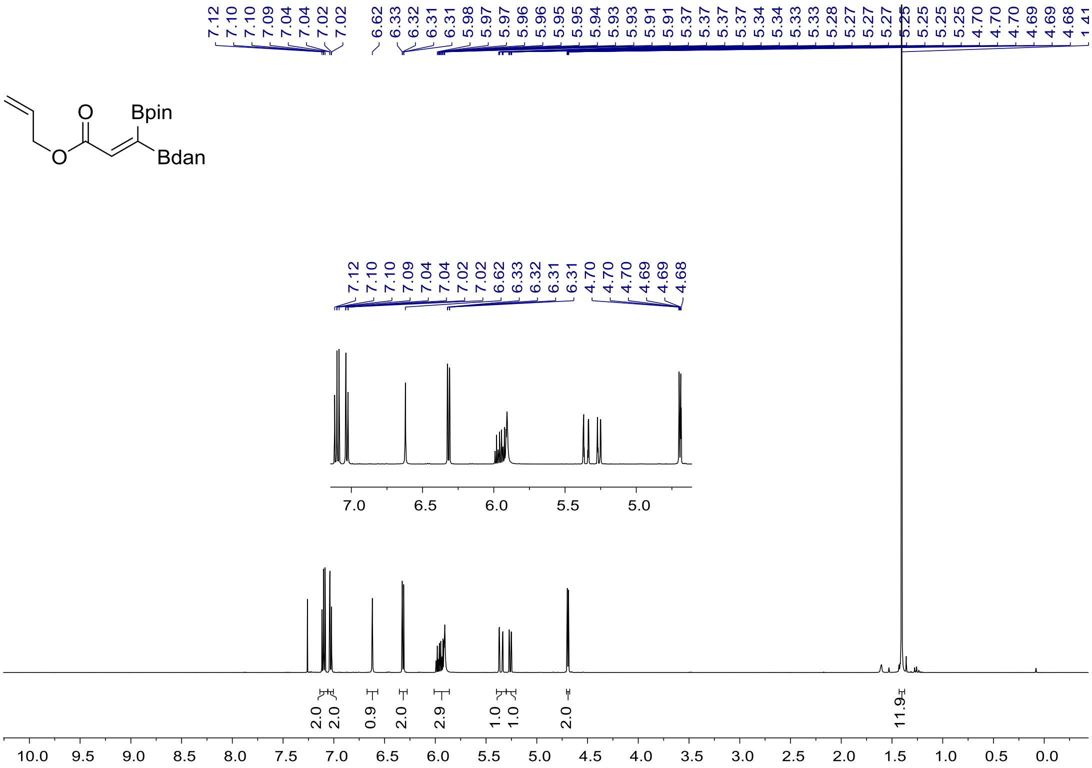

$^{13}C\{^{1}H\}$ NMR spectrum (126 MHz, $CDCl_3$) of **2l**

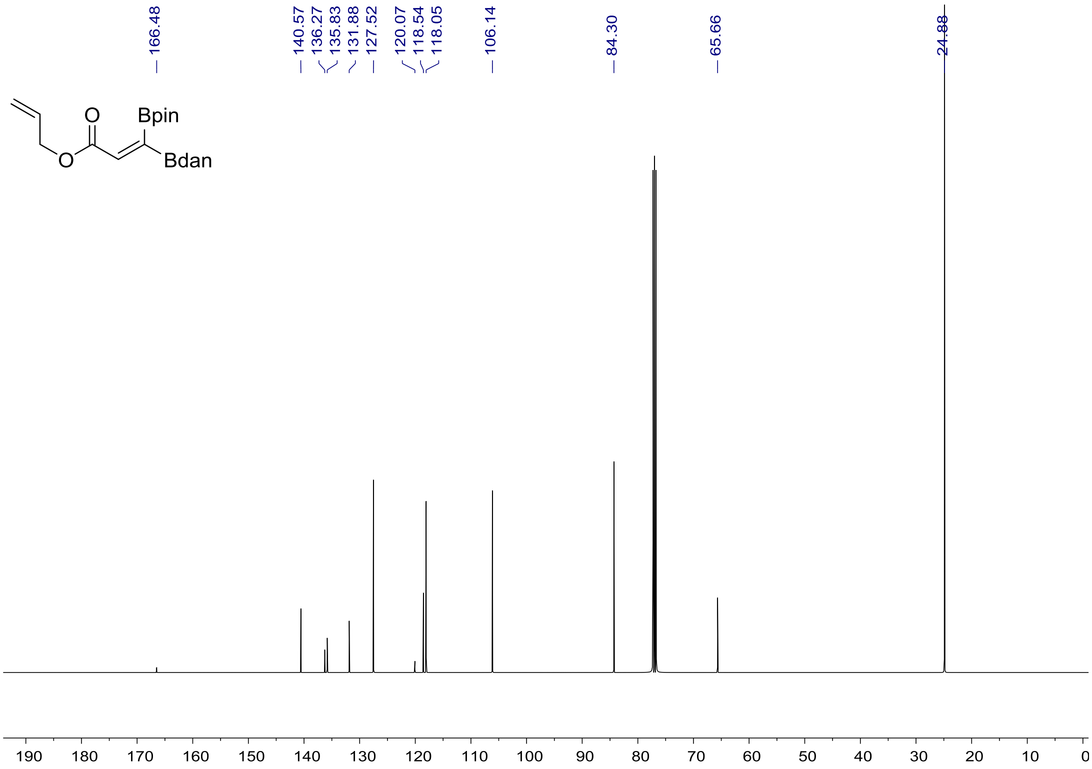

^{11}B NMR spectrum (160 MHz, $CDCl_3$) of **2l**

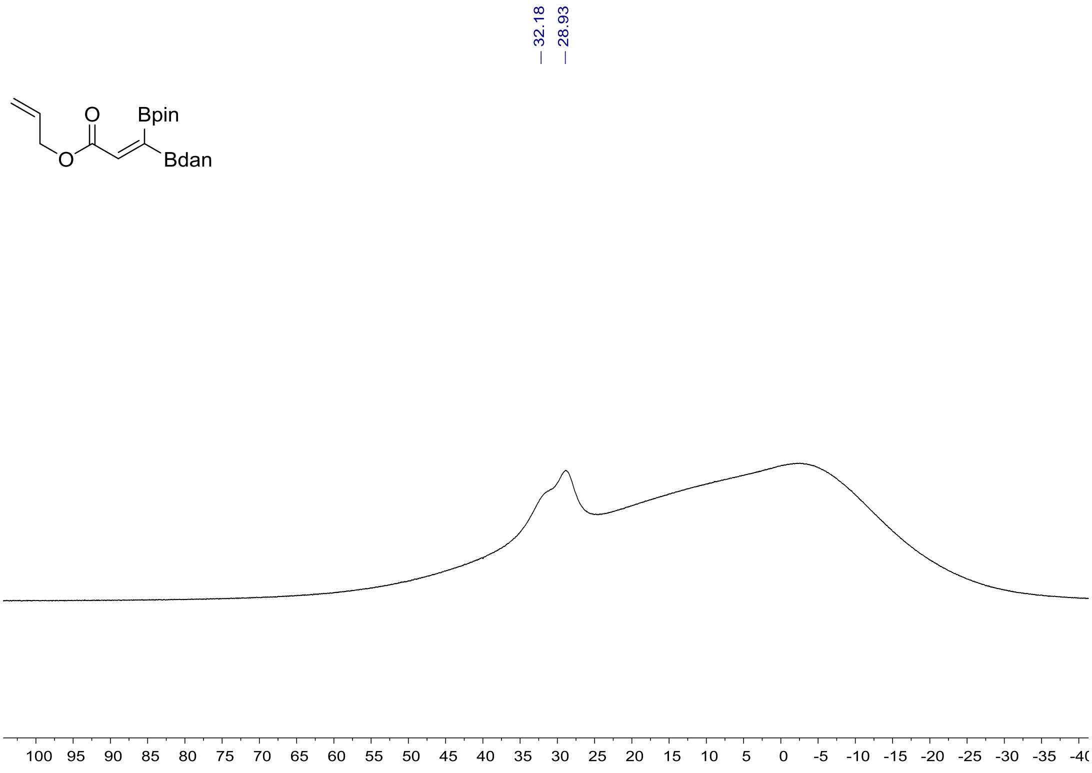

^{1}H NMR spectrum (500 MHz, $CDCl_3$) of **2m**

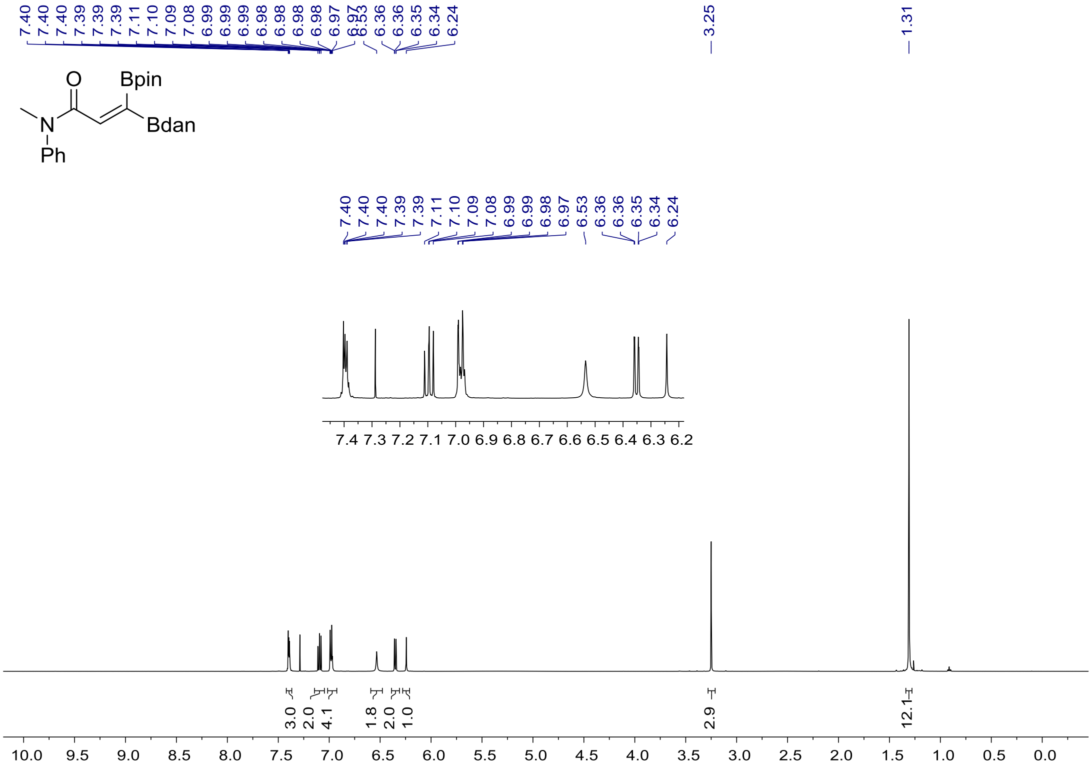

$^{13}C\{^{1}H\}$ NMR spectrum (126 MHz, $CDCl_3$) of **2m**

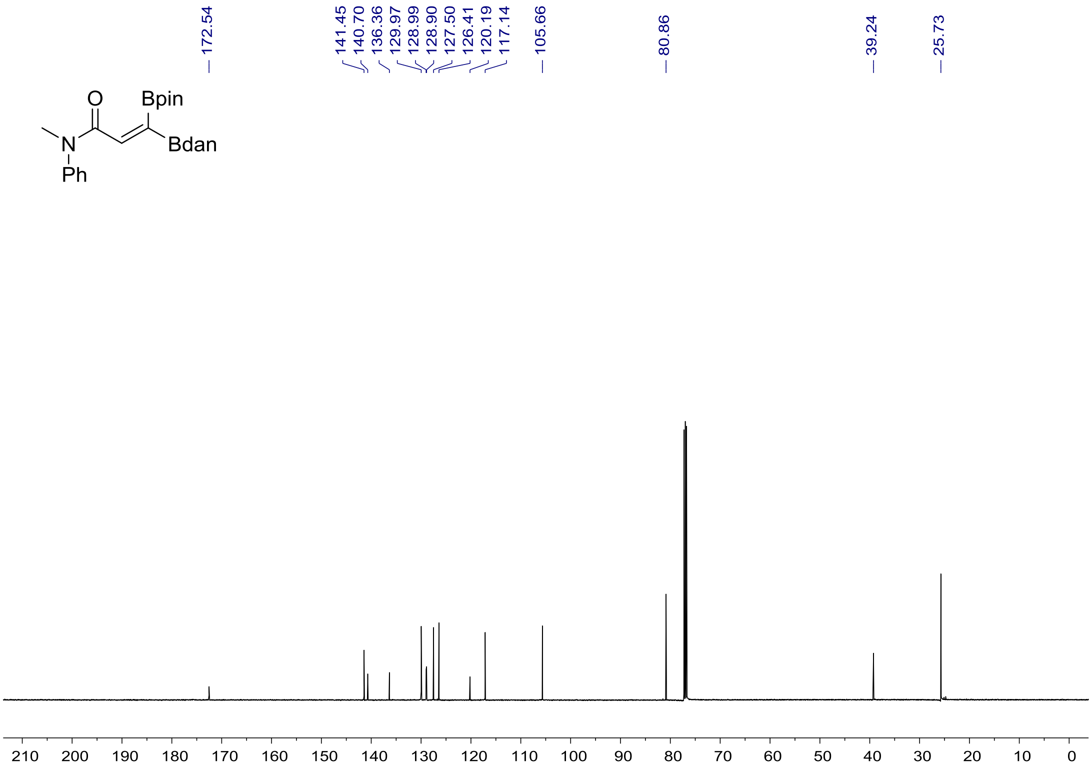

^{11}B NMR spectrum (160 MHz, $CDCl_3$) of **2m**

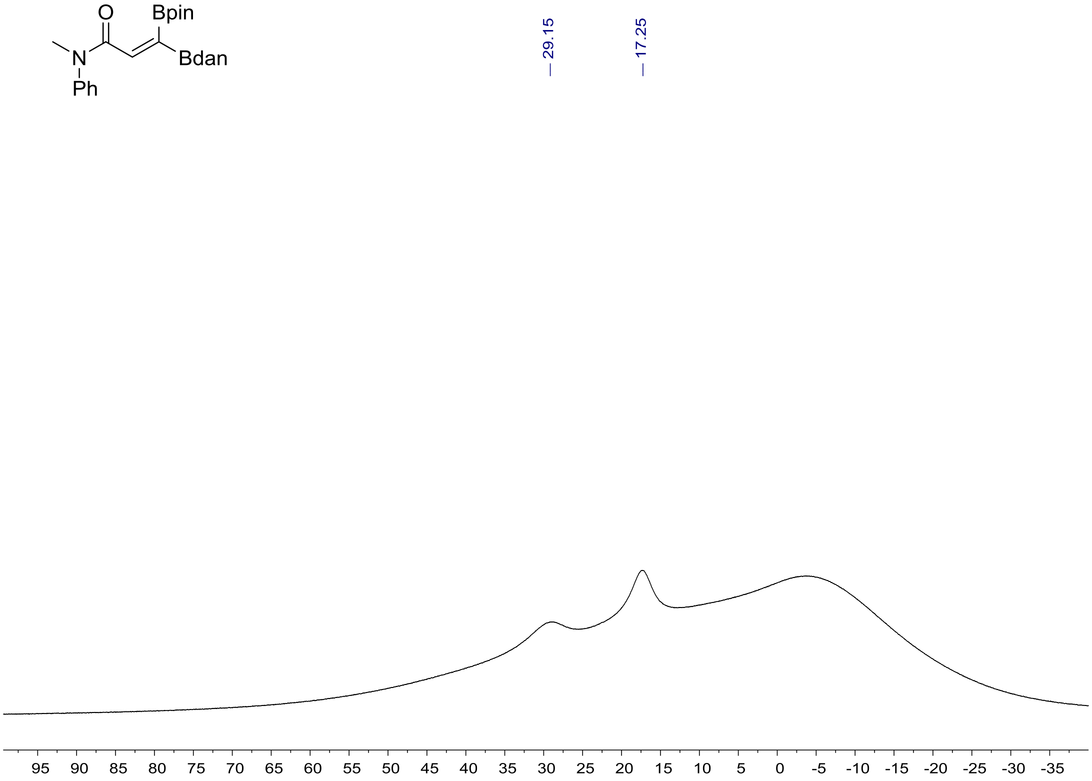

^{1}H NMR spectrum (500 MHz, $CDCl_3$) of **2n**

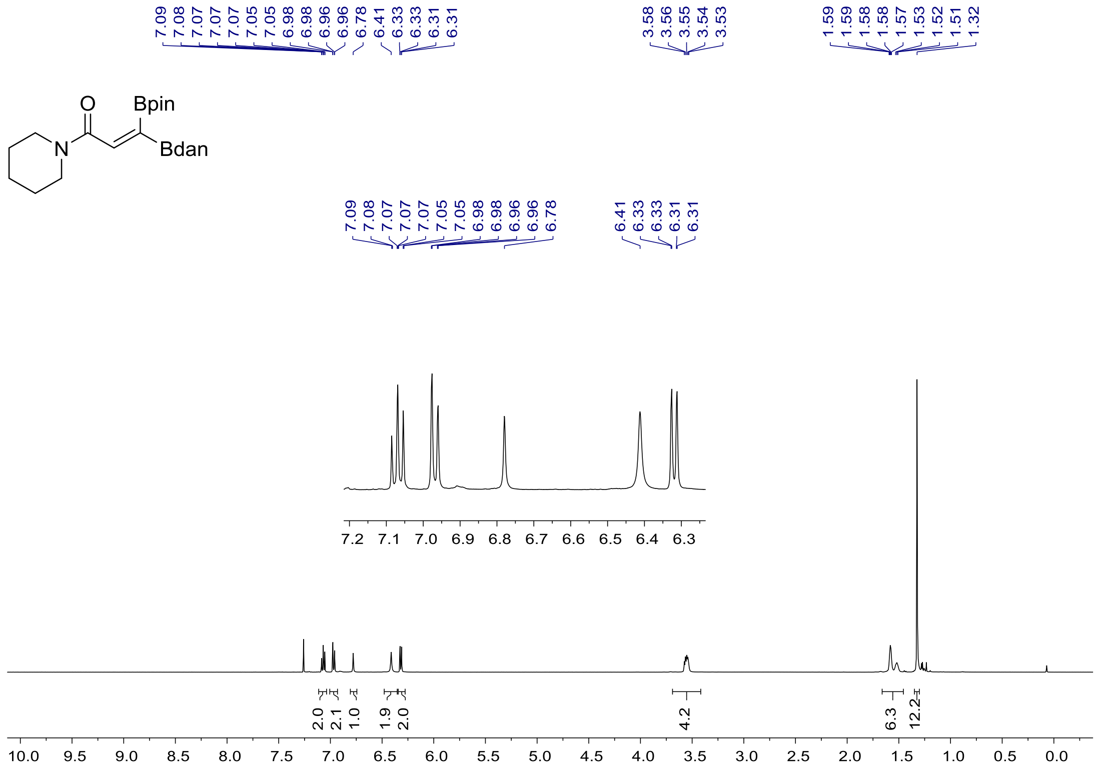

$^{13}C\{^{1}H\}$ NMR spectrum (126 MHz, $CDCl_3$) of **2n**

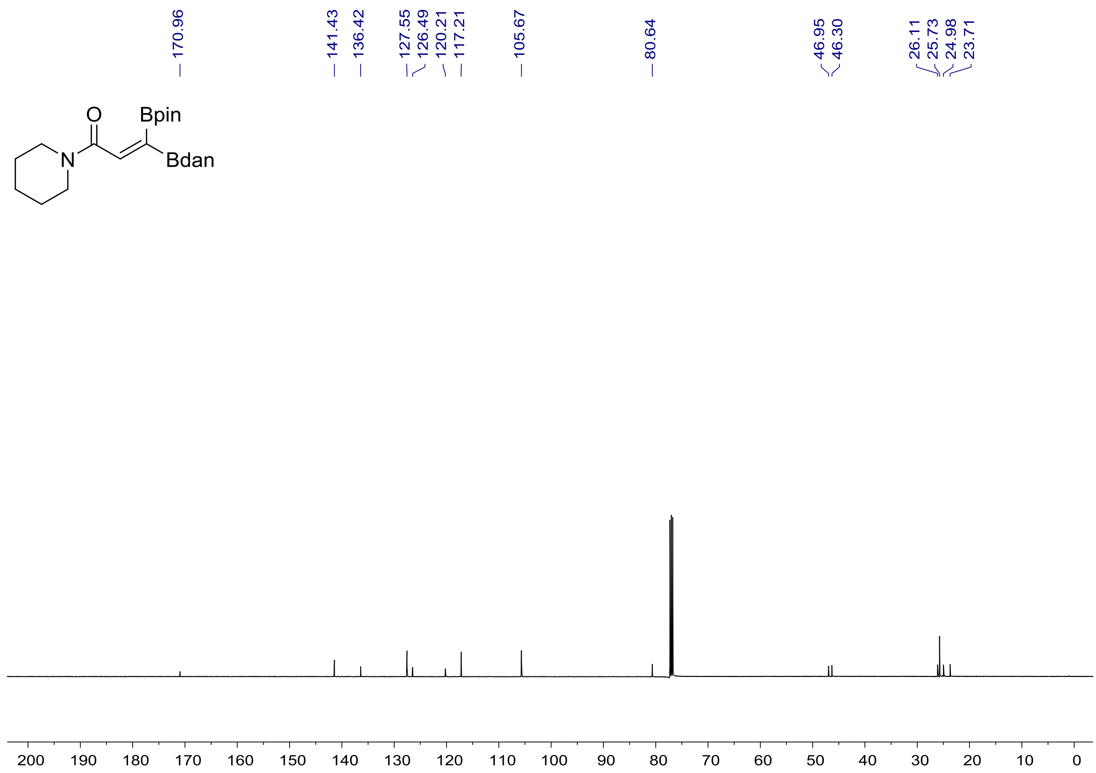

^{11}B NMR spectrum (160 MHz, $CDCl_3$) of **2n**

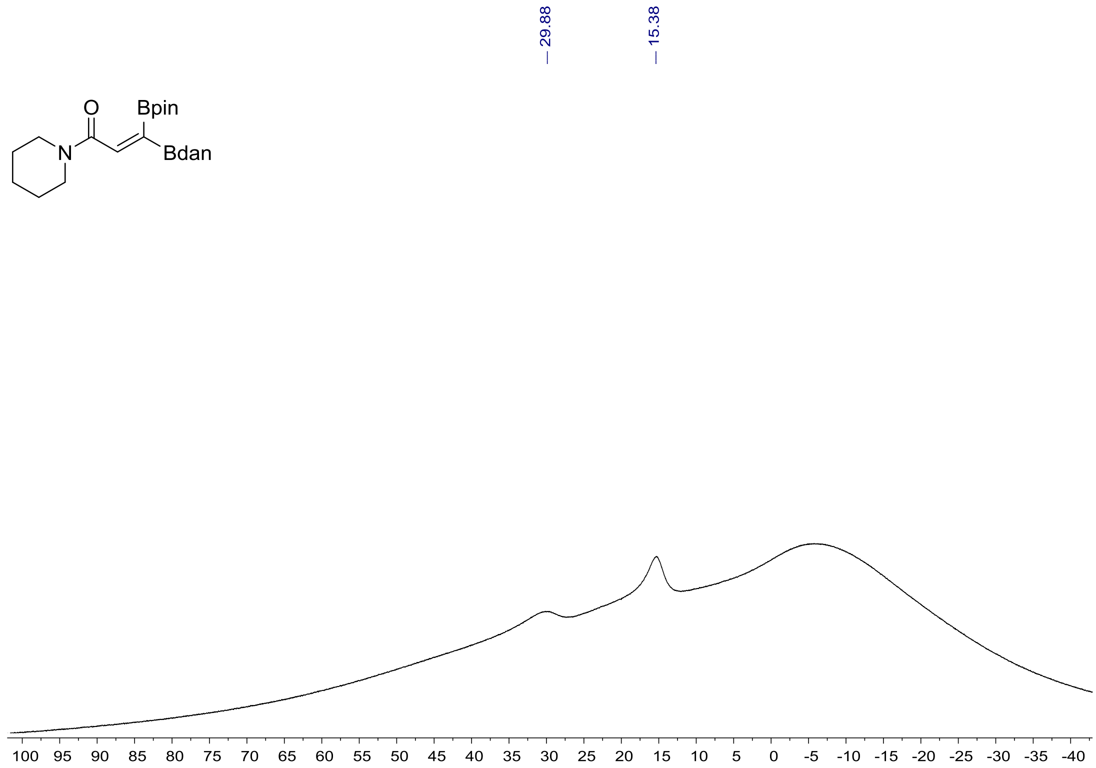

^{1}H NMR spectrum (300 MHz, $CDCl_3$) of **25a**

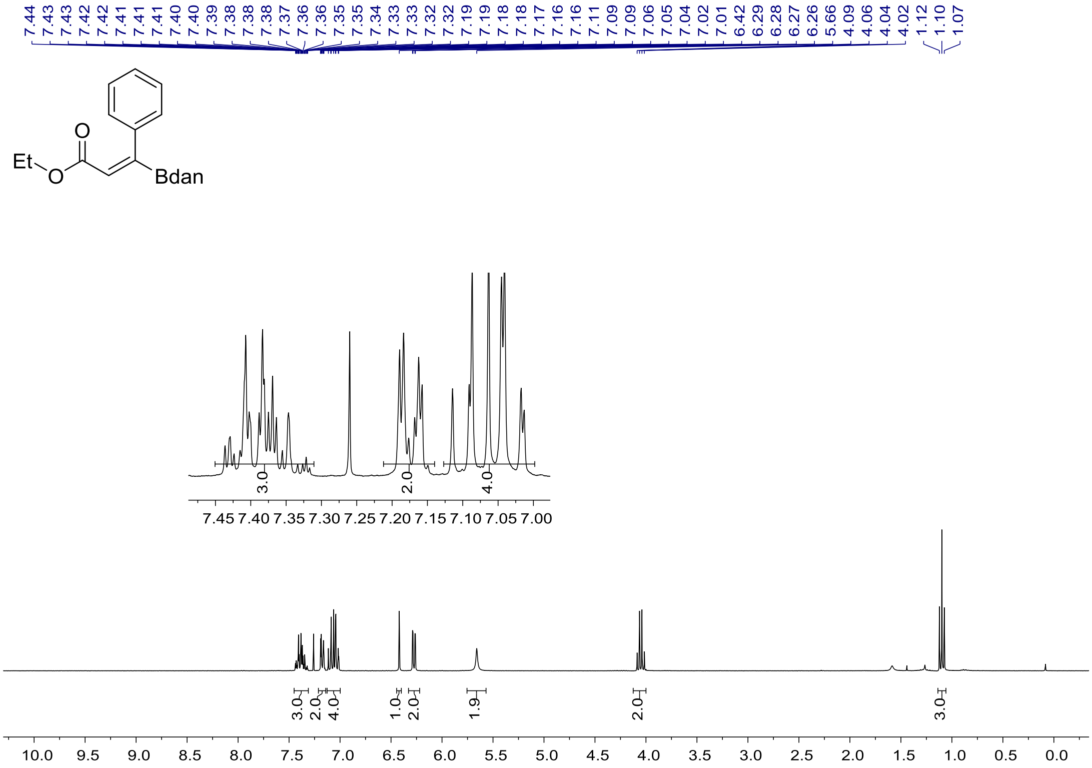

$^{13}C\{^1H\}$ NMR spectrum (75 MHz, $CDCl_3$) of **25a**

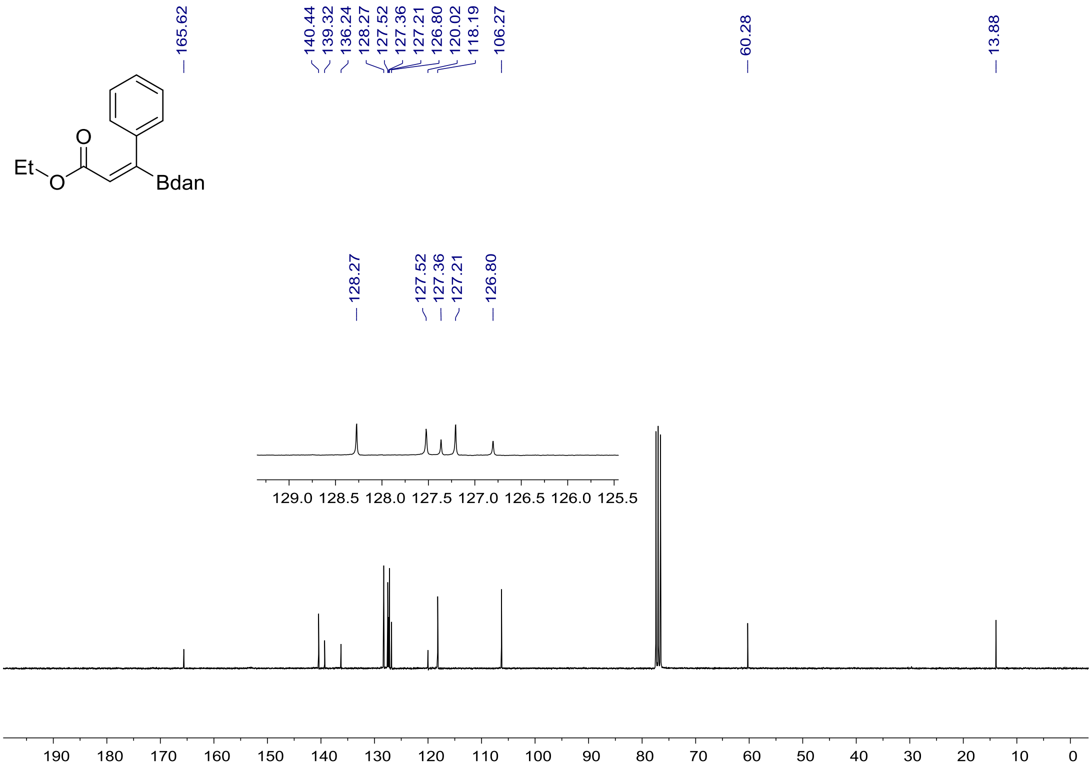

^{11}B NMR spectrum (96 MHz, CDCl$_3$) of **28a**

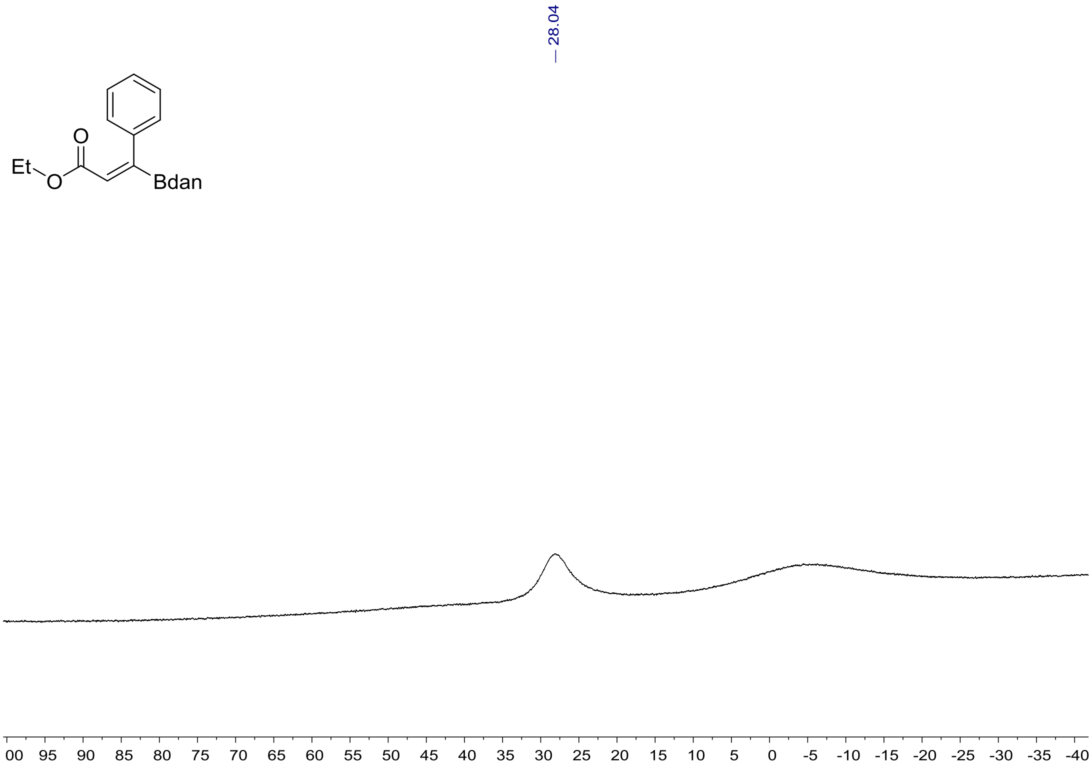

^{1}H NMR spectrum (300 MHz, CDCl$_3$) of **28b**

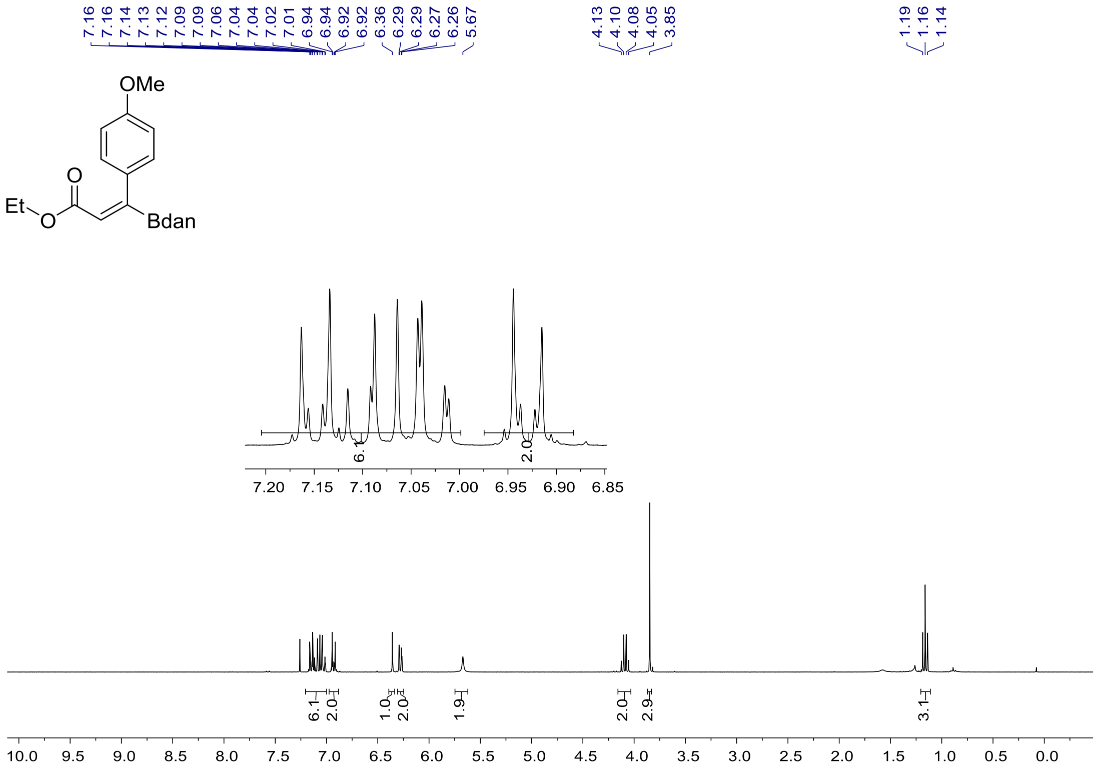

$^{13}C\{^1H\}$ NMR spectrum (75 MHz, $CDCl_3$) of **28b**

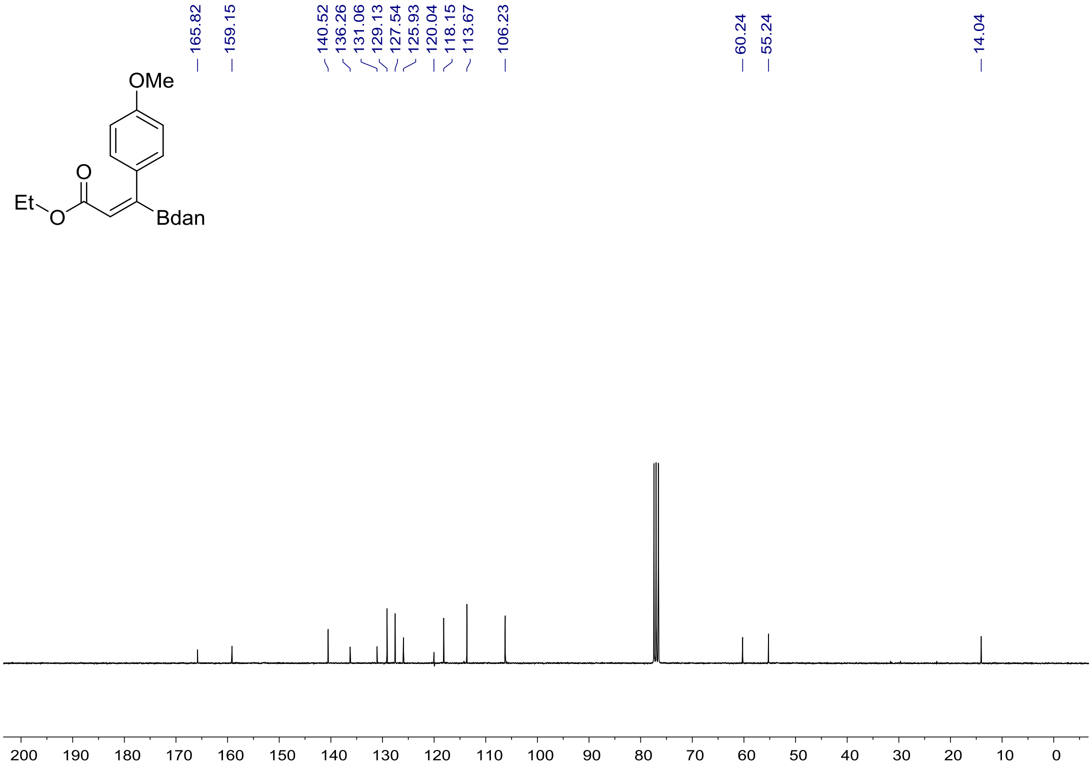

^{11}B NMR spectrum (96 MHz, $CDCl_3$) of **28b**

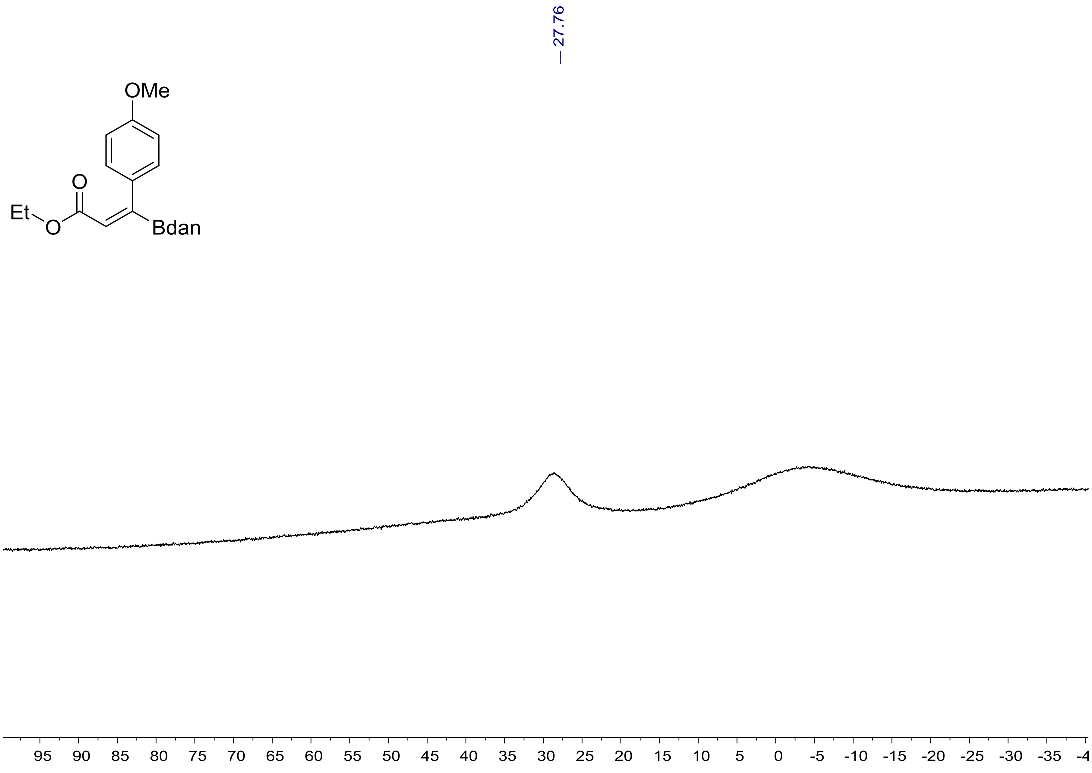

^{1}H NMR spectrum (300 MHz, $CDCl_3$) of **28c**

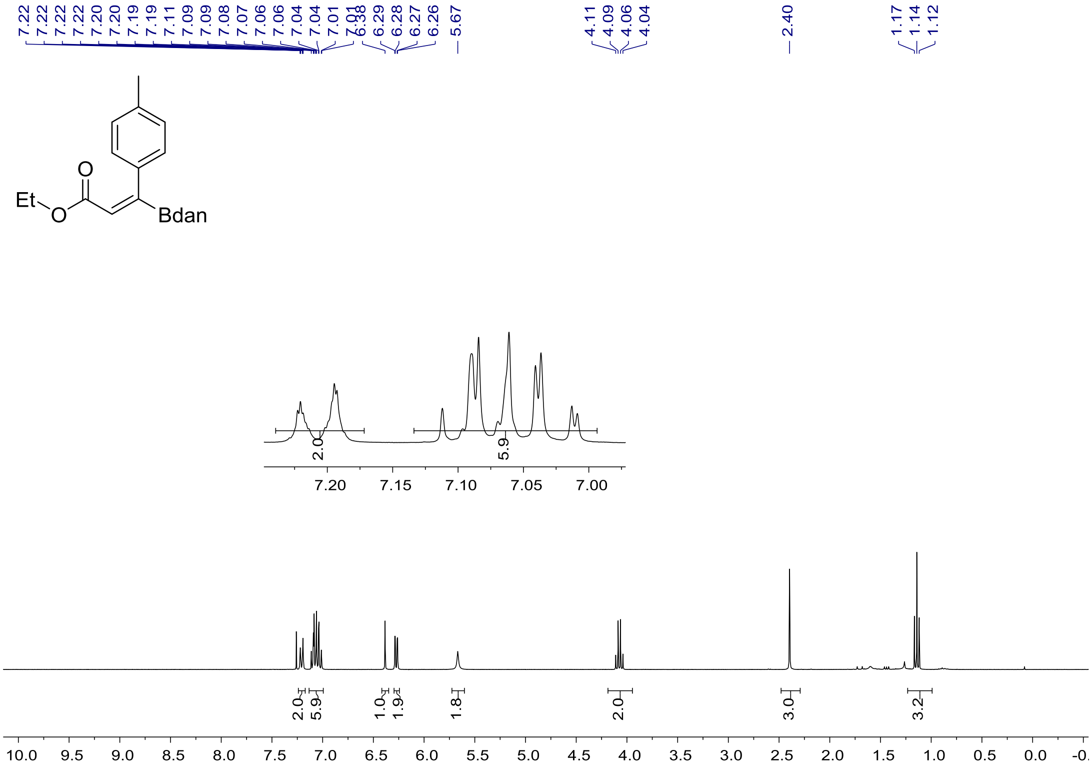

$^{13}C\{^{1}H\}$ NMR spectrum (75 MHz, $CDCl_3$) of **28c**

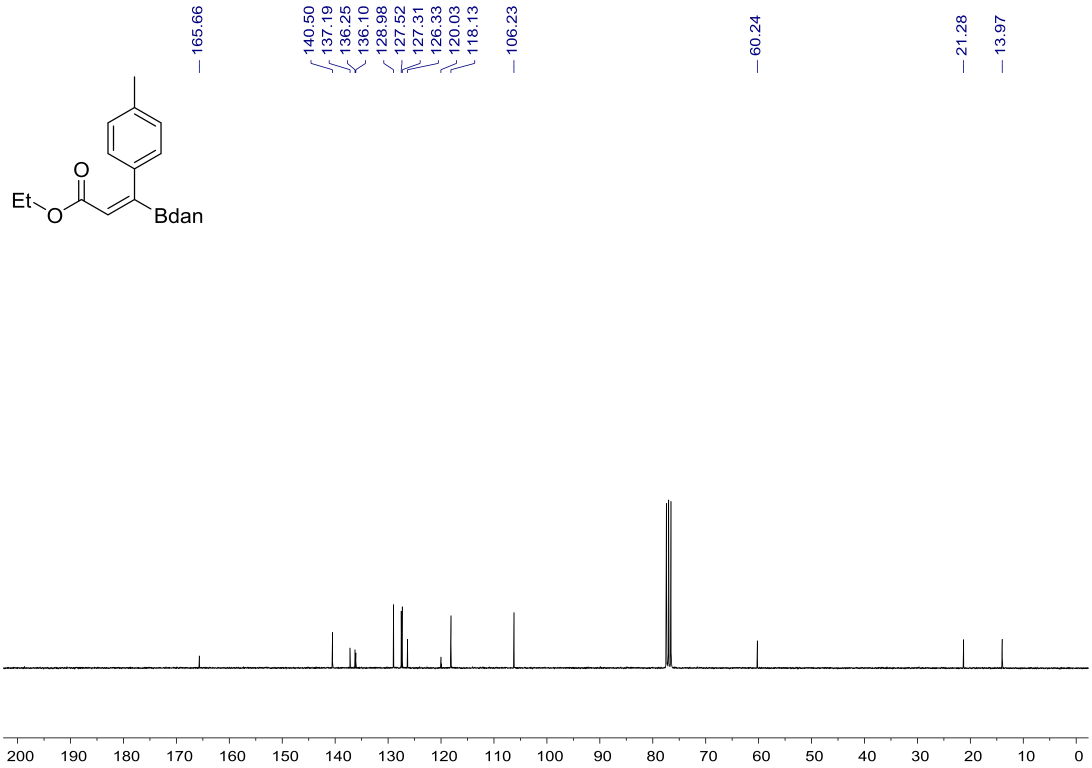

^{11}B NMR spectrum (96 MHz, $CDCl_3$) of **28c**

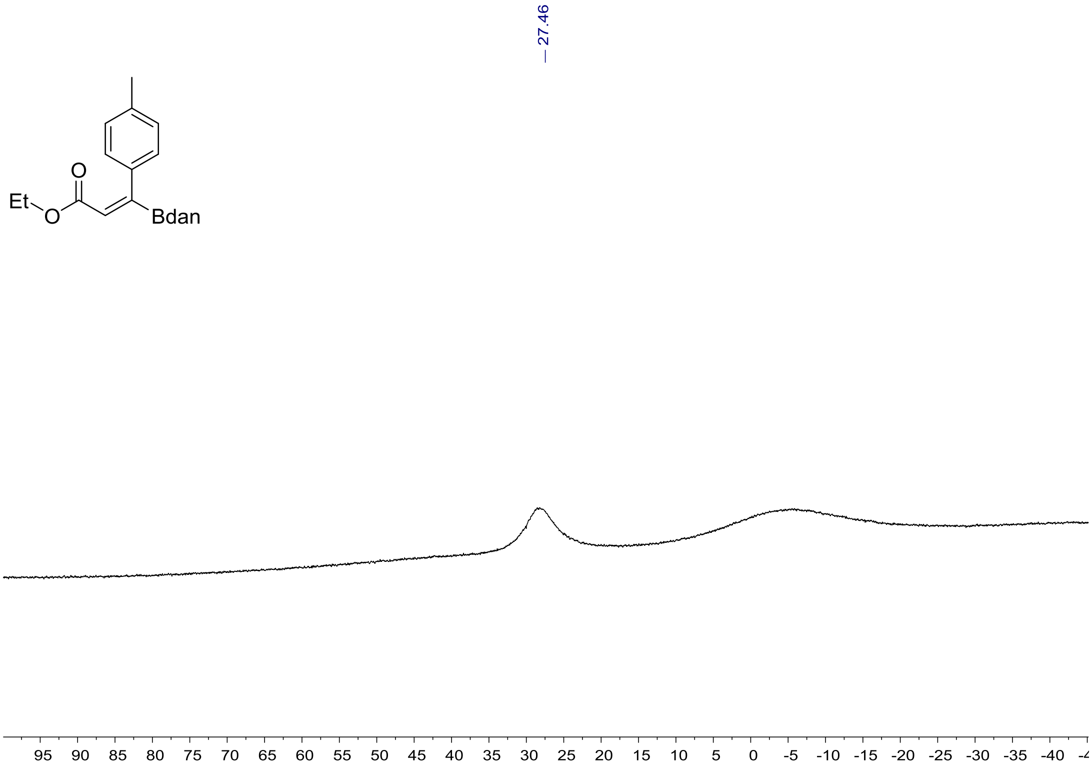

^{1}H NMR spectrum (300 MHz, $CDCl_3$) of **28d**

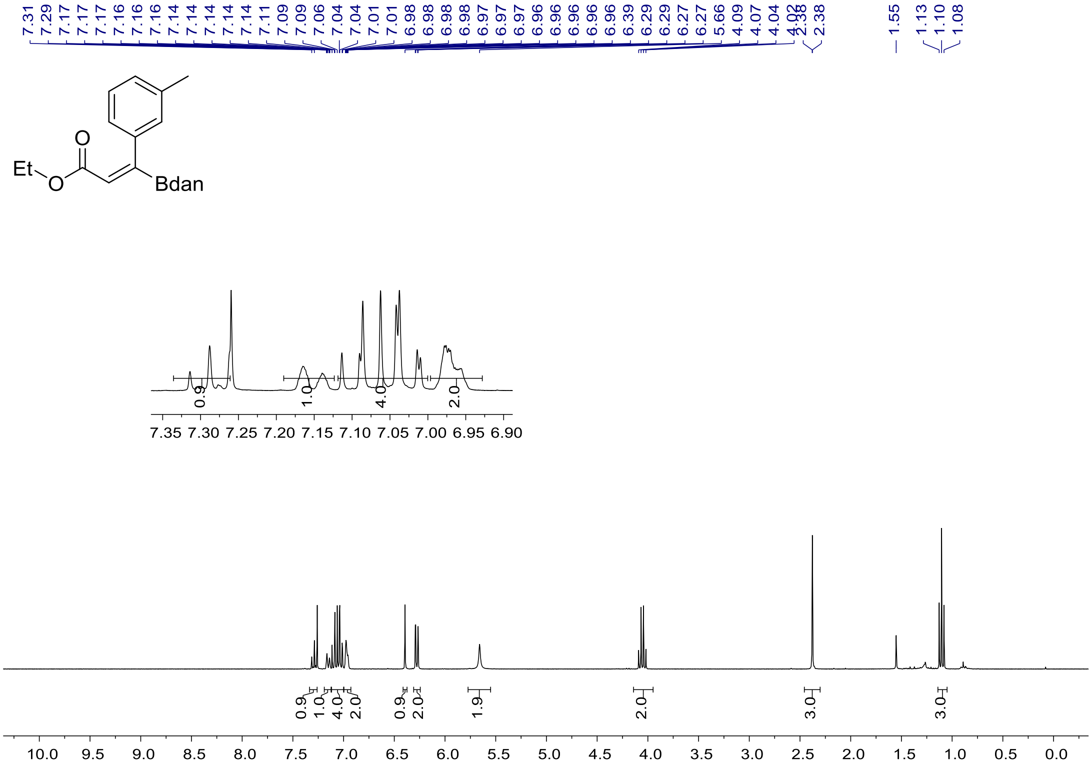

$^{13}C\{^{1}H\}$ NMR spectrum (75 MHz, $CDCl_3$) of **28d**

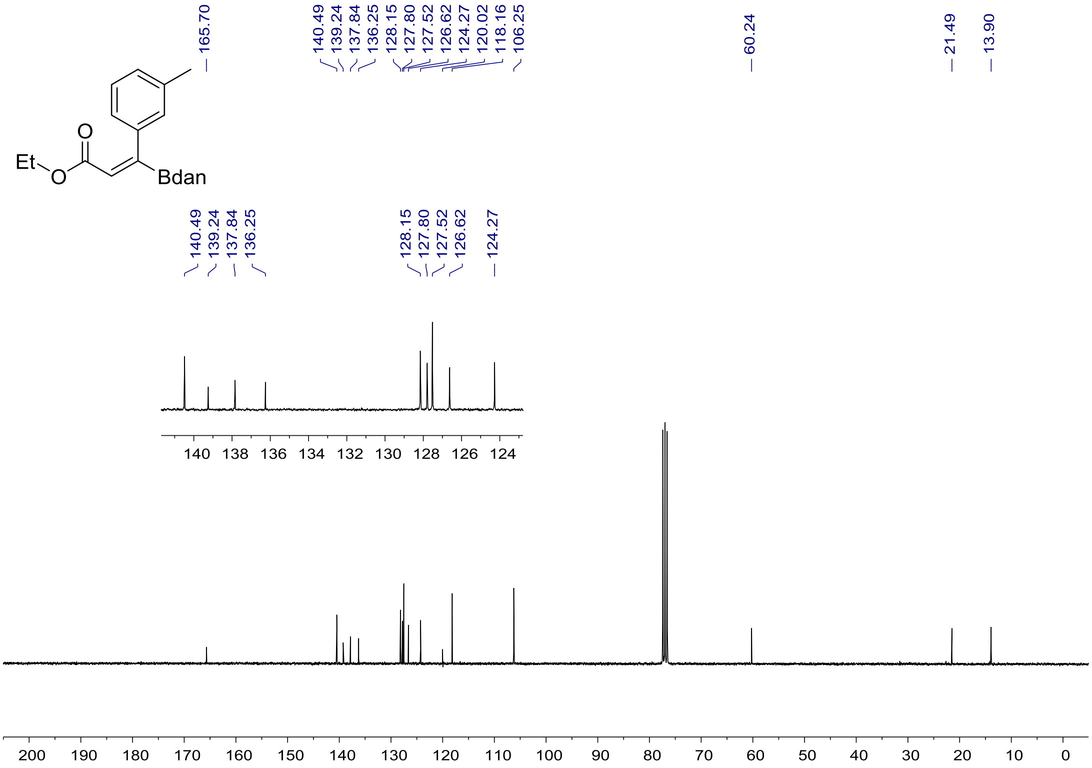

^{11}B NMR spectrum (96 MHz, $CDCl_3$) of **28d**

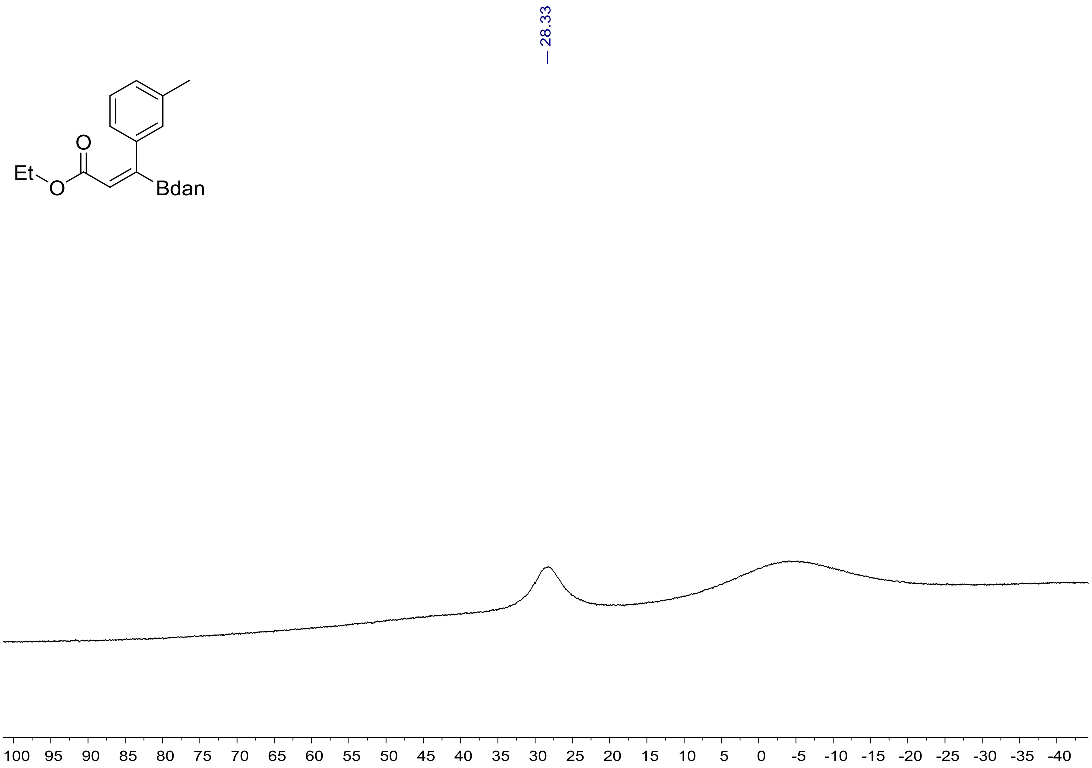

^{1}H NMR spectrum (300 MHz, CDCl$_3$) of **28e**

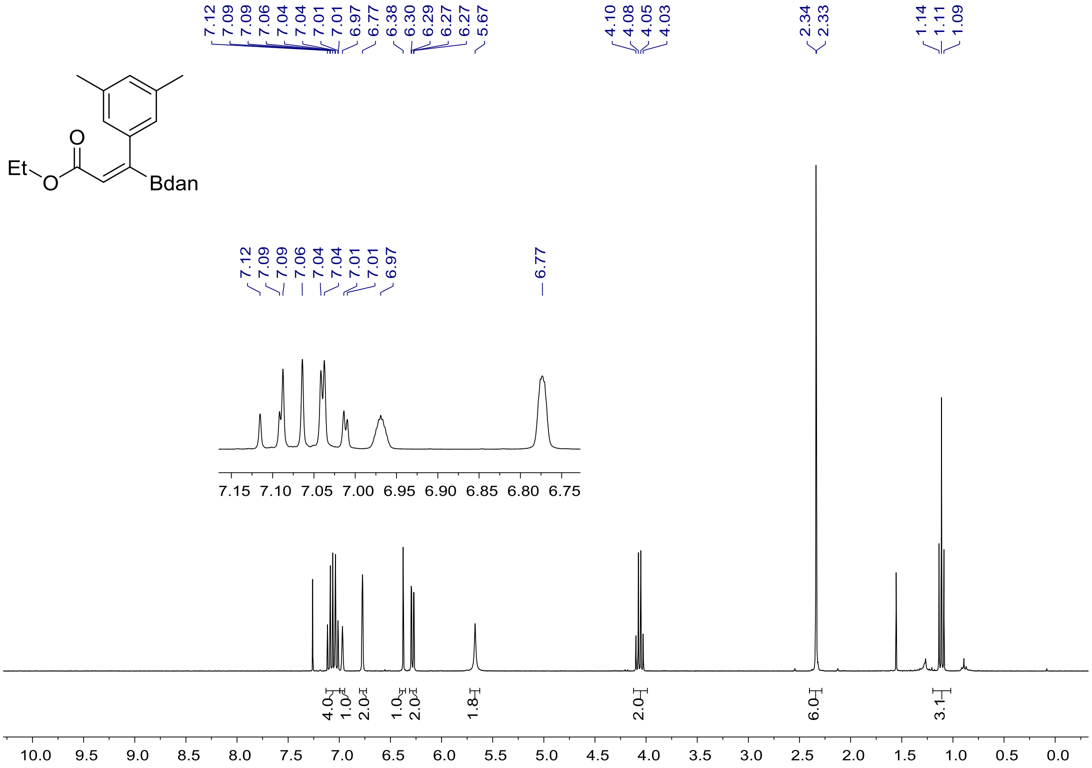

^{13}C{^{1}H} NMR spectrum (75 MHz, CDCl$_3$) of **28e**

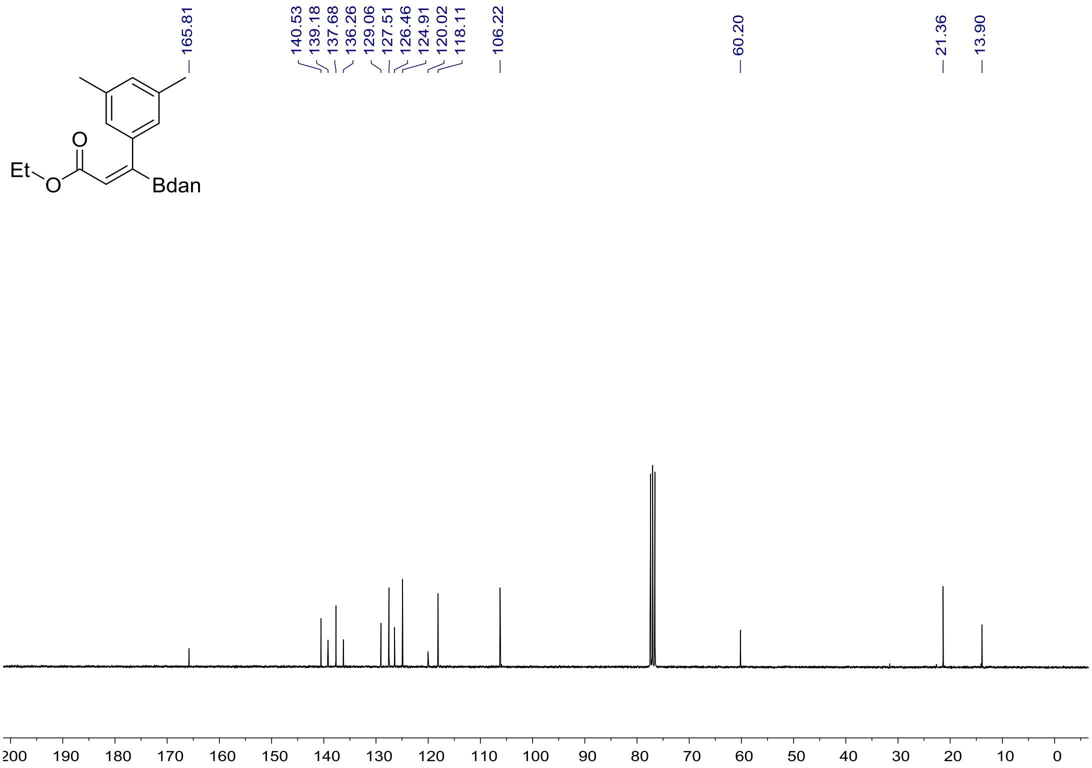

^{11}B NMR spectrum (96 MHz, $CDCl_3$) of **28e**

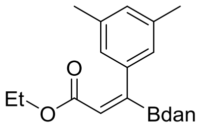

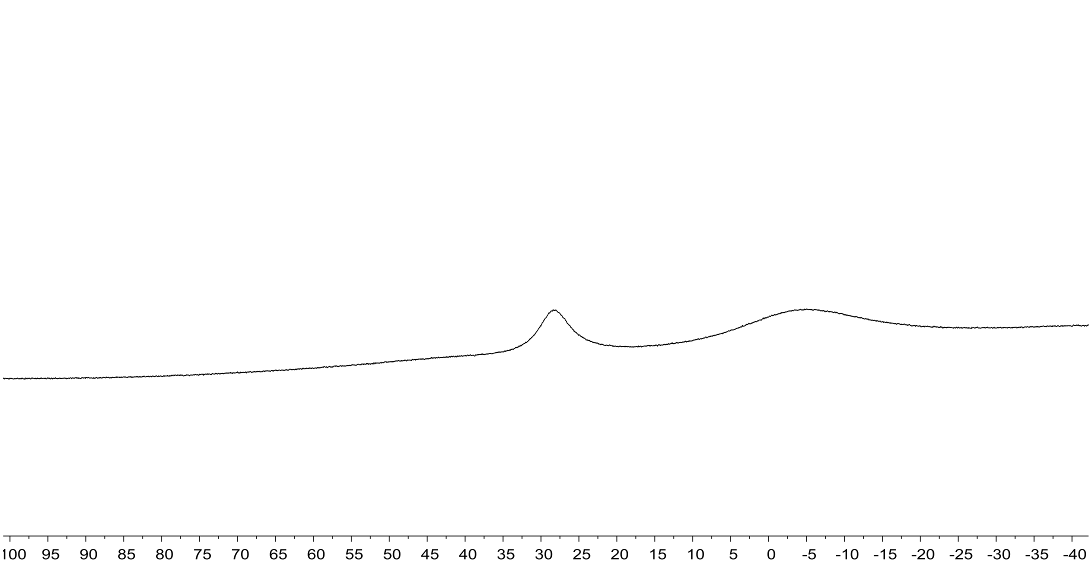

VIII. Cartesian Coordinates for All Optimized Geometries

1a

C	-0.79047100	-0.68828100	-0.00021500
O	-1.40303900	-1.74491800	0.00010100
O	0.54567500	-0.68166400	-0.00010000
C	1.28606500	0.58243100	-0.00006800
H	1.00337200	1.14697600	0.89251900
H	1.00353800	1.14691800	-0.89274400
C	2.75861800	0.23678200	0.00008100
H	3.02863900	-0.33960000	-0.89166200
H	3.34083400	1.16559300	0.00011500
H	3.02846800	-0.33955800	0.89190100
C	-1.47376200	0.58957500	-0.00002200
C	-2.09688900	1.62700500	0.00013000
H	-2.64730200	2.54724600	0.00043300

2a

C	2.86904700	1.95681400	-0.28522700
O	3.61151200	0.98169400	-0.28285400
C	1.39171900	1.88453500	-0.21272500
C	0.75702100	0.70488300	-0.06106000
O	3.30511800	3.22434900	-0.36198900
C	-5.20054500	2.55164200	1.02296400
C	-5.84083900	1.44074400	0.50780500
C	-5.08533300	0.36465000	-0.03727100
C	-3.65611000	0.45713700	-0.04127100
C	-3.02161800	1.62202300	0.49746200
C	-3.79266500	2.65254200	1.02285200
H	-6.78288500	-0.87249500	-0.57591400
H	-5.78518700	3.36892800	1.43831000
H	-6.92556400	1.37207800	0.51181500
C	-5.69835300	-0.80074500	-0.57786100
C	-2.88120000	-0.61797900	-0.58157900
H	-3.30278200	3.53287300	1.43144400
C	-3.51456800	-1.74028300	-1.10250700
C	-4.92365200	-1.82118200	-1.09572200
H	-2.91664700	-2.55105600	-1.51088600
H	-5.40075800	-2.70742500	-1.50701000
N	-1.49291100	-0.50014500	-0.56099700
N	-1.62892300	1.68007100	0.47798300
H	-0.98516200	-1.27708700	-0.97050400
H	-1.22115400	2.51238700	0.89062600
B	-0.80948500	0.63999600	-0.04601300
B	1.51099700	-0.66441300	0.08075200
O	1.61148400	-1.55428300	-0.96129900
O	1.88944200	-1.18916000	1.28908300
C	1.93802100	-2.86539900	-0.38584800
C	2.53132400	-2.48111700	1.02010700
C	2.91093500	-3.57676900	-1.31564100
H	3.79512300	-2.96785500	-1.52058200
H	3.23404100	-4.52536700	-0.87220000
H	2.41559600	-3.79763100	-2.26774700
C	0.62126500	-3.64035400	-0.30175100
H	0.78559400	-4.65864400	0.06520600
H	-0.09929700	-3.14575600	0.35781400
H	0.18216700	-3.70494500	-1.30331700
C	2.17096500	-3.44211300	2.14506000
H	2.56439900	-4.44222200	1.93048200
H	2.61846600	-3.09456700	3.08284400
H	1.08968300	-3.51532200	2.28739200
C	4.04024100	-2.23147200	0.99894200
H	4.59051800	-3.16765000	0.85812200
H	4.32016300	-1.53084000	0.20805000
H	4.34090600	-1.79867800	1.95942800
H	0.86227100	2.83089200	-0.30824300
C	4.74352600	3.42533300	-0.44830300
C	4.99228000	4.91535300	-0.54349300
H	5.11230700	2.89148000	-1.32933200
H	5.20509300	2.98916400	0.44273300
H	6.07161600	5.09606800	-0.60936000
H	4.51772900	5.33881200	-1.43602000
H	4.60982100	5.43720000	0.34113500
H	-3.39531000	3.27032400	1.78443400
C	-3.54489600	-1.67982700	-1.33748800

C	-4.95361100	-1.76559000	-1.35958100
H	-2.94359500	-2.43843700	-1.83343600
H	-5.42191700	-2.59869600	-1.87810700
N	-1.52964200	-0.50659600	-0.62680000
N	-1.68996800	1.54838900	0.63746400
H	-0.99930800	-1.21691000	-1.11875900
H	-1.29121000	2.32231400	1.15392000
B	-0.85700800	0.57923000	0.00531700
B	1.51628800	-0.67404300	0.11893500
O	1.51875200	-1.63038000	-0.87151600
O	2.07496000	-1.10679200	1.29195500
C	2.04081600	-2.87119500	-0.29809600
C	2.76035700	-2.36772700	1.02067400
C	2.96186400	-3.52283900	-1.32833900
H	3.75697500	-2.84468600	-1.64657900
H	3.41838100	-4.43310400	-0.92115200
H	2.38137300	-3.80251200	-2.21422400
C	0.82811400	-3.77407500	-0.03379500
H	1.13115800	-4.75018100	0.36075600
H	0.12869400	-3.31799000	0.67451500
H	0.29638900	-3.94227800	-0.97689400
C	2.58466900	-3.27603500	2.23737400
H	3.03529200	-4.25952400	2.05702700
H	3.08660300	-2.82744800	3.10111000
H	1.53207700	-3.41601500	2.49573800
C	4.24526300	-2.03336700	0.82610300
H	4.83840100	-2.93874700	0.65404300
H	4.39729500	-1.33961000	-0.00439900
H	4.61688800	-1.54720900	1.73421000
H	0.74696900	2.79439100	-0.32303700
C	4.57673800	3.55090200	-0.56220200
C	4.77004800	5.05544400	-0.60940800
H	4.92352300	3.06099900	-1.47793900
H	5.10163600	3.09397300	0.28314600
H	5.83600500	5.28622000	-0.72114800
H	4.23237000	5.49507600	-1.45642000
H	4.41083200	5.52760400	0.31140700

2a′

C	0.76717700	3.22282300	-0.22902800
O	-0.43180300	3.47750000	-0.17140800
C	1.42996800	1.89468800	-0.16307100
C	0.90149100	0.64441600	-0.08817800
O	1.70234000	4.17911700	-0.36628100
C	-5.45376600	1.00727700	0.02253700
C	-5.71777300	-0.35005600	0.02883400
C	-4.64800000	-1.28890400	0.01331600
C	-3.30288900	-0.79803100	-0.00828500
C	-3.05899700	0.61147500	-0.01567000
C	-4.13158400	1.49892000	0.00031900
H	-5.88117300	-3.07345200	0.03401100
H	-6.27755200	1.71715100	0.03443500
H	-6.74066400	-0.71742400	0.04530600
C	-4.86184500	-2.69638000	0.01785100
C	-2.21026000	-1.72072900	-0.02399000
H	-3.94033600	2.56900900	-0.00524400
C	-2.45823100	-3.09034800	-0.01929400
C	-3.78655500	-3.56551300	0.00156500
H	-1.62251700	-3.78560300	-0.03174500
H	-3.96009900	-4.63904000	0.00469700
N	-0.91818500	-1.20936400	-0.04313700
N	-1.74177400	1.05439600	-0.04064800
H	-0.17002100	-1.89643300	-0.06120300
H	-1.59444200	2.06128100	-0.05678200
B	-0.61280500	0.18758700	-0.05906200
B	2.02423500	-0.46308400	-0.03223800
O	1.79069100	-1.80974800	-0.19120800
O	3.34879800	-0.18005900	0.18104800
C	3.02805400	-2.52127800	0.15899200
C	4.11140000	-1.40827200	-0.06750400
C	3.16114400	-3.74117700	-0.74045500
H	3.09624200	-3.47538900	-1.79856100
H	4.12213400	-4.23656300	-0.56236600
H	2.36254200	-4.45606700	-0.51396500
C	2.88326300	-2.94561900	1.62106700
H	3.74983000	-3.53000500	1.94680800

H	2.77656200	-2.08037800	2.28382900
H	1.98952300	-3.57002400	1.72462200
C	5.28706700	-1.45757400	0.89787300
H	5.82209100	-2.40792200	0.79153000
H	5.98607400	-0.64569200	0.66895700
H	4.96496200	-1.35202500	1.93693800
C	4.60885900	-1.32944800	-1.51190500
H	5.23863300	-2.19065200	-1.75761800
H	3.77771200	-1.29045500	-2.22398500
H	5.20752700	-0.42040500	-1.63272400
H	2.51260300	1.99749900	-0.18196500
C	1.25874000	5.56567000	-0.41660100
C	1.08425700	6.13349900	0.98064800
H	0.33527100	5.61753400	-0.99746000
H	2.05768400	6.07508600	-0.95868700
H	0.81727900	7.19463000	0.90474900
H	0.28761400	5.61700200	1.52579200
H	2.01576800	6.05347000	1.55227200

^{t}BuO$^-$

C	-1.35557900	-0.51055900	-0.43493600
C	0.00001700	-0.00022600	0.13521200
H	-1.54728000	-1.53242800	-0.07844600
H	-2.17366400	0.12847800	-0.07380100
H	-1.39122600	-0.52104500	-1.53574000
C	0.23522500	1.42999600	-0.43262200
C	1.12074700	-0.91774600	-0.43482200
H	1.19924400	1.81795000	-0.07455500
H	0.23996400	1.46817700	-1.53338900
H	-0.55221600	2.10656800	-0.07174300
H	1.14880100	-0.94145600	-1.53562900
H	2.10083700	-0.57267000	-0.07640300
H	0.97655400	-1.94640400	-0.07551300
O	-0.00043500	-0.00199500	1.50727800

^{t}BuOH

C	0.66769900	1.26250500	-0.53406700
C	-0.00807300	-0.00002300	0.01203300
H	0.19252300	2.16105100	-0.12319700
H	1.73231700	1.27958300	-0.26596100
H	0.59449600	1.30116300	-1.62762700
C	0.67237600	-1.25978200	-0.53463600
C	-1.50139800	-0.00265100	-0.30777900
H	0.20047200	-2.16029000	-0.12429100
H	0.59944700	-1.29814800	-1.62822400
H	1.73701600	-1.27307700	-0.26639700
H	-1.65858500	-0.00252900	-1.39205100
H	-1.98454900	-0.89340300	0.11100400
H	-1.98777000	0.88603400	0.11166900
O	0.07203000	-0.00021900	1.46063100
H	1.01477100	0.00107300	1.70671800

BpinBdan

C	4.29728300	2.43214500	-0.08563900
C	5.00681200	1.24682400	-0.04387800
C	4.32136300	0.00001100	-0.00000200
C	2.88873800	0.00000000	0.00000100
C	2.18165500	1.24375500	-0.04632800
C	2.88616500	2.44205000	-0.08776600
H	6.09382100	-1.24911600	0.04344800
H	4.82953800	3.37979100	-0.11850800
H	6.09380000	1.24916800	-0.04346200
C	5.00683300	-1.24678900	0.04387000
C	2.18167500	-1.24376700	0.04633300
H	2.34024300	3.38169000	-0.12197500
C	2.88620600	-2.44205000	0.08776700
C	4.29732400	-2.43212200	0.08563400
H	2.34030000	-3.38170000	0.12197900
H	4.82959500	-3.37976000	0.11850000
N	0.78844200	-1.20534800	0.04775500
N	0.78842200	1.20531200	-0.04774400
H	0.33185800	-2.11045100	0.08208300
H	0.33182100	2.11040600	-0.08207200
B	0.02456400	-0.00002500	0.00000600
B	-1.66963800	-0.00002300	0.00000500
O	-2.43350000	-1.13591500	0.14167200
O	-2.43347500	1.13588800	-0.14166700
C	-3.83058500	-0.77515000	-0.12779400
C	-3.83056800	0.77515400	0.12779200

C	-4.73194700	-1.57604200	0.80209600
H	-4.44832800	-1.45207200	1.85039500
H	-5.77504900	-1.26168200	0.68268400
H	-4.66688000	-2.64090100	0.55218900
C	-4.10330700	-1.15363200	-1.58529400
H	-5.14814100	-0.96530000	-1.85352700
H	-3.46006200	-0.59622700	-2.27430600
H	-3.90290600	-2.22222500	-1.71862300
C	-4.73190800	1.57606600	-0.80210300
H	-5.77501900	1.26173300	-0.68269200
H	-4.66681400	2.64092400	-0.55219900
H	-4.44829000	1.45208600	-1.85040100
C	-4.10329000	1.15364200	1.58529000
H	-5.14813000	0.96533200	1.85351700
H	-3.46006100	0.59622400	2.27430600
H	-3.90286800	2.22223100	1.71862000

3

C	-0.90213500	-0.56952200	-0.00012000
O	-1.54211600	-1.62069100	0.00006500
O	0.45209200	-0.66126900	-0.00006700
C	1.26805700	0.53960200	0.00004600
H	1.02879200	1.13046600	0.88945700
H	1.02879500	1.13063400	-0.88925500
C	2.71858900	0.10164200	0.00000500
H	2.95140900	-0.49281000	-0.89093800
H	3.36322900	0.98871500	0.00009600
H	2.95140400	-0.49298800	0.89083000
C	-1.48993100	0.73501400	0.00005000
C	-2.02848500	1.85854000	0.00001100

4-ts

C	-1.12832500	2.75090300	0.28184500
O	-1.41586900	3.46939700	0.67480900
C	0.19076600	2.31678200	0.62277400
C	1.28231300	1.73361800	0.75496300
O	-2.06300700	2.25082900	1.12464400
C	-3.45140400	2.50588800	0.78863700
H	-3.62705000	2.17393500	0.23897100
H	-3.63359100	3.58432900	0.84104600
C	-4.30491000	1.73984700	1.77796900
H	-5.36381000	1.90773400	1.54764000
H	-4.10665600	0.66456500	1.71738100
H	-4.11879100	2.07638500	2.80450500
C	-3.61928700	-0.54131500	-2.75993500
C	-4.31501300	-1.13634200	-1.72327000
C	-3.64243900	-1.49360900	-0.52086000
C	-2.23908100	-1.22648600	-0.40869600
C	-1.54306000	-0.61178000	-1.49952400
C	-2.23792200	-0.27590600	-2.66006000
H	-5.37889500	-2.30855800	0.49206200
H	-4.14162800	-0.26885100	-3.67427300
H	-5.37973100	-1.33769300	-1.81062400
C	-4.31428800	-2.10483600	0.57499500
C	-1.54589300	-1.56701100	0.79729500
H	-1.70537700	0.19671600	-3.48196000
C	-2.23961900	-2.16258600	1.84873900
C	-3.61938100	-2.42708400	1.72622900
H	-1.70785300	-2.41519100	2.76306800
H	-4.14124000	-2.89081800	2.56027100
N	-0.19018900	-1.27826300	0.87681100
N	-0.18532500	-0.36515900	-1.35491400
H	0.24544500	-1.49649500	1.76614900
H	0.25094400	0.11419100	-2.13463500
B	0.55508500	-0.60287300	-0.14737400
B	2.17238100	-0.12456400	0.05886100
O	2.98570900	-0.64030900	1.09167700
O	2.96995800	0.20455100	-1.05530300
C	4.34692000	-0.73326600	0.60492400
C	4.35162400	0.25457200	-0.61797900
C	4.58006200	-2.19331300	0.19603200
H	3.90751500	-2.49178500	-0.61527600
H	5.61336600	-2.36521900	-0.12614900
H	4.37951700	-2.83859900	1.05898100
C	5.30749500	-0.35567400	1.72893400
H	6.34295000	-0.34685800	1.36780700
H	5.07536500	0.62853200	2.14473600
H	5.23749200	-1.09268900	2.53778300

C	4.68283800	1.70004200	-0.22800600
H	5.73937400	1.80506700	0.04327200
H	4.48417200	2.35272100	-1.08604000
H	4.07011500	2.04415600	0.60880000
C	5.24474900	-0.17457600	-1.77829100
H	6.29083900	-0.24276900	-1.45668500
H	4.94160500	-1.14259900	-2.18657900
H	5.18600600	0.56799200	-2.58297400

5

C	3.48448600	-2.36275800	-0.49171500
O	3.81271600	-2.87048100	-1.56077900
C	2.65919200	-1.20118300	-0.36128400
C	1.96540200	-0.18970300	-0.29820200
O	3.87095000	-2.84769400	0.70730900
C	4.72169100	-4.02739800	0.69316500
H	5.63027400	-3.79262500	0.13025700
H	4.18983400	-4.83267600	0.17745900
C	5.02627600	-4.38415600	2.13292600
H	5.66756300	-5.27323800	2.15542800
H	5.55285000	-3.56733100	2.63957200
H	4.10772700	-4.60869400	2.68704400
C	-5.05028600	-0.00336600	2.11507600
C	-5.52877500	-0.88799300	1.16553300
C	-4.69749000	-1.30008600	0.08558100
C	-3.36427900	-0.78061500	0.00395200
C	-2.89571500	0.13515300	1.00094000
C	-3.73989500	0.51324800	2.04359500
H	-6.15302000	-2.60362900	-0.85733200
H	-5.69258300	0.30518400	2.93694200
H	-6.54102300	-1.27918200	1.22978900
C	-5.14196800	-2.20846800	-0.91610000
C	-2.50926900	-1.18084200	-1.07367500
H	-3.37789400	1.20809300	2.79767500
C	-2.98219200	-2.07310900	-2.03453100
C	-4.29569000	-2.57852400	-1.94572700
H	-2.32932300	-2.37341200	-2.85071300
H	-4.64363200	-3.27321900	-2.70697200
N	-1.22294600	-0.65678300	-1.12032800
N	-1.59988600	0.61573500	0.88376500
H	-0.65722000	-0.98096500	-1.89734400
H	-1.29979500	1.26995900	1.59911100
B	-0.68371000	0.27524800	-0.16701100
B	0.88623400	1.00962200	-0.21687000
O	0.99887200	1.95492000	-1.36522500
O	1.15515800	1.85079500	0.99487700
C	1.73035700	3.09692900	-0.91169000
C	1.27588300	3.21495800	0.58658900
C	1.36201000	4.29700500	-1.78356000
H	0.27836300	4.43842500	-1.83272800
H	1.81718200	5.21849100	-1.39934000
H	1.72957000	4.14112500	-2.80564000
C	3.24047500	2.82783600	-1.04095300
H	3.83475200	3.71701900	-0.79818300
H	3.56230600	2.01077800	-0.38808700
H	3.46240000	2.54310000	-2.07649300
C	2.27939100	3.92534900	1.49607700
H	2.47075900	4.94929400	1.15070700
H	1.88048900	3.98379600	2.51671700
H	3.23152600	3.38823100	1.53694600
C	-0.09693400	3.89807500	0.72040000
H	-0.04360000	4.97385600	0.51335500
H	-0.82923200	3.44973200	0.04167600
H	-0.45865000	3.76845900	1.74765700

6-ts

C	-1.07232700	2.98877300	0.13975500
O	-1.62687500	3.22288500	1.22164600
C	0.33252300	2.87015900	-0.05677700
C	1.26040200	1.98633600	-0.07682600
O	-1.77465900	2.90443000	-1.03334300
C	-3.21405700	3.03610900	-0.95551800
H	-3.50628100	3.38174000	-1.95093400
H	-3.46443200	3.80821300	-0.22322200
C	-3.88218400	1.71273600	-0.61911200
H	-4.97229700	1.84078500	-0.62900000
H	-3.61939500	0.94463800	-1.35413200
H	-3.58486300	1.36020100	0.37286400

C	-3.16102700	-2.23467800	-2.21407400
C	-3.82622300	-2.43982200	-1.01921200
C	-3.27905700	-1.95078500	0.20051300
C	-2.03206700	-1.24702800	0.16516000
C	-1.37190300	-1.03993600	-1.08879000
C	-1.93531100	-1.53898700	-2.26071900
H	-4.87759700	-2.66298100	1.48246200
H	-3.58702100	-2.61171100	-3.14103800
H	-4.77231800	-2.97453900	-0.99406700
C	-3.93067700	-2.12985700	1.45320600
C	-1.46867200	-0.74075500	1.38072300
H	-1.42701300	-1.37617900	-3.20808900
C	-2.13634000	-0.93599200	2.58727800
C	-3.36473300	-1.62857100	2.61108600
H	-1.70441900	-0.54456800	3.50511200
H	-3.87128600	-1.76686200	3.56356400
N	-0.25920300	-0.06021700	1.31054300
N	-0.18051300	-0.32898500	-1.08945100
H	0.09340900	0.29667300	2.19156700
H	0.26440100	-0.21057300	-1.99294000
B	0.41826600	0.21762900	0.08455700
B	2.21530700	0.84208300	-0.03526300
O	2.97383800	0.46048200	1.13264500
O	2.86893600	0.32113700	-1.20877300
C	4.16271500	-0.21523200	0.67404600
C	3.74426900	-0.72963400	-0.75045200
C	4.52164400	-1.31817900	1.66614700
H	3.67763200	-1.99081800	1.84294700
H	5.36969500	-1.90984800	1.30065500
H	4.80881200	-0.87352700	2.62662300
C	5.29726300	0.81699800	0.61488600
H	6.25282700	0.35652400	0.33846200
H	5.07390700	1.61258200	-0.10420400
H	5.41286100	1.27387200	1.60456600
C	4.89826200	-0.87008800	-1.73865000
H	5.64488300	-1.58066300	-1.36442400
H	4.52221600	-1.24758500	-2.69725500
H	5.38992800	0.08950900	-1.92028800
C	2.95594000	-2.04626000	-0.69154100
H	3.60658200	-2.88984800	-0.43538200
H	2.14592000	-2.00071700	0.04269500
H	2.51345200	-2.24177200	-1.67483400

7

C	-2.36004200	2.39512800	-0.70182600
O	-2.92064300	2.60127900	-1.79199700
C	-0.99003900	2.01403800	-0.52346400
C	-0.53377500	0.73307900	-0.33658800
O	-2.99085500	2.69228100	0.48451900
C	-4.33631300	3.19815800	0.39919800
H	-4.95518100	2.48675400	-0.15847800
H	-4.33411500	4.14781700	-0.14881700
C	-4.84989700	3.38005400	1.81508400
H	-5.87754000	3.76294300	1.78876400
H	-4.85226500	2.42647200	2.35636100
H	-4.23142100	4.09438300	2.37157600
C	5.55908700	2.38967400	-0.12965700
C	6.10474200	1.13797000	0.09152300
C	5.26035000	-0.00364800	0.19363400
C	3.84423500	0.17082200	0.06314600
C	3.30599000	1.47972400	-0.16639000
C	4.16694400	2.57263900	-0.25986400
H	6.84572200	-1.44891300	0.51978000
H	6.21162000	3.25661000	-0.20576000
H	7.17964400	1.00865600	0.18982300
C	5.77165900	-1.31282200	0.42107700
C	2.97514400	-0.96380200	0.16380800
H	3.75368000	3.56325500	-0.43348800
C	3.51494100	-2.22928100	0.38764200
C	4.91028200	-2.39091100	0.51401800
H	2.85076700	-3.08685900	0.46315500
H	5.30983800	-3.38754200	0.68784000
N	1.60930500	-0.76057700	0.03362300
N	1.93321500	1.60794900	-0.28785300
H	1.01823700	-1.58228800	0.11803700
H	1.57136100	2.54350100	-0.44933600
B	1.00276600	0.52025600	-0.19609400

B	-1.49722100	-0.48200000	-0.23875500
O	-1.06619100	-1.76442100	0.08696900
O	-2.86121900	-0.44959500	-0.44642100
C	-2.18011200	-2.68424500	-0.12363400
C	-3.41732000	-1.72468900	-0.01159000
C	-2.11796500	-3.78154400	0.93071400
H	-2.08363200	-3.36975900	1.94282500
H	-2.99101500	-4.43938600	0.85020800
H	-1.21924200	-4.38957400	0.77694900
C	-2.00135600	-3.28292500	-1.52166900
H	-2.77387100	-4.02903400	-1.73615300
H	-2.03795100	-2.51059600	-2.29729900
H	-1.02445800	-3.77593300	-1.57528200
C	-4.58774000	-2.08559600	-0.91693600
H	-4.97410400	-3.07969700	-0.66398900
H	-5.39760800	-1.36038400	-0.77859400
H	-4.30054700	-2.08031000	-1.97159200
C	-3.90479500	-1.52857000	1.42687400
H	-4.39455800	-2.43112900	1.80798500
H	-3.08003400	-1.26858000	2.09885700
H	-4.63154700	-0.70915600	1.44532800

8-ts

C	2.44384100	2.23324500	-1.51343300
O	3.13986500	2.17350100	-2.52606900
C	1.16319800	1.61835100	-1.35159200
C	0.08762400	1.01617000	-1.18891900
O	2.83497300	2.89812100	-0.40005100
C	4.15652100	3.49674900	-0.42831100
H	4.19378200	4.22862400	-1.24139000
H	4.88944000	2.71062600	-0.63530900
C	4.39208500	4.14328900	0.92116000
H	5.38656500	4.60502800	0.93240600
H	3.64849600	4.92348900	1.12041900
H	4.34799800	3.40165100	1.72693800
C	-5.37931600	-0.85899500	-1.83641900
C	-5.85168700	0.05673800	-0.91296000
C	-4.98692100	0.56388800	0.09790400
C	-3.62518300	0.11710800	0.13391200
C	-3.16094400	-0.83485700	-0.83504700
C	-4.04471500	-1.31043800	-1.80674600
H	-6.46449400	1.83513500	1.05188600
H	-6.04627700	-1.24258100	-2.60561900
H	-6.88380700	0.39695800	-0.94325000
C	-5.43272800	1.49360000	1.07955300
C	-2.74189000	0.60341200	1.15580500
H	-3.68945900	-2.02968300	-2.54124600
C	-3.22216900	1.51132200	2.10242800
C	-4.56135900	1.94740400	2.05382900
H	-2.55004900	1.88004200	2.87395000
H	-4.90983800	2.65715900	2.80111900
N	-1.44096000	0.14201900	1.17382100
N	-1.85004000	-1.26092300	-0.76232100
H	-0.84616500	0.56682300	1.87604700
H	-1.56284000	-1.88638600	-1.50651100
B	-0.84889900	-0.62013200	0.08128300
B	0.70137000	-1.28532800	0.23652300
O	1.57115000	-0.99298300	1.26958000
O	1.23081100	-2.22527400	-0.62800700
C	2.86266900	-1.61148300	0.96828300
C	2.45936700	-2.75184400	-0.03358800
C	3.48850100	-2.09331000	2.27083900
H	2.81479000	-2.75027700	2.82723300
H	4.41948100	-2.63617600	2.07039400
H	3.72832000	-1.23191100	2.90467000
C	3.74285700	-0.53288700	0.33217100
H	4.75441900	-0.91082300	0.14929600
H	3.32823300	-0.18342100	-0.61650900
H	3.81159300	0.32140000	1.01328900
C	3.46920500	-3.01606200	-1.14323900
H	4.42725700	-3.33775400	-0.71870300
H	3.10063600	-3.81687300	-1.79447200
H	3.63972700	-2.12788700	-1.75704000
C	2.08460300	-4.06482400	0.65954500
H	2.96639700	-4.55375500	1.08743400
H	1.35350800	-3.90346100	1.45890100
H	1.64119500	-4.74270900	-0.07813300

9

C	-0.14179800	3.45289100	-1.04445500
O	-0.08975400	3.85540400	-2.20285600
C	-0.12789900	2.07252400	-0.66115700
C	-0.12831700	0.87729500	-0.38153300
O	-0.21912500	4.27380300	0.02294800
C	-0.24394500	5.70274700	-0.25057900
H	-1.11373400	5.91954200	-0.87821700
H	0.66183100	5.96489400	-0.80569300
C	-0.31678500	6.41831000	1.08178000
H	-0.33592300	7.50068700	0.90798500
H	-1.22468300	6.14187300	1.62991400
H	0.55520100	6.18505000	1.70336100
C	-4.17571700	-2.12299100	-2.20179200
C	-4.89800800	-1.86856500	-1.04794500
C	-4.22006300	-1.50429800	0.15069500
C	-2.79087800	-1.38083500	0.13757200
C	-2.05937600	-1.64797300	-1.07701700
C	-2.77243000	-2.01884300	-2.22730300
H	-6.01097300	-1.36373200	1.37128200
H	-4.69824800	-2.40506000	-3.11409400
H	-5.98210100	-1.95297700	-1.03718000
C	-4.92697700	-1.27773900	1.36633500
C	-2.08807200	-1.05254100	1.35424800
H	-2.22335400	-2.21921000	-3.14533400
C	-2.82935100	-0.85430300	2.52910100
C	-4.23219800	-0.96666600	2.52315700
H	-2.30246900	-0.60542100	3.44825200
H	-4.77647300	-0.79923200	3.45085300
N	-0.71857900	-0.97708700	1.32853100
N	-0.69087200	-1.56284800	-1.05601300
H	-0.30672200	-0.65160600	2.19657400
H	-0.25434400	-1.67976600	-1.96410300
B	0.01122400	-0.68746700	0.00137600
B	1.70640500	-0.97201900	0.08137900
O	2.47791400	-0.82763400	1.22184200
O	2.48025100	-1.34267500	-1.00505800
C	3.88742800	-0.88869800	0.83364200
C	3.82263600	-1.66988200	-0.52508700
C	4.67497700	-1.58521700	1.93581500
H	4.25501100	-2.56510100	2.17791200
H	5.72019000	-1.71851000	1.63324900
H	4.66034300	-0.97209900	2.84415500
C	4.36520700	0.55775800	0.67790500
H	5.43162900	0.59946600	0.43142100
H	3.80627700	1.08387900	-0.10328100
H	4.21045200	1.08787400	1.62414800
C	4.83996900	-1.22652800	-1.56889200
H	5.86033500	-1.38530200	-1.20094000
H	4.71294100	-1.81847700	-2.48252800
H	4.72280000	-0.17071500	-1.82688900
C	3.87281000	-3.19074600	-0.35491200
H	4.87082500	-3.52491200	-0.05145000
H	3.14774200	-3.53608000	0.38969800
H	3.62988600	-3.66347000	-1.31296400

10-ts

C	3.55198800	-1.44221800	-0.49003700
O	4.13107000	-1.27410600	-1.57313500
C	2.26961200	-2.01908900	-0.32500300
C	1.01548200	-1.81425100	-0.21349600
O	4.15100800	-1.14415400	0.70861000
C	5.53246800	-0.71667700	0.67107900
H	5.65663900	-0.12822000	1.58404500
H	5.68711500	-0.06719800	-0.19400600
C	6.48324700	-1.90292900	0.65148300
H	7.52012700	-1.54468000	0.68433000
H	6.31606200	-2.55057900	1.52021100
H	6.35344000	-2.49664200	-0.25957200
C	-4.40596400	-0.35084100	2.54619700
C	-5.12373000	-0.05826000	1.39985000
C	-4.54407500	-0.28292700	0.11861500
C	-3.21244800	-0.80728100	0.03715200
C	-2.48814300	-1.10619700	1.24419800
C	-3.09824300	-0.87041600	2.48069500
H	-6.26423900	0.39321000	-1.02035700
H	-4.85292800	-0.17615200	3.52279600

H	-6.13318200	0.34108700	1.45944400
C	-5.25488200	-0.00583800	-1.08386900
C	-2.61883300	-1.05212900	-1.25100900
H	-2.54756700	-1.09087200	3.39268600
C	-3.35544500	-0.76603200	-2.40531800
C	-4.66193900	-0.24804100	-2.31056800
H	-2.90410600	-0.94505300	-3.37885700
H	-5.20868100	-0.03301100	-3.22649200
N	-1.34724900	-1.57864000	-1.30928200
N	-1.21974700	-1.63511600	1.14677300
H	-0.94734800	-1.61108300	-2.24036300
H	-0.72563700	-1.70310000	2.02930700
B	-0.43989400	-1.53223000	-0.12279300
B	0.54868100	0.11105700	-0.11365700
O	0.69068700	0.90893900	-1.22377300
O	0.79637700	0.77945200	1.06355000
C	1.26483100	2.18654900	-0.77867500
C	0.87993800	2.20875700	0.74391900
C	0.65496400	3.30749000	-1.60850300
H	-0.43749400	3.28499100	-1.58014800
H	0.99440800	4.28208000	-1.23908400
H	0.97416800	3.20966800	-2.65232500
C	2.77302400	2.10602800	-1.01896200
H	3.26065500	3.05402000	-0.76716400
H	3.23336100	1.31013500	-0.42764400
H	2.95558800	1.89514700	-2.07768600
C	1.91385500	2.85651600	1.65450800
H	2.05437000	3.90890900	1.38164600
H	1.56649200	2.81820900	2.69311100
H	2.88018500	2.34911200	1.59699100
C	-0.50772700	2.79714700	1.01158400
H	-0.51695000	3.88039900	0.84987600
H	-1.26888600	2.34145900	0.36979900
H	-0.78156700	2.60310900	2.05421800

11-ts

C	2.34535000	1.88661600	0.07027000
O	2.82750800	2.34119200	1.11193700
C	0.98713800	1.38446600	-0.06537300
C	0.62886600	0.06398600	-0.00443800
O	2.99334700	1.98328600	-1.12441200
C	-5.64281100	0.81367200	-0.56342700
C	-6.00696800	-0.52055900	-0.51606800
C	-5.01109400	-1.52856700	-0.38210800
C	-3.63639600	-1.13326600	-0.29682500
C	-3.28473200	0.25543100	-0.35017800
C	-4.29343100	1.21250700	-0.48213800
H	-6.37089900	-3.21998200	-0.39504900
H	-6.41000500	1.57817500	-0.66597300
H	-7.05127500	-0.81576300	-0.58049300
C	-5.32919400	-2.91562000	-0.33000100
C	-2.61744000	-2.12913100	-0.15879700
H	-4.02772000	2.26520100	-0.52204300
C	-2.96872500	-3.47783600	-0.11246200
C	-4.32423500	-3.85714600	-0.19878900
H	-2.18959100	-4.22918000	-0.00838000
H	-4.57721600	-4.91440400	-0.16052000
N	-1.29905900	-1.70985500	-0.07337300
N	-1.94965400	0.60969000	-0.26723900
H	-0.59956200	-2.44089500	0.01823500
H	-1.74585900	1.62038100	-0.30241600
B	-0.88239400	-0.33470200	-0.11860500
B	1.70980500	-1.04335100	0.16714100
O	1.43777200	-2.40159600	0.06706200
O	3.04373900	-0.81506800	0.42788900
C	2.62827400	-3.13385800	0.49560400
C	3.75990700	-2.07396800	0.25593600
C	2.75048300	-4.40358500	-0.33534700
H	2.73400500	-4.18880200	-1.40695900
H	3.68419000	-4.92608200	-0.09757300
H	1.91689100	-5.07702400	-0.10631400
C	2.42428200	-3.48175300	1.97217900
H	3.25781800	-4.07857500	2.35720600
H	2.32649800	-2.58122000	2.58770600
H	1.50541700	-4.06914900	2.07596800
C	4.90781100	-2.12724200	1.25554200
H	5.40924200	-3.10074100	1.20856900

H	5.64417000	-1.35299800	1.01256600
H	4.56187300	-1.96117100	2.27909700
C	4.30466100	-2.08168700	-1.17529200
H	4.91018600	-2.97425500	-1.36530600
H	3.49661500	-2.04413000	-1.91360100
H	4.93810200	-1.19970100	-1.31772900
C	-1.05652100	4.09887000	0.74038300
O	-0.88634800	3.22165000	-0.36334200
H	0.01126400	2.45884700	-0.20200400
C	0.21359000	4.94762200	0.93761800
H	0.43111700	5.51780000	0.02511200
H	0.09957000	5.65859700	1.76722700
H	1.07593500	4.30596400	1.15280100
C	-2.24847400	5.01533100	0.42968400
H	-3.15831500	4.41990000	0.28261900
H	-2.43251600	5.72755100	1.24542400
H	-2.06156800	5.58540900	-0.48926400
C	-1.34794800	3.29864600	2.02673000
H	-0.52055500	2.61561100	2.25266000
H	-1.48618600	3.96503400	2.88869300
H	-2.25994300	2.70066500	1.90871800
C	4.31539200	2.56634700	-1.10608300
C	4.83886200	2.56348000	-2.52872400
H	4.95355500	1.97242800	-0.44312400
H	4.25341800	3.58279100	-0.70303800
H	5.84628700	2.99665500	-2.54915200
H	4.89558500	1.54312600	-2.92561300
H	4.19538800	3.15911500	-3.18690700

12

C	2.78546000	1.30057300	-0.94511200
O	3.60962100	0.39210000	-0.92102200
C	1.32290400	1.12477100	-0.81155900
C	0.78989100	-0.06196500	-0.44877600
O	3.11915900	2.59343800	-1.11223900
C	-5.34584700	1.47195000	-0.17317500
C	-5.86667900	0.19091600	-0.23714500
C	-4.99469300	-0.92924500	-0.32888300
C	-3.57936100	-0.70214500	-0.34613800
C	-3.06194300	0.63528400	-0.27038700
C	-3.95681100	1.70580900	-0.19048400
H	-6.54974700	-2.44106200	-0.39467000
H	-6.01998900	2.32360400	-0.10851900
H	-6.94087500	0.02365100	-0.22308100
C	-5.47650600	-2.26772100	-0.40865400
C	-2.69005600	-1.81836300	-0.44862900
H	-3.55910100	2.71421800	-0.14276000
C	-3.19648000	-3.11319500	-0.52815400
C	-4.59118600	-3.32506700	-0.50624500
H	-2.51008400	-3.95261300	-0.60729900
H	-4.96939900	-4.34293500	-0.56852700
N	-1.32555500	-1.55936200	-0.46786300
N	-1.69032100	0.84018100	-0.28052600
H	-0.73170900	-2.37232200	-0.59095200
H	-1.37235500	1.86922200	-0.19035800
B	-0.77349900	-0.23724700	-0.39097700
B	1.66534800	-1.30319400	-0.06211100
O	1.74608500	-2.43166600	-0.84647300
O	2.21757300	-1.47174400	1.18381300
C	2.26810100	-3.51744100	-0.00863200
C	2.95832900	-2.73548900	1.16992400
C	3.20970700	-4.36926500	-0.84841800
H	3.99981800	-3.76791800	-1.30502600
H	3.67353900	-5.14628700	-0.23020000
H	2.64554200	-4.86252700	-1.64785800
C	1.05951800	-4.34500700	0.43513700
H	1.37159500	-5.21431800	1.02299500
H	0.36201200	-3.75306300	1.03657700
H	0.52889000	-4.70553900	-0.45289400
C	2.81806900	-3.39374100	2.53631700
H	3.29495500	-4.38046400	2.53323000
H	3.31641600	-2.77803500	3.29339300
H	1.77089600	-3.51268200	2.82611200
C	4.42460000	-2.39253500	0.89874100
H	5.04816000	-3.29245300	0.92435400
H	4.54999300	-1.89814100	-0.06784900
H	4.77815800	-1.70847500	1.67803900

C	-0.62868400	3.98063700	1.03571400
O	-0.91578600	3.38130700	-0.18150300
H	0.70767200	2.01066700	-0.97343500
C	-0.00165900	5.37813900	0.81118700
H	-0.69605300	6.01374000	0.24473900
H	0.24263900	5.89240000	1.75302600
H	0.92188300	5.28237000	0.22434900
C	-1.91412700	4.15674400	1.88637400
H	-2.36795400	3.17858000	2.09202300
H	-1.72028400	4.65114000	2.85019500
H	-2.64915300	4.75874000	1.33522500
C	0.37427000	3.13282500	1.86434700
H	1.32168300	3.02334200	1.32260200
H	0.59141800	3.58361000	2.84418800
H	-0.02996500	2.12657500	2.03550300
C	4.53112300	2.90154600	-1.24979700
C	4.65616300	4.40175200	-1.41273100
H	4.92048100	2.36185700	-2.11883100
H	5.05215800	2.54352800	-0.35641800
H	5.71496800	4.66759900	-1.51471900
H	4.12666900	4.74700600	-2.30804700
H	4.25118400	4.92736400	-0.54048900

13-ts

C	0.78528100	-2.41531800	0.03746400
O	0.59211600	-3.08886900	-0.98280700
C	1.26435300	-1.03812500	0.04969200
C	0.43032600	0.03927100	-0.03468400
O	0.67242100	-2.94524300	1.28269300
C	-5.28569000	-2.46474600	-1.19640000
C	-6.05472300	-1.42615000	-0.70426600
C	-5.43132100	-0.26597400	-0.16470900
C	-4.00043900	-0.19952600	-0.14101100
C	-3.22938900	-1.29077900	-0.65498200
C	-3.87641900	-2.40832400	-1.17707900
H	-7.26261600	0.78371300	0.33644900
H	-5.76983400	-3.34737600	-1.60795100
H	-7.14026200	-1.48018600	-0.72304400
C	-6.17672200	0.83170700	0.35133500
C	-3.35211100	0.95996000	0.39286000
H	-3.28553600	-3.23352100	-1.56710600
C	-4.11586800	2.01420400	0.88783700
C	-5.52429500	1.93860600	0.86240800
H	-3.61690000	2.89115300	1.29288100
H	-6.10193300	2.77253600	1.25454500
N	-1.96483800	0.99009000	0.39817800
N	-1.84307600	-1.19632500	-0.62002900
H	-1.53417100	1.82434100	0.78647900
H	-1.34593500	-1.97922200	-1.03170600
B	-1.13137700	-0.07133400	-0.08535400
B	1.01297100	1.48344000	-0.01160300
O	0.51196200	2.46050000	0.83756700
O	1.97610300	1.98447900	-0.85554900
C	1.01221200	3.75391100	0.37243800
C	2.30825900	3.33680000	-0.40808600
C	1.24836700	4.65382000	1.57718500
H	1.89945100	4.17986900	2.31608400
H	1.70566300	5.59899300	1.26289100
H	0.29157700	4.88331800	2.05978900
C	-0.07579600	4.34648100	-0.52735600
H	0.20277900	5.34496800	-0.88034700
H	-0.26906300	3.71022700	-1.39759900
H	-1.00524800	4.43343100	0.04591400
C	2.62204700	4.19221100	-1.62813700
H	2.79088100	5.23350300	-1.33052500
H	3.53436400	3.82415800	-2.11061800
H	1.81172800	4.16582700	-2.36139400
C	3.54275100	3.22020100	0.48878600
H	3.88222200	4.20633600	0.82297000
H	3.34433100	2.60119000	1.36986000
H	4.35299800	2.75166400	-0.07936000
C	4.69779000	-1.53644800	-0.24586600
O	3.82636700	-0.90102500	0.65687600
H	2.63067600	-0.96654800	0.29255800
C	0.31570300	-4.34723400	1.39576400
C	1.54067700	-5.24297400	1.32612800
H	-0.40851400	-4.59472200	0.61572900

H	-0.17157500	-4.42038500	2.37127500
H	1.23860000	-6.28518500	1.49072100
H	2.02698700	-5.17458900	0.34838300
H	2.26725800	-4.97011200	2.09998800
C	4.60522200	-0.88972400	-1.64647500
H	4.85204500	0.17785100	-1.58696100
H	5.29149900	-1.36264400	-2.36307300
H	3.58586900	-0.97758300	-2.04258300
C	4.36327600	-3.04098800	-0.36622900
H	5.06745000	-3.56596200	-1.02728300
H	4.40302100	-3.51466600	0.62290000
H	3.35414800	-3.18250800	-0.76906000
C	6.13662500	-1.38621000	0.27978800
H	6.86954400	-1.86136500	-0.38764000
H	6.39564100	-0.32355000	0.37354100
H	6.22782200	-1.84648800	1.27236900

14

C	1.04118800	-2.21242800	-0.16795900
O	0.61202900	-2.85901800	-1.11849300
C	1.28153800	-0.73925100	-0.18614100
C	0.28630900	0.18044100	-0.21437100
O	1.37712300	-2.75850500	1.00718500
C	-5.15656700	-2.98038400	-1.09363400
C	-6.00358000	-2.06203100	-0.50131400
C	-5.48393500	-0.85452900	0.04414300
C	-4.07372500	-0.61510400	-0.03127100
C	-3.22082900	-1.58464400	-0.64799200
C	-3.76642500	-2.75255700	-1.17275600
H	-7.38319000	-0.05337100	0.71966400
H	-5.56226500	-3.89973600	-1.50931800
H	-7.07326100	-2.24716000	-0.44583600
C	-6.31269700	0.12563600	0.65998600
C	-3.53010700	0.59459200	0.50619000
H	-3.11273900	-3.48414900	-1.64128000
C	-4.37215200	1.53048600	1.09971200
C	-5.75983300	1.28516200	1.17112300
H	-3.95117700	2.44680600	1.50586400
H	-6.40091800	2.02858800	1.63907700
N	-2.15858800	0.79427900	0.41227300
N	-1.85477400	-1.32442700	-0.70480000
H	-1.80444400	1.66719400	0.79295600
H	-1.29305600	-2.03081600	-1.16863800
B	-1.25158600	-0.14364400	-0.17259900
B	0.69021700	1.69005800	-0.18470600
O	0.07957500	2.59636800	0.66085800
O	1.62028200	2.27409900	-1.00286700
C	0.46884900	3.93941700	0.21785600
C	1.81406900	3.65197800	-0.53923400
C	0.59886100	4.84069500	1.43713200
H	1.27138500	4.41496400	2.18608000
H	0.98169100	5.82407400	1.14159600
H	-0.38504600	4.98304400	1.89801600
C	-0.65152700	4.43622200	-0.69837300
H	-0.46080100	5.46094500	-1.03383000
H	-0.76379500	3.79606800	-1.57982900
H	-1.59674500	4.42690400	-0.14483900
C	2.06708700	4.54134100	-1.74858700
H	2.12605900	5.59168600	-1.44135900
H	3.02092300	4.26826300	-2.21321400
H	1.27831300	4.44108500	-2.49870000
C	3.03638200	3.64183900	0.38029400
H	3.27171400	4.65215800	0.73071600
H	2.88036700	2.99630700	1.25086000
H	3.89849800	3.26071600	-0.17624300
C	5.17457200	-1.27713300	0.07135000
O	4.31941700	-0.20338900	-0.00221300
H	2.35990900	-0.47653800	-0.13712800
C	1.20236000	-4.19901700	1.16950400
C	2.39534900	-4.97327900	0.63999300
H	0.27345100	-4.49268400	0.67540700
H	1.09237800	-4.32484000	2.24839200
H	2.24359800	-6.04005600	0.84685000
H	2.50941600	-4.84567500	-0.44062100
H	3.31982000	-4.65527100	1.13250100
C	6.65054400	-0.83771800	-0.14014900
H	6.93126600	-0.09918200	0.62389100

H	7.36612500	-1.67352400	-0.08714100
H	6.75784100	-0.35902900	-1.12355700
C	4.83997600	-2.33784500	-1.01713600
H	5.49700500	-3.22093000	-0.98071300
H	3.80582400	-2.68262100	-0.90021600
H	4.93070400	-1.88595800	-2.01483900
C	5.08330800	-1.97416400	1.45889500
H	5.74462400	-2.85020500	1.55134200
H	5.35306100	-1.25908200	2.24890200
H	4.05235500	-2.30181100	1.64006800

15-ts

C	-1.86583300	2.50564000	-0.70967900
O	-2.33730700	2.80356100	-1.81133400
C	-0.64738400	1.75007100	-0.50118500
C	-0.52413100	0.40597400	-0.30378300
O	-2.39796900	2.97801100	0.45113600
C	-3.56631200	3.82501700	0.34156200
H	-4.35478400	3.27548600	-0.18288800
H	-3.31046300	4.70897200	-0.25246100
C	-3.99165000	4.20221600	1.74663800
H	-4.87923600	4.84474200	1.69992000
H	-4.24276300	3.31134100	2.33411900
H	-3.19718900	4.75108500	2.26576600
C	5.35183300	2.31384400	-0.22774200
C	6.00015600	1.11446300	0.02157800
C	5.24810900	-0.08654100	0.16422600
C	3.81970400	-0.02913900	0.04586300
C	3.15873900	1.23519300	-0.21481400
C	3.95156100	2.38725300	-0.34655600
H	6.95237700	-1.38535900	0.51028600
H	5.93634800	3.22588600	-0.33533100
H	7.08309400	1.07314100	0.11022600
C	5.86934800	-1.34286300	0.42122000
C	3.05399600	-1.23461700	0.18964700
H	3.46327100	3.33883200	-0.54270600
C	3.70020600	-2.44602500	0.44101100
C	5.10432800	-2.48763700	0.55441400
H	3.10932200	-3.35273600	0.54868500
H	5.58895900	-3.44173800	0.75062900
N	1.66840300	-1.15992200	0.07518600
N	1.79941500	1.25113600	-0.32213200
H	1.15965200	-2.03014000	0.19078400
H	0.78732400	2.05477100	-0.48529000
B	1.01297200	0.08568200	-0.17533600
B	-1.66396500	-0.62898500	-0.20233200
O	-1.40893800	-1.97026700	0.03388900
O	-3.01346300	-0.37064900	-0.32254200
C	-2.66253500	-2.69870100	-0.14677900
C	-3.73335800	-1.57383800	0.08733400
C	-2.71306400	-3.84940200	0.84983200
H	-2.55859800	-3.50488000	1.87571700
H	-3.68161300	-4.35938600	0.79383400
H	-1.93068900	-4.57876500	0.61122400
C	-2.65616800	-3.23995800	-1.57915300
H	-3.54292900	-3.85169500	-1.77610300
H	-2.62091200	-2.42959700	-2.31501800
H	-1.76895700	-3.86730600	-1.71808800
C	-4.99358400	-1.70445300	-0.75763500
H	-5.51089600	-2.64372900	-0.53041000
H	-5.67585700	-0.87732800	-0.53138900
H	-4.76847600	-1.68001000	-1.82700600
C	-4.10223900	-1.38667500	1.56159500
H	-4.69609700	-2.22869800	1.93268500
H	-3.21079000	-1.28527300	2.18987500
H	-4.69876300	-0.47378800	1.66516700

16

C	2.45199300	2.80180500	-0.19798400
O	2.79403300	3.96687800	-0.37931800
C	1.04058700	2.37817000	-0.11784800
C	0.58853700	1.11442900	0.03705400
O	3.32881700	1.78675600	-0.05770400
C	4.74480800	2.07990800	-0.18466800
H	5.23227500	1.38184600	0.49813900
H	4.92549100	3.10121900	0.15625800
C	5.20368700	1.87191500	-1.61666400
H	6.28508400	2.04457200	-1.68194100

H	4.99728500	0.84762200	-1.94719100
H	4.70225700	2.56846100	-2.29776700
C	-5.59770500	2.01363300	0.89952900
C	-6.04587100	0.80652500	0.37863000
C	-5.10889000	-0.13811300	-0.12394400
C	-3.70977800	0.18322900	-0.07836100
C	-3.24373600	1.44618200	0.46766200
C	-4.23221500	2.33549100	0.94848600
H	-6.56974200	-1.63531400	-0.70919200
H	-6.32217800	2.73150800	1.28161200
H	-7.10708600	0.57055200	0.34692600
C	-5.51029500	-1.39127600	-0.67436100
C	-2.76111300	-0.75990000	-0.58503800
H	-3.90604200	3.28764400	1.36191200
C	-3.18987400	-1.97920200	-1.12063600
C	-4.56524900	-2.28011000	-1.15797100
H	-2.45553000	-2.68419900	-1.50325500
H	-4.88530700	-3.23233600	-1.57649200
N	-1.42324200	-0.41581400	-0.51915500
N	-1.91607700	1.77645000	0.52190500
H	-0.77919700	-1.09515700	-0.91016100
B	-0.98174600	0.85326400	0.02990700
B	1.49251600	-0.15240200	0.19466300
O	1.70269600	-1.03527600	-0.84196600
O	1.94791700	-0.62173000	1.40134700
C	2.17292500	-2.29701000	-0.25876900
C	2.72409000	-1.83698500	1.14090900
C	3.21395700	-2.90483700	-1.18888100
H	4.02413900	-2.20382700	-1.40428700
H	3.64264800	-3.80875300	-0.74139000
H	2.74096000	-3.18608600	-2.13644900
C	0.95455800	-3.21773000	-0.15761300
H	1.24049400	-4.20515100	0.21947200
H	0.18619800	-2.80469100	0.50355400
H	0.51926900	-3.34512400	-1.15479000
C	2.47707800	-2.82029500	2.27747000
H	2.97314500	-3.77481700	2.06819700
H	2.89254300	-2.41794800	3.20818700
H	1.41094100	-3.00676000	2.43029400
C	4.19845700	-1.43202700	1.10660000
H	4.84203600	-2.31144700	0.99801300
H	4.40832600	-0.74439600	0.28423800
H	4.45388400	-0.93225100	2.04704700
H	0.34606600	3.21023300	-0.22352500

17-ts

C	2.45447500	1.90119800	-1.71663400
O	2.71459500	2.85824300	-2.43864600
C	1.08261500	1.39400000	-1.51027500
C	0.74020800	0.32153900	-0.76803400
O	3.38829000	1.21609300	-1.02747100
C	4.77957100	1.59778200	-1.19231000
H	5.24115900	1.36742800	-0.23055900
H	4.82989600	2.67457300	-1.36623500
C	5.42007600	0.81118900	-2.32156600
H	6.48156600	1.07840700	-2.39424800
H	5.34807500	-0.26650800	-2.13648500
H	4.94231400	1.03595500	-3.28145800
C	-5.53502200	0.81610700	-0.22865900
C	-5.84142800	-0.53366900	-0.29447600
C	-4.80093200	-1.49174300	-0.44247600
C	-3.44409200	-1.03211100	-0.51814000
C	-3.14250700	0.37513400	-0.44442600
C	-4.20759700	1.27674200	-0.30208400
H	-6.08725100	-3.24022400	-0.46234900
H	-6.33751700	1.54306300	-0.11754200
H	-6.87170900	-0.87655500	-0.23664800
C	-5.05881500	-2.89161100	-0.51921100
C	-2.38962800	-1.98749400	-0.66958000
H	-3.98073900	2.33647600	-0.24526700
C	-2.67822800	-3.34993400	-0.74185000
C	-4.01641800	-3.78856300	-0.66527900
H	-1.86616800	-4.06377400	-0.85762300
H	-4.22499600	-4.85468000	-0.72315500
N	-1.09126900	-1.50490000	-0.73954600
N	-1.83483100	0.82130000	-0.51005800
H	-0.37092800	-2.20753500	-0.86692900

B	-0.77916500	-0.10107500	-0.67120000
B	1.73225700	-0.61117600	0.00113600
O	2.14807300	-1.81739000	-0.51638400
O	2.06760400	-0.44369900	1.32021500
C	2.63477900	-2.62900200	0.60541000
C	2.97107500	-1.53820700	1.68705700
C	3.82772900	-3.44904200	0.13412200
H	4.60346900	-2.82051100	-0.31060600
H	4.26359600	-4.00025200	0.97498700
H	3.50155000	-4.17647200	-0.61764600
C	1.48352500	-3.55256900	1.00991700
H	1.78966100	-4.23544200	1.80923100
H	0.61030000	-2.98770300	1.35179600
H	1.18628000	-4.15281300	0.14310800
C	2.67169300	-1.94975500	3.12221700
H	3.26893400	-2.82589900	3.39936000
H	2.93301600	-1.13078300	3.80176100
H	1.61451800	-2.18879900	3.26378600
C	4.39740500	-0.99833800	1.57712400
H	5.12459900	-1.74103600	1.92188100
H	4.64347000	-0.72181000	0.54922300
H	4.49098700	-0.10648800	2.20583000
H	0.33111200	1.99067100	-2.02192900
H	-1.66862300	2.01333700	-0.27413300
O	-1.56997800	3.28625700	-0.05871100
C	-1.21424200	3.63831200	1.24999000
C	-2.19251500	3.01886500	2.27693200
C	0.21739300	3.15844100	1.58639900
C	-1.26538500	5.17285500	1.38307200
H	-3.21645800	3.36132800	2.08053100
H	-2.18256200	1.92419300	2.20453200
H	-1.93213300	3.29282000	3.30927200
H	0.93265600	3.56509700	0.85967600
H	0.53131900	3.47308900	2.59218600
H	0.28133500	2.06523000	1.53835500
H	-1.00452700	5.51197200	2.39570400
H	-0.56553900	5.63642200	0.67502600
H	-2.27451400	5.53688000	1.14864200

18-ts

C	2.09864100	2.55517900	0.22765300
O	2.42064600	2.92208800	1.36169100
C	0.90466400	1.79932200	-0.09295100
C	0.81919400	0.45892100	-0.33204000
O	2.79713700	2.93557700	-0.87739400
C	3.96359200	3.76493200	-0.66526100
H	4.65067200	3.24420000	0.00957400
H	3.65364100	4.69966200	-0.18549200
C	4.59806800	4.01669800	-2.01859900
H	5.48896200	4.64398700	-1.89366700
H	4.90362800	3.07550700	-2.49047300
H	3.90397600	4.53546600	-2.69018800
C	-5.05124000	2.30847100	-0.67197200
C	-5.64293500	1.16323900	-1.17839100
C	-4.86003500	-0.00325900	-1.41042100
C	-3.45746000	0.03328700	-1.11485800
C	-2.86109700	1.23547000	-0.57576300
C	-3.67685900	2.35491500	-0.36775200
H	-6.49004300	-1.23035300	-2.15110400
H	-5.65863600	3.19471700	-0.49846700
H	-6.70628500	1.13779500	-1.40396600
C	-5.42616700	-1.20419800	-1.92758300
C	-2.65867800	-1.13511600	-1.35435500
H	-3.23190100	3.25768900	0.04341800
C	-3.25050400	-2.29221000	-1.85876800
C	-4.63198700	-2.31574700	-2.14089600
H	-2.63552100	-3.17145200	-2.03634200
H	-5.07338000	-3.22844300	-2.53527000
N	-1.29418600	-1.07418500	-1.08126800
N	-1.52809000	1.22011600	-0.25132100
H	-0.75269900	-1.90130900	-1.30884100
H	-0.55349600	2.05173800	-0.09718100
B	-0.69488200	0.11524300	-0.56946500
B	1.96911700	-0.56842800	-0.30965900
O	1.77772800	-1.88277600	-0.70261800
O	3.25860300	-0.33597800	0.12021800
C	2.95720600	-2.64033000	-0.29174000

C	4.05305400	-1.51902000	-0.20110500
C	3.23432500	-3.72471000	-1.32396200
H	3.31569800	-3.31205000	-2.33299700
H	4.16594700	-4.25030800	-1.08473700
H	2.41930100	-4.45729700	-1.31679600
C	2.62156700	-3.27052500	1.06318100
H	3.43767800	-3.90861800	1.41838300
H	2.41811200	-2.50799900	1.82238300
H	1.72448500	-3.88953200	0.95329800
C	5.08982700	-1.72859200	0.89449400
H	5.64037600	-2.66112800	0.72485000
H	5.80914900	-0.90196100	0.88403000
H	4.62967800	-1.76978200	1.88518000
C	4.74558900	-1.23732100	-1.53724900
H	5.41358400	-2.05809100	-1.81940800
H	4.01951500	-1.08535100	-2.34297000
H	5.34439400	-0.32515100	-1.44055400
C	-2.80416700	-0.97623500	2.57391900
C	-1.66340600	-0.18683600	3.22985300
H	-3.76333100	-0.46930000	2.73331200
H	-2.64030000	-1.07103800	1.49519300
H	-2.87200600	-1.98674600	2.99571500
C	-0.31742400	-0.87819000	2.96823200
C	-1.90446500	-0.03669800	4.73162200
H	0.50513200	-0.28899800	3.39043100
H	-0.29846900	-1.87667900	3.42241900
H	-0.13950500	-0.99190800	1.89411100
H	-1.93163000	-1.01813400	5.21909300
H	-1.10367500	0.55482100	5.19196300
H	-2.85925100	0.46909100	4.91997400
O	-1.63749300	1.15784300	2.70686000
H	-1.55783200	1.11864800	1.72709000

19-ts

C	-1.30187900	2.47531400	-1.01450800
O	-1.98851200	2.16976800	-1.99688200
C	0.12256300	2.56760200	-0.98901700
C	1.12624100	1.82221800	-0.69985400
O	-1.86041300	2.83059100	0.18304100
C	-3.30178200	2.77037300	0.28966900
H	-3.48442300	2.59483400	1.35297700
H	-3.66800800	1.91117400	-0.27778900
C	-3.95533600	4.06307500	-0.17062400
H	-5.03966300	4.00354900	-0.01129300
H	-3.57217500	4.91764900	0.39975700
H	-3.77310700	4.24127200	-1.23556600
C	-2.85320600	-3.14056500	-1.87328300
C	-3.60277100	-3.04984900	-0.71478600
C	-3.17914100	-2.20485200	0.34959400
C	-1.96704300	-1.45609900	0.19625400
C	-1.21676800	-1.56200300	-1.01991500
C	-1.66178700	-2.40356600	-2.03669400
H	-4.83810600	-2.64210000	1.67694300
H	-3.18441700	-3.78896900	-2.68125100
H	-4.52160800	-3.61937200	-0.60039600
C	-3.91714800	-2.07656700	1.56017700
C	-1.51845400	-0.60841000	1.26057200
H	-1.08771000	-2.47872200	-2.95696100
C	-2.26886000	-0.50913900	2.43004100
C	-3.46441300	-1.24434300	2.56755000
H	-1.92471300	0.14129700	3.23061100
H	-4.03612300	-1.14986900	3.48797300
N	-0.33466200	0.09461800	1.08343000
N	-0.05973300	-0.80376000	-1.14295800
H	-0.07684700	0.71194400	1.84489600
H	0.44035700	-0.90085100	-2.01974700
B	0.41518400	0.08424400	-0.13161000
B	2.16102800	0.83284800	-0.28024800
O	2.81386100	0.84400400	1.00829300
O	2.98135100	0.10588600	-1.21451000
C	4.09982000	0.21132900	0.84568200
C	3.88588800	-0.69130300	-0.42290700
C	4.44057400	-0.55788100	2.11901300
H	3.64269000	-1.25393100	2.39276700
H	5.37098200	-1.12477900	1.99320900
H	4.58138600	0.14318100	2.95049400
C	5.14105700	1.31380400	0.61029200

H	6.15758000	0.90794300	0.55172000
H	4.93317000	1.86546200	-0.31285200
H	5.10451200	2.02103400	1.44695800
C	5.14927700	-0.95072900	-1.23776700
H	5.90847200	-1.45397600	-0.62716400
H	4.91500800	-1.60040100	-2.08977200
H	5.57410400	-0.02083100	-1.62601300
C	3.20712700	-2.02687500	-0.08534000
H	3.89384400	-2.70500300	0.43351200
H	2.32349700	-1.88396400	0.54436200
H	2.88694000	-2.50924000	-1.01581900

20-ts

C	-3.52430300	-1.66608100	-0.74755400
O	-4.11065500	-1.26932700	-1.76870100
C	-2.17867600	-2.08042900	-0.68303800
C	-0.93593700	-1.83455300	-0.55047800
O	-4.18701400	-1.84853800	0.44369400
C	-5.62156000	-1.66330800	0.44187100
H	-5.97759700	-2.31810600	1.24253000
H	-6.02558000	-2.01632000	-0.51079400
C	-6.02258100	-0.22141000	0.70804700
H	-7.11688900	-0.15579700	0.76589200
H	-5.60849400	0.13201300	1.65898400
H	-5.67899600	0.44116100	-0.09192900
C	4.69983900	0.34431000	-2.15753200
C	5.25664100	0.36961200	-0.89097300
C	4.53863000	-0.16840400	0.21495700
C	3.23531800	-0.72388400	-0.00303300
C	2.68011600	-0.74148500	-1.33051700
C	3.42281100	-0.20427100	-2.38708800
H	6.07193500	0.24788000	1.69434600
H	5.25252100	0.75714700	-2.99901500
H	6.24390100	0.79304400	-0.72339800
C	5.08386100	-0.17511700	1.53046000
C	2.50282900	-1.28146200	1.10343700
H	2.99956700	-0.21290700	-3.38925500
C	3.07993300	-1.26986300	2.37750500
C	4.36123300	-0.71965200	2.57753200
H	2.52394500	-1.68774700	3.21399700
H	4.78267200	-0.72236700	3.58071800
N	1.25982700	-1.82796700	0.87140000
N	1.43857400	-1.30652700	-1.52551300
H	0.75210200	-2.09195200	1.70823200
H	1.05883300	-1.17532900	-2.45626400
B	0.50614000	-1.52465600	-0.38022100
B	-0.56116000	0.05764600	-0.13851300
O	-0.82600100	0.57203000	1.10849500
O	-0.73871600	0.97633000	-1.14797300
C	-1.42732000	1.89991400	0.91586800
C	-0.91313200	2.28878600	-0.51559700
C	-0.95590400	2.81563100	2.03620200
H	0.13436900	2.83558700	2.11248500
H	-1.31342200	3.83799600	1.86696100
H	-1.36129000	2.46763500	2.99307100
C	-2.94147000	1.70539300	0.98071500
H	-3.46899400	2.66222300	0.90401300
H	-3.29297100	1.04906200	0.18072700
H	-3.20078500	1.24510000	1.93949600
C	-1.89272600	3.10668500	-1.34550800
H	-2.10216800	4.06319400	-0.85270700
H	-1.45632600	3.31919400	-2.32799200
H	-2.83695100	2.57734500	-1.49662200
C	0.46451500	2.95561700	-0.50518800
H	0.41009400	3.97080600	-0.09780000
H	1.18490500	2.37992900	0.08509100
H	0.83830900	3.01758300	-1.53293800

21

C	-2.37531400	-2.38563800	-0.63977800
O	-2.94467700	-2.56112000	-1.73144800
C	-1.00819800	-1.99721600	-0.46497800
C	-0.54562700	-0.71644900	-0.29617600
O	-2.99099700	-2.73423800	0.54073100
C	-4.32440200	-3.26968500	0.44574200
H	-4.30793000	-4.18032000	-0.16436800
H	-4.97267300	-2.54026000	-0.05250000
C	-4.80644600	-3.56015500	1.85449200

H	-5.82381400	-3.96895500	1.81908000
H	-4.15891900	-4.29325100	2.35020700
H	-4.82486500	-2.64624500	2.46016200
C	5.54064700	-2.40305200	-0.17176500
C	6.09596400	-1.15363800	0.03806300
C	5.25918800	-0.00717900	0.14838700
C	3.84047800	-0.17438400	0.03826300
C	3.29210700	-1.48125100	-0.17825100
C	4.14581300	-2.57902500	-0.28102600
H	6.85672200	1.43052500	0.44723000
H	6.18745600	-3.27360600	-0.25509000
H	7.17283900	-1.02974900	0.12043100
C	5.78068500	1.30000400	0.36368500
C	2.97907100	0.96558500	0.14467800
H	3.72477600	-3.56791600	-0.44559900
C	3.52883700	2.22893200	0.35576300
C	4.92658300	2.38323900	0.46345100
H	2.87058900	3.09062900	0.43603100
H	5.33389400	3.37831300	0.62791500
N	1.61042100	0.76977000	0.03217400
N	1.91708100	-1.60260500	-0.27806700
H	1.02653500	1.59766100	0.10541100
H	1.54754600	-2.53718100	-0.42747000
B	0.99386100	-0.50921900	-0.18058500
B	-1.50286600	0.50417500	-0.20768700
O	-1.04987200	1.81621700	-0.10835100
O	-2.88176500	0.44882300	-0.20812600
C	-2.19516700	2.66928700	0.19473000
C	-3.39158000	1.80727400	-0.34399500
C	-2.00770700	4.01166700	-0.49914800
H	-1.82926300	3.89139400	-1.57082600
H	-2.89534500	4.63940300	-0.36007800
H	-1.14908600	4.53560500	-0.06404200
C	-2.21809200	2.85702200	1.71404400
H	-3.02494900	3.53195600	2.01856500
H	-2.34646300	1.90235600	2.23506200
H	-1.26590500	3.29543400	2.03258100
C	-4.68043300	1.93096500	0.45852400
H	-5.04037300	2.96627500	0.44809100
H	-5.45457400	1.29749800	0.01087200
H	-4.54283100	1.61926900	1.49725000
C	-3.67470500	2.02932500	-1.83277700
H	-4.11765500	3.01491800	-2.01163800
H	-2.76233400	1.94284500	-2.43246800
H	-4.38258700	1.26723400	-2.17622600

22-ts

C	2.40902600	1.66206400	-0.79953800
O	2.98655300	1.77638800	-1.88551100
C	1.02785000	1.24067500	-0.63105100
C	0.61742100	-0.04351300	-0.39449300
O	2.96356100	2.11056300	0.36033700
C	-5.62626100	1.04039800	-0.21709200
C	-6.04665200	-0.25688100	0.01830300
C	-5.09305200	-1.30784700	0.12874900
C	-3.70156400	-0.99366100	-0.00702900
C	-3.29178900	0.35794700	-0.25280900
C	-4.26014400	1.35922200	-0.35389900
H	-6.52446900	-2.90223900	0.47209100
H	-6.36138400	1.83803200	-0.30061900
H	-7.10348400	-0.49034800	0.12093600
C	-5.46965400	-2.66000100	0.36889400
C	-2.72395700	-2.03380600	0.10152500
H	-3.94981600	2.38345800	-0.54095200
C	-3.13218300	-3.34615300	0.33714200
C	-4.50407300	-3.64558400	0.46845200
H	-2.38445200	-4.13147400	0.41882000
H	-4.80154500	-4.67566200	0.65196300
N	-1.38717400	-1.69261300	-0.03267900
N	-1.94217500	0.63350100	-0.38279500
H	-0.71966800	-2.45534500	0.03438200
H	-1.69552400	1.62123700	-0.54977500
B	-0.91314300	-0.35773900	-0.27711400
B	1.65626200	-1.19081300	-0.22738300
O	1.31918500	-2.53765400	-0.24157100
O	3.00991100	-1.01098500	-0.04162000
C	2.49247700	-3.30182700	0.17773900

C	3.66145200	-2.31031800	-0.16265900
C	2.52262000	-4.61722000	-0.58817300
H	2.47521900	-4.45867000	-1.66863000
H	3.43884200	-5.17211700	-0.35584900
H	1.66704600	-5.23567000	-0.29387300
C	2.33386100	-3.56376000	1.67744700
H	3.15523600	-4.17797300	2.06129400
H	2.30122500	-2.62874400	2.24664800
H	1.39494700	-4.10191400	1.84774700
C	4.84029900	-2.35899800	0.80035400
H	5.29998900	-3.35392800	0.79010900
H	5.59834100	-1.63047400	0.49147700
H	4.53659700	-2.12485500	1.82397400
C	4.15314400	-2.42844300	-1.60763400
H	4.70548400	-3.36111000	-1.76372100
H	3.32157300	-2.39216300	-2.31939400
H	4.82458300	-1.59058300	-1.82385800
C	-0.74448400	4.14602000	0.12819300
O	-0.77546600	3.15105400	-0.88452800
H	0.09629800	2.35348200	-0.75863200
C	0.59132800	4.91193300	0.07365200
H	0.72921600	5.35915900	-0.91909300
H	0.62561800	5.71632300	0.82095100
H	1.43227100	4.23529500	0.26371200
C	-1.90683100	5.11423500	-0.13308100
H	-2.86290800	4.57664400	-0.10493100
H	-1.94063100	5.91543800	0.61756800
H	-1.80327100	5.57466400	-1.12381100
C	-0.91439200	3.50486400	1.52096800
H	-0.10782200	2.78887400	1.71836400
H	-0.89861100	4.26417200	2.31432400
H	-1.86853300	2.96716900	1.58290700
C	4.28975600	2.67687100	0.27891100
C	4.69519800	3.09711400	1.67784200
H	4.27381700	3.52985400	-0.40790600
H	4.97247300	1.92266600	-0.12662100
H	5.70295700	3.52923400	1.65316300
H	4.00745200	3.85118200	2.07805500
H	4.70646400	2.23801000	2.35886400

23-ts

C	-0.72938000	-2.35774100	-0.87443700
O	-0.57502600	-2.82154800	-2.00521900
C	-1.26617100	-1.03848200	-0.55965800
C	-0.48999400	0.04150400	-0.25853400
O	-0.52596400	-3.12114000	0.24492100
C	5.30244000	-2.33814700	0.74226800
C	6.03347900	-1.20628800	0.43148800
C	5.37045000	-0.01072900	0.03492400
C	3.93965600	-0.00653500	-0.03631800
C	3.20897400	-1.19390500	0.28937900
C	3.89348100	-2.34374500	0.67500500
H	7.16221800	1.18122500	-0.23994900
H	5.81716800	-3.24724100	1.04463200
H	7.11915000	-1.21284900	0.48590100
C	6.07639200	1.18175600	-0.29127300
C	3.25166100	1.18697900	-0.42709600
H	3.33228000	-3.24165900	0.92187100
C	3.97743100	2.33395600	-0.73809100
C	5.38629800	2.31955000	-0.66688700
H	3.44858100	3.23642500	-1.03472200
H	5.93369900	3.22621500	-0.91423200
N	1.86474500	1.15559300	-0.48181700
N	1.82199600	-1.15661200	0.21384800
H	1.40635900	2.01651900	-0.76483500
H	1.34770500	-2.00579500	0.50160100
B	1.07280200	-0.00053300	-0.18241600
B	-1.13930800	1.43834400	-0.02693200
O	-0.73154800	2.55807200	-0.73652200
O	-2.06834100	1.74971900	0.93827100
C	-1.25636700	3.73681200	-0.04718100
C	-2.48003400	3.13875400	0.73495600
C	-1.61323700	4.79579800	-1.08081700
H	-2.29010100	4.40373800	-1.84418200
H	-2.09229200	5.65422000	-0.59615800
H	-0.70284400	5.15000200	-1.57750100
C	-0.13723000	4.24139100	0.86749900

H	-0.43325400	5.15826700	1.38765600
H	0.14035700	3.49005200	1.61428000
H	0.74700000	4.46319500	0.26001000
C	-2.73956700	3.77715100	2.09280700
H	-2.96066000	4.84418500	1.97463900
H	-3.60565300	3.30063900	2.56570900
H	-1.88190200	3.67096100	2.76233400
C	-3.76857300	3.10638500	-0.09021200
H	-4.16476500	4.11600700	-0.24194600
H	-3.60914800	2.64203800	-1.06871100
H	-4.52109100	2.51900600	0.44603200
C	-4.63033400	-1.77706400	-0.23116600
O	-3.85639000	-0.91497100	-1.02971200
H	-2.64408700	-0.98902100	-0.75493300
C	-0.15320800	-4.50775700	0.04463500
C	-0.01952500	-5.14375000	1.41353900
H	0.78988100	-4.54445100	-0.51061100
H	-0.92551800	-4.99775800	-0.55625200
H	0.26569500	-6.19664300	1.30094500
H	0.75191900	-4.64110200	2.00854900
H	-0.96780000	-5.10013300	1.96156600
C	-4.39326300	-1.49806600	1.27051800
H	-4.63516100	-0.45332200	1.50244900
H	-5.01163200	-2.14463600	1.90894700
H	-3.34195700	-1.66662200	1.53374300
C	-4.28651500	-3.25535000	-0.52627000
H	-4.91560200	-3.94668700	0.05219300
H	-4.43232600	-3.46868100	-1.59316900
H	-3.23987000	-3.46672800	-0.27834700
C	-6.11592900	-1.53649200	-0.55361900
H	-6.77547000	-2.18229600	0.04326600
H	-6.38194600	-0.49119200	-0.34926300
H	-6.31104800	-1.73476600	-1.61564800

24

C	-2.52774200	2.02070600	-1.37462800
O	-3.08727300	1.90093200	-2.47895400
C	-1.14532500	1.76520000	-1.09049000
C	-0.65795400	0.61655000	-0.53700200
O	-3.19360800	2.59714100	-0.31300600
C	-4.55395800	3.00806000	-0.53857400
H	-5.14027400	2.14950300	-0.88441500
H	-4.57904400	3.77287700	-1.32405500
C	-5.09498600	3.54809200	0.77224800
H	-6.13510200	3.87031100	0.63875300
H	-5.07160300	2.77815400	1.55279600
H	-4.51006000	4.40956200	1.11607200
C	5.31732900	-0.97316900	-1.91635000
C	5.92050800	-0.46012200	-0.78760000
C	5.13404700	0.16951700	0.21645000
C	3.71330700	0.26725800	0.03958400
C	3.11160600	-0.27049300	-1.14644100
C	3.92235400	-0.88566400	-2.10497400
H	6.80624100	0.62553000	1.51804400
H	5.91867300	-1.45588200	-2.68334100
H	6.99615700	-0.52797700	-0.64607200
C	5.72909000	0.70224900	1.39330300
C	2.91779600	0.89533800	1.05485800
H	3.49221400	-1.30138100	-3.00667300
C	3.54443300	1.40296500	2.19684500
C	4.94252000	1.30295300	2.35341300
H	2.96659000	1.88297600	2.97573100
H	5.39782400	1.71074500	3.25301200
N	1.53691200	0.97634300	0.86847900
N	1.72969000	-0.15814200	-1.30451100
B	0.88995100	0.48154800	-0.32132100
B	-1.54101200	-0.57494100	-0.08738500
O	-1.01986700	-1.63249400	0.64528600
O	-2.89061700	-0.73938600	-0.33301700
C	-2.04117100	-2.67057500	0.72119200
C	-3.35960300	-1.85041300	0.48559600
C	-1.95029600	-3.35830400	2.07728200
H	-1.99720800	-2.63935800	2.89957800
H	-2.76809200	-4.07846300	2.19589200
H	-1.00286600	-3.90408000	2.15287800
C	-1.74016900	-3.66990500	-0.39970900
H	-2.43279300	-4.51807600	-0.37529300

H	-1.80213500	-3.19745500	-1.38581200
H	-0.72264300	-4.05429300	-0.26870100
C	-4.44934000	-2.59720300	-0.27250800
H	-4.76307600	-3.48629900	0.28667700
H	-5.32324900	-1.94789300	-0.39782000
H	-4.11015600	-2.91007500	-1.26347100
C	-3.93131900	-1.24385900	1.77065400
H	-4.36239900	-2.01466800	2.41844400
H	-3.16342200	-0.70337800	2.33433800
H	-4.72313600	-0.53465000	1.50592400
C	0.75899200	1.57071800	1.95332500
H	1.03972700	2.61931400	2.11621400
H	-0.29698500	1.53108500	1.69597000
H	0.91746100	1.02223500	2.89050200
C	1.15440900	-0.73969500	-2.51524800
H	0.07745100	-0.58648000	-2.50657100
H	1.56605800	-0.26632400	-3.41583100
H	1.35868800	-1.81675900	-2.56683000

25-ts

C	2.59252500	1.72962100	-0.04119500
O	3.14296000	1.99738900	-1.11424600
C	1.21996300	1.27684400	0.10319900
C	0.80555400	-0.01712400	0.05698200
O	3.18014900	2.01090600	1.16004500
C	-4.89251500	-0.93795800	2.66505600
C	-5.60829800	-1.12619000	1.50172900
C	-4.94659000	-1.08345100	0.24347900
C	-3.53301900	-0.83996500	0.19939900
C	-2.81096000	-0.65501200	1.42510400
C	-3.50173100	-0.70530200	2.63922500
H	-6.72943400	-1.46452600	0.92894600
H	-5.39917000	-0.96896500	3.62693100
H	-6.67950400	-1.30949200	1.52572200
C	-5.65895700	-1.28031000	-0.97178700
C	-2.86535200	-0.79134600	-1.06922000
H	-2.98022000	-0.56313400	3.57653700
C	-3.60334500	-0.99779500	-2.23828500
C	-4.99166600	-1.23961900	-2.17761900
H	-3.12221600	-0.97111500	-3.20716300
H	-5.53582400	-1.39350800	-3.10663800
N	-1.49455400	-0.53180400	-1.09304900
N	-1.43484800	-0.43295200	1.36357100
B	-0.73510900	-0.31756100	0.11146900
B	1.76975700	-1.22640500	-0.04566900
O	1.31704600	-2.50399700	-0.32560200
O	3.13549600	-1.19000200	0.14413600
C	2.42913200	-3.42639700	-0.11788200
C	3.67426900	-2.48107200	-0.27274400
C	2.34154800	-4.54508400	-1.14773600
H	2.29162500	-4.15435300	-2.16749600
H	3.21192200	-5.20676700	-1.06947500
H	1.44197000	-5.14385900	-0.96551100
C	2.27284800	-3.99400800	1.29562400
H	3.04441700	-4.74047700	1.51228100
H	2.32839300	-3.20493800	2.05315500
H	1.29382000	-4.47867500	1.37767600
C	4.85743400	-2.83206800	0.61938400
H	5.22925600	-3.83554400	0.38225000
H	5.67179300	-2.11862500	0.44971900
H	4.58972500	-2.80029700	1.67874500
C	4.13592200	-2.31947900	-1.72368600
H	4.61156200	-3.23376100	-2.09438900
H	3.30019100	-2.06753500	-2.38511700
H	4.86828900	-1.50645700	-1.77393400
C	-0.86604800	3.93457200	-0.57418500
O	-0.64697700	3.08157200	0.52579000
H	0.25982900	2.28938700	0.29081700
C	4.46392600	2.67651500	1.13997500
C	4.30599900	4.18565500	1.04473300
H	5.06094700	2.28405100	0.31272500
H	4.93404300	2.38994200	2.08470000
H	5.29230800	4.66445700	1.09726900
H	3.83225900	4.47405100	0.10071800
H	3.69557400	4.56558900	1.87251600
C	-1.31282800	3.12039700	-1.80805500
H	-2.21993000	2.54893600	-1.57523500

H	-1.52410100	3.76731800	-2.67125200
H	-0.53142600	2.40967300	-2.09851100
C	0.42235700	4.71375700	-0.92340300
H	0.26580300	5.40977300	-1.75954100
H	0.75934800	5.29265300	-0.05358000
H	1.22675600	4.02316100	-1.20212900
C	-1.97369600	4.93382600	-0.20034900
H	-2.19037300	5.63046400	-1.02253600
H	-2.89953700	4.39888500	0.04828300
H	-1.67518200	5.52168900	0.67756300
C	-0.73205300	-0.28453400	2.63578500
H	0.33044400	-0.15175800	2.44427400
H	-1.09761800	0.58880400	3.19120100
H	-0.87054600	-1.17462000	3.26232600
C	-0.84926800	-0.50800800	-2.40318400
H	0.20603800	-0.27571000	-2.27813700
H	-0.94089100	-1.48193600	-2.90096000
H	-1.29806400	0.25460600	-3.05054300

26-ts

C	0.56030600	2.48053900	0.09725000
O	0.24271700	2.99644500	1.17451400
C	1.19799500	1.18246600	-0.07467400
C	0.49542700	0.02054500	-0.09337100
O	0.45480900	3.16646700	-1.08157300
C	-5.14552200	-0.68698000	2.68593600
C	-5.93762500	-0.44044400	1.58471300
C	-5.33990200	-0.14440700	0.32855300
C	-3.90952200	-0.10452600	0.22152100
C	-3.10952000	-0.36092100	1.38414900
C	-3.73823400	-0.64959100	2.59796500
H	-7.21590200	0.08132800	-0.73305000
H	-5.60325200	-0.91422800	3.64594500
H	-7.02193400	-0.46790000	1.65632000
C	-6.13312600	0.11269000	-0.82364000
C	-3.30575600	0.18963500	-1.04591300
H	-3.15500500	-0.84806700	3.48764200
C	-4.12325800	0.43481700	-2.15225500
C	-5.52796100	0.39497300	-2.02976200

H	-3.69108000	0.66095100	-3.11822100
H	-6.13460700	0.59203600	-2.91062200
N	-1.91146500	0.21661800	-1.13390900
N	-1.71943500	-0.30861400	1.26296400
B	-1.07291500	-0.01799800	0.01121400
B	1.22491700	-1.34694500	-0.25624200
O	1.33364500	-2.00092700	-1.46726000
O	1.74360800	-2.08840700	0.78380300
C	1.76020500	-3.37323400	-1.19828500
C	2.44042800	-3.24040200	0.21200100
C	2.68835300	-3.82816100	-2.31640700
H	3.52580100	-3.13881700	-2.45171500
H	3.08748800	-4.82570900	-2.09942000
H	2.13156900	-3.88218700	-3.25881300
C	0.49062300	-4.22854100	-1.18113700
H	0.72627600	-5.28842900	-1.03784800
H	-0.19494200	-3.91351000	-0.38709500
H	-0.02502200	-4.11820700	-2.14131500
C	2.24061400	-4.43781900	1.13202800
H	2.68090000	-5.33793000	0.68777600
H	2.73881600	-4.25304100	2.09043700
H	1.18194500	-4.62756800	1.32750700
C	3.92370200	-2.87224000	0.13698100
H	4.51658400	-3.70514400	-0.25553200
H	4.08801500	-1.99324900	-0.49391200
H	4.28519200	-2.63976300	1.14421700
C	4.53167500	1.65757800	0.62236200
O	3.80325400	1.12396700	-0.46060100
H	2.60628000	1.19427700	-0.24715500
C	0.00098900	4.53878200	-1.02826000
C	1.15347900	5.49607700	-0.77039400
H	-0.77515000	4.63315800	-0.26430100
H	-0.44426000	4.71567500	-2.01116000
H	0.78853900	6.53054900	-0.80807400
H	1.59837900	5.32315600	0.21480100
H	1.93399900	5.37931700	-1.53147200
C	4.26736500	0.84350400	1.90818600
H	4.55052500	-0.20515500	1.75699600

H	4.83605200	1.23457500	2.76372500
H	3.20167800	0.87070900	2.16608700
C	4.13911100	3.13184400	0.86904400
H	4.74005000	3.58576400	1.66953800
H	4.28685800	3.71837900	-0.04684700
H	3.08379700	3.20908100	1.15377400
C	6.02899700	1.58539700	0.27965500
H	6.65310700	1.97949600	1.09405700
H	6.32506100	0.54505200	0.09269700
H	6.24094800	2.16695600	-0.62706200
C	-0.93802400	-0.53812600	2.47675700
H	0.11968700	-0.43858900	2.24406300
H	-1.19738800	0.19524800	3.25052300
H	-1.11844500	-1.54342400	2.87790500
C	-1.33762200	0.47067900	-2.45416700
H	-0.25362200	0.44557700	-2.37937200
H	-1.66471700	-0.29203600	-3.17217800
H	-1.63937800	1.45516500	-2.83118600

IX. Single-crystal X-ray Diffraction

Crystal structure determination: Crystal of **2a**, **2e**, **2j**, and **2m** suitable for single-crystal X-ray diffraction were selected, coated in perfluoropolyether oil, and mounted on MiTeGen sample holders. Diffraction data of **2a** were collected on a BRUKER X8-APEX II diffractometer with a CCD area detector using graphite-monochromated Mo-K_α radiation. Diffraction data of **2e**, **2j**, and **2m** were collected on a Rigaku Oxford Diffraction XtaLAB Synergy diffractometer with a semiconductor HPA-detector (HyPix-6000) and multi-layer mirror monochromated Cu-K_α radiation. The crystals were cooled using Oxford Cryostream low-temperature devices. Data were collected at 100 K. The images were processed and corrected for Lorentz-polarization effects and absorption as implemented in the Bruker software packages (**2a**) or using the CrysAlisPro software from Rigaku Oxford Diffraction (**2e**, **2j**, **2m**). The structures were solved using the intrinsic phasing method (SHELXT)[11] and Fourier expansion technique. All non-hydrogen atoms were refined in anisotropic approximation, with hydrogen atoms 'riding' in idealized positions, by full-matrix least squares against F^2 of all data, using SHELXL[12] software and the SHELXLE graphical user interface.[13] The crystal of **2m** was a non-merohedral twin with domains rotated by 180.0° around real axis [100]. The twin fraction was refined to 43.7%. Diamond[14] software was used for graphical representation. Crystal data and experimental details are listed in Table S5. Full structural information has been deposited with the Cambridge Crystallographic Data Centre. CCDC-1959477 (**2a**), 1969050 (**2e**), 1969051 (**2j**), and 1969052 (**2m**).

Table S5: Single-crystal X-ray diffraction data and structure refinements of compounds **2a**, **2e**, **2j**, and **2m**.

Data	**2a**[a]	**2e**
CCDC number	1959477	1969050
Empirical formula	$C_{21}H_{26}B_2N_2O_4$	$C_{26}H_{28}B_2N_2O_4$
Formula weight / g·mol^{-1}	392.06	454.12
T / K	100(2)	100(2)
λ / Å, radiation	0.71073, Mo-Kα	1.45184, Cu-Kα
Crystal size / mm³	0.152×0.300×0.423	0.137×0.188×0.255
Crystal color, habit	colorless plate	yellow block
μ / mm^{-1}	0.084	0.675
Crystal system	Monoclinic	Monoclinic
Space group	$P2_1/c$	$P2_1/c$
a / Å	11.033(3)	8.20510(10)
b / Å	8.345(2)	14.59160(10)
c / Å	22.965(8)	20.20720(10)
α / °	90	90
β / °	99.702(19)	101.5190(10)
γ / °	90	90
Volume / Å^3	2084.2(11)	2370.59(4)
Z	4	4
ρ_{calc} / g·cm^{-3}	1.249	1.272
F(000)	832	960
θ range / °	2.601 – 26.767	3.763 – 74.463
Reflections collected	22419	25622
Unique reflections	4432	4840
Parameters / restraints	267 / 0	312 / 0
GooF on F^2	1.026	1.031
R_1 [I>2σ(I)]	0.0442	0.0339
wR^2 (all data)	0.1097	0.0882
Max. / min. residual electron density / e·Å^{-3}	0.296 / –0.252	0.331 / –0.221

[a] For compound **2a**, the unit-cell parameters and their standard deviations were obtained from a combination of the random-error standard deviations from the original least-squares refinement with the systematic-error component estimated from 32 Monte-Carlo simulations using the Bruker software package. For this reason, the standard deviations of the unit-cell parameters are significantly larger for **2a** than for **2e**, **2j** and **2m**, and they represent a better overall estimation of the realistic standard deviations.

Table S5: Continued.

Data	**2j**	**2m**
CCDC number	1969051	1969052
Empirical formula	$C_{26}H_{32}B_2N_2O_4$	$C_{26}H_{29}B_2N_3O_3$, CH_2Cl_2
Formula weight / g·mol^{-1}	458.15	538.07
T / K	100(2)	100(2)
λ / Å, radiation	1.45184, Cu-Kα	1.45184, Cu-Kα
Crystal size / mm^3	0.173×0.197×0.286	0.044×0.078×0.405
Crystal color, habit	yellow block	yellow needle
μ / mm^{-1}	0.629	2.352
Crystal system	Monoclinic	Triclinic
Space group	$P2_1/c$	$P\bar{1}$
a / Å	10.55440(10)	13.3012(3)
b / Å	14.1696(2)	14.1175(4)
c / Å	17.6434(2)	15.4831(4)
α / °	90	78.036(2)
β / °	105.1800(10)	82.282(2)
γ / °	90	81.451(2)
Volume / Å^3	2546.53(5)	2796.52(13)
Z	4	4
ρ_{calc} / g·cm^{-3}	1.195	1.278
F(000)	976	1128
θ range / °	4.059 – 74.483	2.934 – 74.489
Reflections collected	27178	72498
Unique reflections	5192	12632
Parameters / restraints	372 / 0	749 / 91
GooF on F^2	1.058	1.025
R_1 [I>2σ(I)]	0.0389	0.0674
wR^2 (all data)	0.1074	0.1861
Max. / min. residual electron density / e·Å^{-3}	0.262 / –0.216	0.646 / –0.637

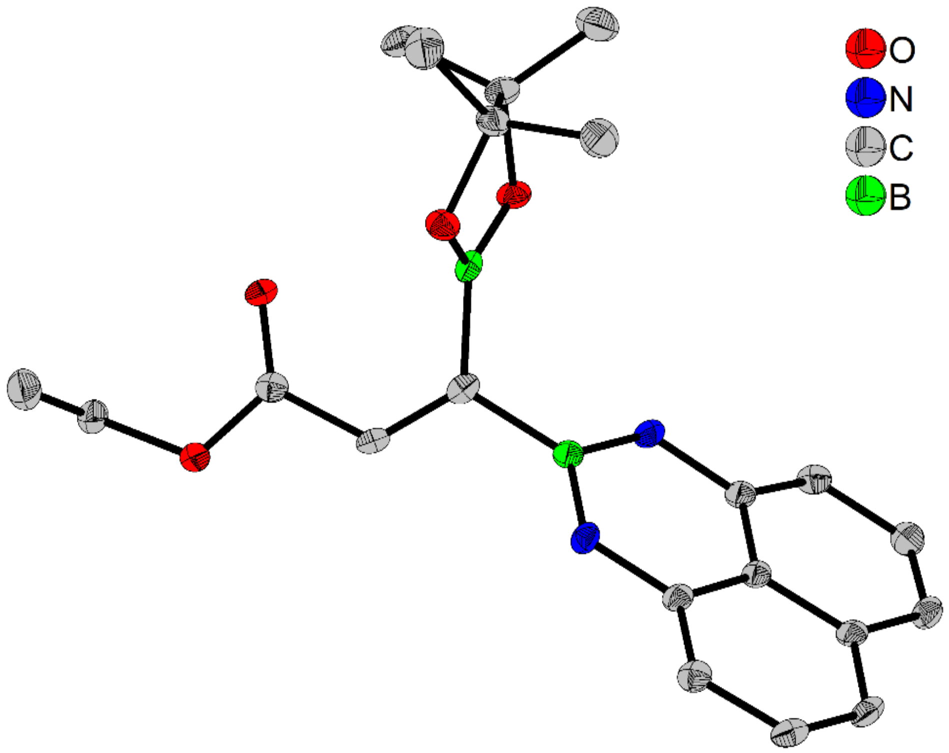

Figure S9. Molecular structure of **2a** in the solid state at 100 K. Atomic displacement ellipsoids are drawn at the 50% probability level, and H atoms are omitted for clarity.

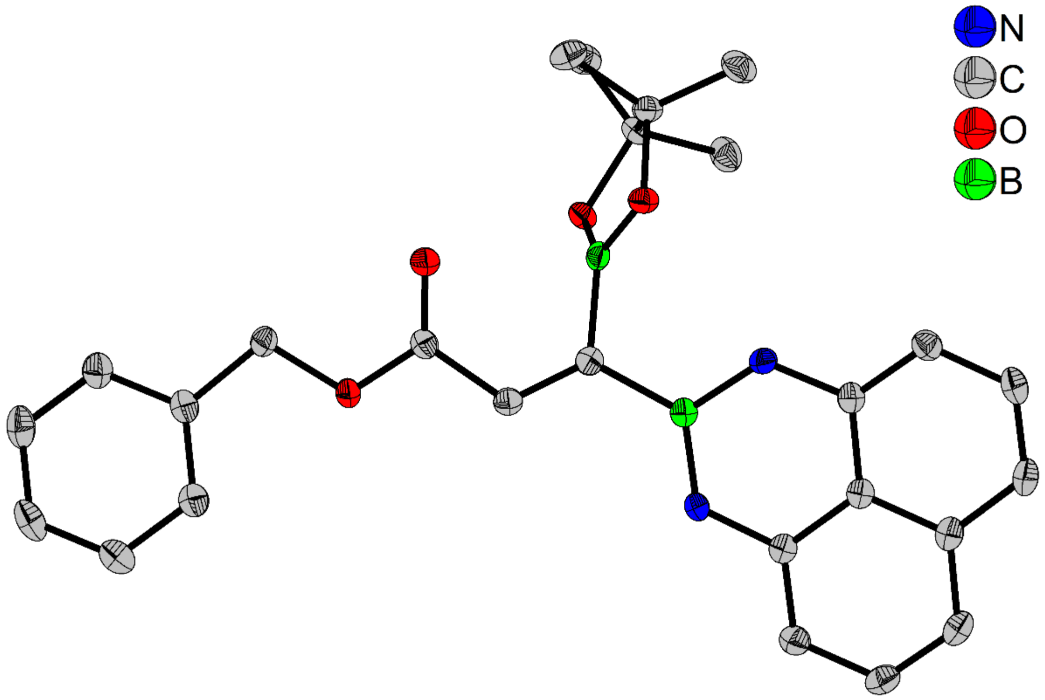

Figure S10. Molecular structure of **2e** in the solid state at 100 K. Atomic displacement ellipsoids are drawn at the 50% probability level, and H atoms are omitted for clarity.

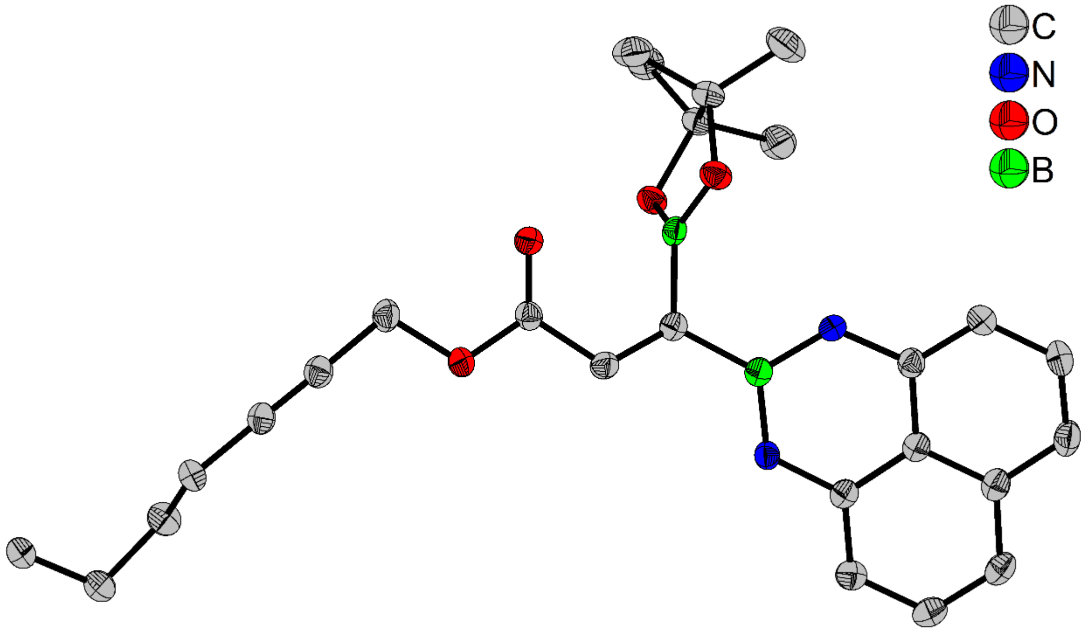

Figure S11. Molecular structure of **2j** in the solid state at 100 K. Atomic displacement ellipsoids are drawn at the 50% probability level, and H atoms are omitted for clarity. The hept-2-yn-1-yl moiety is disordered and only the part with the higher occupancy (75%) is shown here.

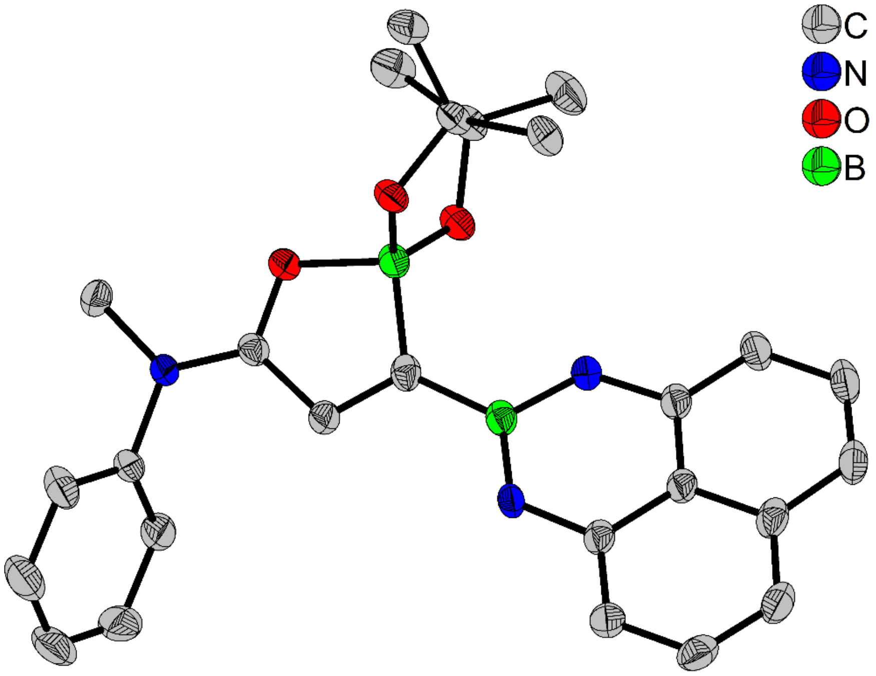

Figure S12. Molecular structure of **2m** in the solid state at 100 K. Atomic displacement ellipsoids are drawn at the 50% probability level, and H atoms as well as solvent molecules are omitted for clarity. Only one of two symmetrically non-equivalent molecules is shown here.

Table S6: O···B distance from the carbonyl oxygen atom to the boron atom of the Bpin moiety in **2a**, **2e**, **2j**, and **2m**. In compound **2m** there are two independent molecules in the unit cell.

compound	O···B
**2a**	2.760(2)
**2e**	2.5881(12)
**2j**	2.5370(14)
**2m**	1.642(3) / 1.628(4)

X. References

[1] H. Yoshida, Y. Takemoto, K. Takaki, *Chem. Commun.* **2014**, *50*, 8299-8302.

[2] A. López, T. B. Clark, A. Parra, M. Tortosa, *Org. Lett.* **2017**, *19*, 6272-6275.

[3] A. G. Aioub, C. J. Higginson, M. G. Finn, *Org. Lett.* **2018**, *20*, 3233-3236.

[4] K. Okamoto, T. Hayashi, V. H. Rawal, *Chem. Commun.* **2009**, 4815-4817.

[5] L. Feray, M. P. Bertrand, *Eur. J. Org. Chem.* **2008**, *2008*, 3164-3170.

[6] M. J. Frisch, G. W. Trucks, H. B. Schlegel, G. E. Scuseria, M. A. Robb, J. R. Cheeseman, G. Scalmani, V. Barone, B. Mennucci, G. A. Petersson, H. Nakatsuji, M. Caricato, X. Li, H. P.Hratchian, A. F. Izmaylov, J. Bloino, G. Zheng, J. L. Sonnenberg, M. Hada, M. Ehara, K. Toyota, R. Fukuda, J. Hasegawa, M. Ishida, T. Nakajima, Y. Honda, O. Kitao, H. Nakai, T. Vreven, J. A. J. Montgomery, J. E. Peralta, F. Ogliaro, M. Bearpark, J. J. Heyd, E. Brothers, K. N. Kudin, V. N. Staroverov, R. Kobayashi, J. Normand, K. Raghavachari, A. Rendell, J. C. Burant, S. S. Iyengar, J. Tomasi, M. Cossi, N. Rega, J. M. Millam, M. Klene, J. E. Knox, J. B. Cross, V. Bakken, C. Adamo, J. Jaramillo, R. Gomperts, R. E. Stratmann, O. Yazyev, A. J. Austin, R. Cammi, C. Pomelli, J. W. Ochterski, R. L. Martin, K. Morokuma, V. G. Zakrzewski, G. A. Voth, P. Salvador, J. J. Dannenberg, S. Dapprich, A. D. Daniels, O. Farkas, J. B. Foresman, J. V. Ortiz, J. Cioslowski, D. J. Fox, Gaussian 09, Revision D.01; Gaussian, Inc.: Wallingford, CT, **2009**.

[7] a) C. Lee, W. Yang, R. G. Parr, *Phys. Rev. B* **1988**, *37*, 785-789; b) A. D. Becke, *J. Chem. Phys.* **1993**, *98*, 5648-5652; c) P. J. Stephens, F. J. Devlin, C. F. Chabalowski, M. J. Frisch, *J. Phys. Chem.* **1994**, *98*, 11623-11627.

[8] A. V. Marenich, C. J. Cramer, D. G. Truhlar, *J. Phys. Chem. B* **2009**, *113*, 6378-6396.

[9] a) K. Fukui, *J. Phys. Chem.* **1970**, *74*, 4161-4163; b) K. Fukui, *Acc. Chem. Res.* **1981**, *14*, 363-368.

[10] R. Peverati, D. G. Truhlar, *J. Phys. Chem. Lett.* **2011**, *2*, 2810-2817.

[11] G. Sheldrick, *Acta Crystallogr.* **2015**, *A71*, 3-8.

[12] G. Sheldrick, *Acta Crystallogr.* **2008**, *A64*, 112-122.

[13] C. B. Hubschle, G. M. Sheldrick, B. Dittrich, *J. Appl. Crystallogr.* **2011**, *44*, 1281-1284.

[14] Brandenburg, K. Diamond (version 4.4.0), Crystal and Molecular Structure Visualization, Crystal Impact, H. Putz & K. Brandenburg GbR, Bonn (Germany), **2017**.
